# Microevolution of *cis*-Regulatory Elements: An Example from the Pair-Rule Segmentation Gene *fushi tarazu* in the *Drosophila melanogaster* Subgroup

**DOI:** 10.1371/journal.pone.0027376

**Published:** 2011-11-03

**Authors:** Mohammed Bakkali

**Affiliations:** Departamento de Genética, Facultad de Ciencias, Universidad de Granada, Granada, Spain; Ecole Normale Supérieure de Lyon, France

## Abstract

The importance of non-coding DNAs that control transcription is ever noticeable, but the characterization and analysis of the evolution of such DNAs presents challenges not found in the analysis of coding sequences. In this study of the *cis*-regulatory elements of the pair rule segmentation gene *fushi tarazu* (*ftz*) I report the DNA sequences of *ftz*'s zebra element (promoter) and a region containing the proximal enhancer from a total of 45 fly lines belonging to several populations of the species *Drosophila melanogaster*, *D. simulans*, *D. sechellia*, *D. mauritiana*, *D. yakuba*, *D. teissieri*, *D. orena* and *D. erecta*. Both elements evolve at slower rate than *ftz* synonymous sites, thus reflecting their functional importance. The promoter evolves more slowly than the average for *ftz*'s coding sequence while, on average, the enhancer evolves more rapidly, suggesting more functional constraint and effective purifying selection on the former. Comparative analysis of the number and nature of base substitutions failed to detect significant evidence for positive/adaptive selection in transcription-factor-binding sites. These seem to evolve at similar rates to regions not known to bind transcription factors. Although this result reflects the evolutionary flexibility of the transcription factor binding sites, it also suggests a complex and still not completely understood nature of even the characterized *cis*-regulatory sequences. The latter seem to contain more functional parts than those currently identified, some of which probably transcription factor binding. This study illustrates ways in which functional assignments of sequences within *cis*-acting sequences can be used in the search for adaptive evolution, but also highlights difficulties in how such functional assignment and analysis can be carried out.

## Introduction

The evolution of DNA sequences can be studied at various time scales. One level of study, referred to as microevolutionary, analyzes closely related species and often includes assessment of polymorphisms within species. It is the only way to identify the individual mutations in rapidly-evolving DNAs. In addition, the use of model organisms and their close relatives allows our knowledge of the biology of model species to be used in the interpretation of the evolutionary observations. Of particular interest is the study of microevolutionary changes in sequences controlling transcription. This approach has been applied to *Drosophila* species, as in the case of the *cis*-acting sequences of the *achaete-scute* gene complex [1], the *even-skipped* stripe 2 enhancer [2], [3], [4], and *Ultrabithorax* enhancers [5], [6].

The case of the *shavenbaby* gene in *Drosophila* species shows how evolutionary changes in some phenotypic traits are not necessarily due to changes in gene or protein sequences, but they are rather due to changes in gene expression regulation ([7], [8], [9], [10]). The evolution of transcription regulation is thus one of the intriguing topics in evolutionary biology (*e.g.*, see [11]). Evolutionary changes in a gene expression pattern can result from changes in genes acting on it *in trans* or from changes in its *cis*-acting regulatory elements (for a review see [12]). Regulatory elements (*e.g.*, see [13], [14], [15], [16], [17]) include promoters, which provide a binding site for TATA-binding protein and, through it, RNA polymerase II, and enhancers, which are typically further from the origin of transcription. Both promoters and enhancers contain binding sites for *trans*-acting proteins called transcription factors, both activators and repressors. They consist of an alternation of sites that bind to transcription factors and sites that do not.

Simultaneous comparison of sequence variation within and between species allows identification of selective constraint, as reflected by sequence conservation, as well as adaptive changes. Driven by selection, adaptive changes differ from neutral and deleterious changes by quickly spreading through populations. They will therefore be underrepresented as polymorphisms relative to fixed differences between species, when this ratio is compared to that for neutrally evolving bases. This allows statistical testing for adaptive evolution based on the division of the sequence into two classes; one subjected to neutral changes only, while the other evolves adaptively. In coding sequences, this dichotomy is between synonymous and replacement sites. It is unambiguous and forms the basis of the McDonald-Kreitman test [18].

Division of non-coding DNAs into two classes of sites is usually impossible. So Andolfatto [19] compared sequence polymorphism and divergence between non-coding DNAs and synonymous sites to successfully detect unequivocal signs of selective constraint and adaptive evolution on non-coding DNAs —which he concluded must contain regulatory elements and other functional DNAs. However, not all non-coding DNA sites are selectively constrained, and comparison with synonymous sites does not allow differentiation between constrained and unconstrained regions of a non-coding sequence. Nonetheless, regulatory elements —typically non-coding— offer an obvious dichotomy between bases binding to transcription factors and those not doing so. This allows for the application of the adaptation of the McDonald-Kreitman test suggested in [3], [20] to look for signs of adaptive selection on a regulatory element's TFBSs.

**Figure 1:**
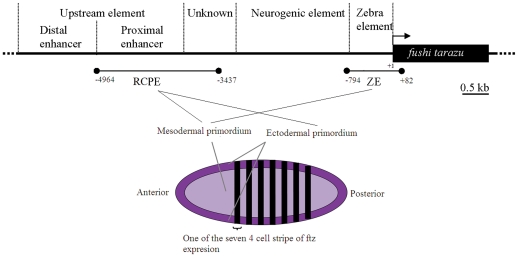
Location of the regions sequenced within the *ftz cis*-regulatory sequence (adapted from [29], [30], [31], [32]). The start and end positions of the regions analyzed in this work, relative to *ftz* start codon, are shown underneath the sequence diagram. The schematic representation underneath is of a blastoderm stage *D. melanogaster* embryo showing *ftz* expression pattern and the actuation domains of its zebra element and proximal enhancer. The ∼500 bp sequence between the proximal and the neurogenic enhancers, marked as unknown, is an uncharacterized part of the *ftz cis*-regulatory sequence.

Here I present data on the microevolution of the regulatory elements of the developmental pair rule segmentation gene *fushi tarazu* (*ftz*) within and among populations of *Drosophila* species. Activated by *caudal* and *runt*, repressed by *hairy* and *tramtrack*, and with a positive feedback on itself [21], [22], [23], [24]), *ftz* controls the expression of at least 11 developmental genes [25]. It is the only member of the Hox cluster that functions as a pair rule gene. This ancestrally *Hox* gene is one of the most evolutionarily flexible early development genes [26]. It has lost its homeotic function and is involved in the development of the central nervous system (CNS) in most metazoans, while also acting as a segmentation gene in *Drosophila*, *Anopheles gambiae* [27], and probably all mandibulate arthropods [28]. Its 6.1 kb *cis*-regulatory sequence [29], [30], [31], [32] consists of a proximal region, called the zebra element (∼0.74 kb), that contains the promoter and drives *ftz* expression in the mesodermal *primordium*, then a more complex upstream region of enhancers (Figure 1). The latter includes a neurogenic element (∼1.9 kb), involved in *ftz* expression in the developing CNS, an uncharacterized ∼1 kb sequence, and an upstream element (∼2.4 kb) containing two enhancers (proximal and distal). The proximal enhancer directs *ftz* expression in both ectodermal and mesodermal *primordia* whilst, like the zebra element, the action of the distal enhancer is mesodermally restricted.

I analyze sequence variability of a region containing the proximal enhancer (RCPE) and another containing the zebra element (ZE) within and between *Drosophila* species. The aim is toreveal the types of selection acting on these sequences. Given that the segmental expression of *ftz* is better characterized than its neurogenic expression (see references herein), I excluded *ftz* ´s neurogenic element to focus on the RCPE and ZE. The choice of one promoter and one enhancer allows comparative analysis of the evolutionary dynamics between these two types of *cis*-regulatory elements. For cost effectiveness I choose to include more samples (flies and species) than elements so, between the proximal and the distal enhancers, I favored the first as it controls *ftz* expression both in the ecto- and mesodermal *primordia*, whereas the latter drives *ftz* expression ‘only’ in the mesodermal *primordium*.

I identify regions that are functionally constrained in the sequences containing *ftz* proximal enhancer and zebra element and I test for signs of adaptive selection. I examine several lines from different populations of *D. melanogaster*, *D. simulans* and *D. yakuba*. In addition, single lines from *D. sechellia*, *D. mauritiana*, *D. teissieri*, *D. orena* and *D. erecta* were used for further inter-specific analyses.

## Results

### Comparison of sequence variability

**Figure 2:**
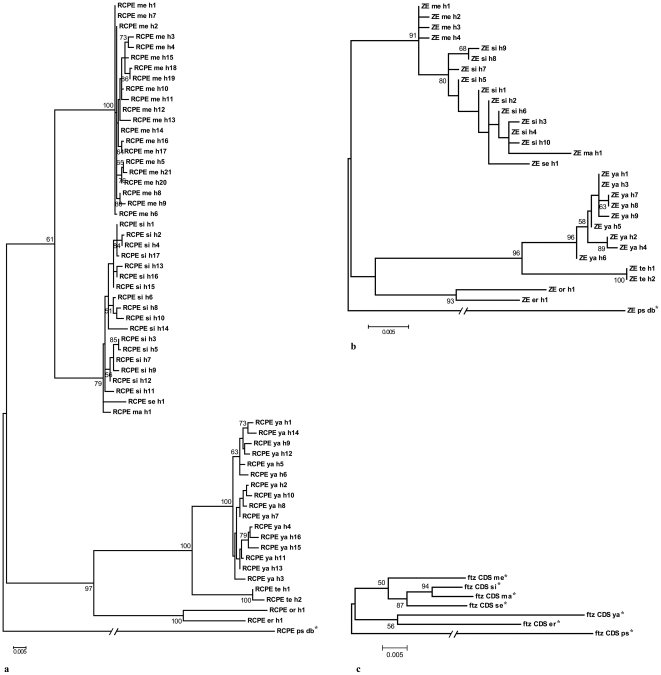
Maximum likelihood phylogenetic trees of the *ftz* RCPE (a), ZE (b) and CDS (c), constructed using PHYLIP. h1, h2…: haplotype 1, haplotype 2…, me: *D. melanogaster*, si: *D. simulans*, se: *D. sechellia*, ma: *D. mauritiana*, ya: *D. yakuba*, te: *D. teissieri*, or: *D. orena*, er: *D. erecta*, ps: *D. pseudoobscura*.*: The *D. pseudoobscura* sequences that served as outgroup and the *ftz* CDS sequences are from the database (accession numbers NM_058150.2 for *D. melanogaster* CDS, XM_002102380.1 for *D. simulans* CDS, EU670514.1 for *D. sechellia* CDS, EU310327.1 for *D. mauritiana* CDS, XM_002096692.1 for *D. yakuba* CDS, XM_001979089.1 for *D. erecta* CDS, XM_001359177.2 for *D. pseudoobscura* CDS, and AY190944 for *D. pseudoobscura* RCPE and ZE). For better visualization of the species and haplotype branches, the branch length of the outgroup in the *ftz* RCPE tree is compressed four times, that in the ZE tree eight times and that in the CDS tree compressed ten times. The scales reflect the genetic distance in substitution per site and only bootstrap values higher than 50 are shown.

From 45 fly lines, PHASE identified 55 RCPE and 29 ZE haplotypes (sequences in Figure S1 and GenBank accession numbers HQ693575- HQ693658). This reflects the higher variability of the RCPE as compared to the ZE, and how the two alleles in an individual are not completely independent samples from the population of origin due to some inbreeding, which also causes some single flies to carry the same rare allele in both *loci*. Figure 2 shows that, overall, the RCPE and ZE phylogenetic trees are similar and in agreement with the known phylogeny of the three major branches (*i.e.*, one containing *D. melanogaster*, *D. simulans*, *D. sechellia* and *D. mauritiana*, another containing *D. yakuba* and *D. teissieri* and a third containing *D. orena* and *D. erecta*).

**Figure 3:**
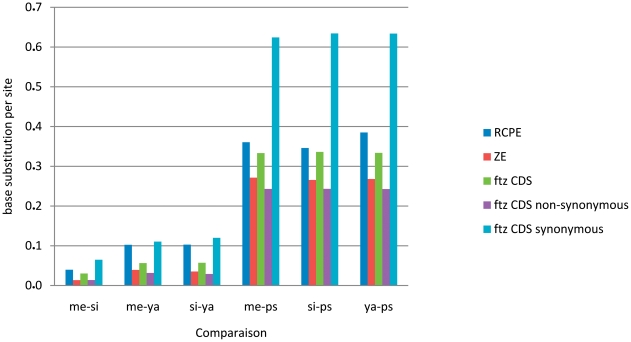
Base substitution per site for the sequences of the RCPE, ZE and *ftz* CDS in comparisons between *D. melanogaster* and *D. simulans* (me-si), *D. melanogsater* and *D. yakuba* (me-ya), *D. simulans* and *D. yakuba* (si-ya), *D. melanogaster* and *D. pseudoobscura* (me-ps), *D. simulans* and *D. pseudoobscura* (si-ps), and *D. yakuba* and *D. pseudoobscura* (ya-ps). Analyses were conducted using the software MEGA5 [88] following the Maximum Composite Likelihood model [89]. *: The *D. pseudoobscura* and *ftz* CDS sequences are from the database (accession numbers NM_058150.2 for *D. melanogaster* CDS, XM_002102380.1 for *D. simulans* CDS, EU670514.1 for *D. sechellia* CDS, EU310327.1 for *D. mauritiana* CDS, XM_002096692.1 for *D. yakuba* CDS, XM_001979089.1 for *D. erecta* CDS, XM_001359177.2 for *D. pseudoobscura* CDS, and AY190944 for *D. pseudoobscura* RCPE and ZE).

**Figure 4:**
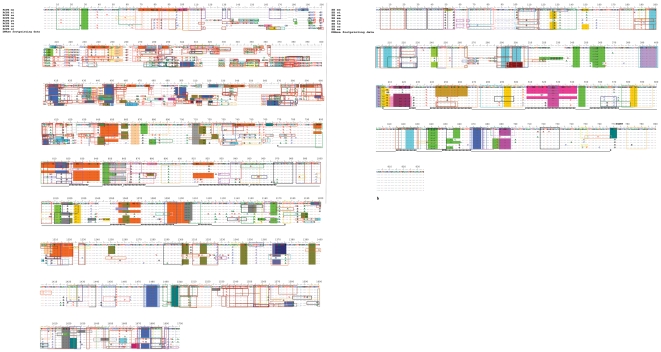
Sequence alignment and location of the TFBSs on the RCPE (a) and ZE (b) of the eight *Drosophila* species studied in this work. Shaded positions are those indentified by PATCH searches and boxes delimit TFBSs are those identified by MATCH searches (see material and methods). me: *D. melanogaster*, si: *D. simulans*, se: *D. sechellia*, ma: *D. mauritiana*, ya: *D. yakuba*, te: *D. teissieri*, or: *D. orena*, er: *D. erecta*. Underlined: Nucleotides shared between two different transcription factor-binding regions identified by PATCH. Double underlined: nucleotides shared between three different transcription factor-binding regions identified by PATCH. Strikethrough: Nucleotides at the core sequence of a TFBS identified by MATCH. DNase-I footprinting data are those in references [29], [39], [40] of the manuscript. S: Start of the DNase-I footprinted sequence. E: End of the DNase-I footprinted sequence. P: Experimentally tested transcription factor binding position. START marks *ftz* transcription start. The color codes for the transcription factor binding sites are those in Figure S1. Positions of the alignments that are in italic and not in bold represent regions of the sequence where the species is polymorphic for an insertion or deletion.

**Figure 5:**
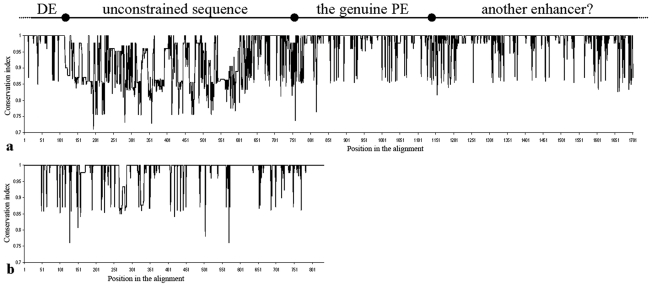
Sequence conservation of the RCPE (a) and ZE (b) from all the lines of the eight *Drosophila* specie used in this work. Above a is a tentative inference of potentially functional parts of the sequences based on an interpretation of the sequence conservation along this phylogenetic footprinting. The start and end positions of the inferred functional elements are approximates and not exact. DE: distal enhancer. The conserved region that I interpret as possibly the functional proximal enhancer coincides with the minimal proximal enhancer suggested in [29], [30].

The trees clearly show that the RCPE is more variable than the ZE as it shows more haplotypes and higher sequence divergence; as inferred from the cumulative lengths of the phylogenetic branches separating the current species (Table 1). Indicative of their functional importance, both the RCPE and the ZE diverge at lower rate than that of the synonymous sites of the *ftz* coding sequence (CDS) and this tendency remains consistent even when using the more diverged *D. pseudoobscura* sequence (Table 1). Surprisingly, the ZE even shows significantly lower variability than the average across replacement and synonymous sites for the *ftz* CDS (Figure 3), thus reflecting the functional importance of gene promoters. For both elements, sequences of *D. melanogaster* vary least within species, while *D. simulans* and *D. yakuba* are most variable for the ZE and the RCPE, respectively (Table 2). Overall, sequences are shorter in size in the clade *D. melanogaster-D. simulans-D. sechellia-D. mauritiana* (ZE = 799 bp averaged across the four species, RCPE = 1448 bp), than in the clade *D. yakuba*-*D. teissieri*-*D. orena*-*D. erecta* (ZE = 814 bp, RCPE = 1514 bp). For the RCPE, this difference is mostly due to deletions at the region between positions 100 and 600 of the alignment, whereas the larger ZE sequences of *D. yakuba*, *D. teissieri, D. orena*, and *D. erecta* result from small duplications at positions 265 to 287 and 324 to 335 (Figure 4, Figure 5 and Figure S1). For *D. melanogaster* and *D. simulans*, sequence diversity is higher in African populations than in European ones, both for RCPE and ZE (Table 3), probably due to bottlenecks in the evolutionary history of the European populations. A similar result was reported for *D. melanogaster* in [33], [34], [35], [36], [37].

**Table 1:** Sequence divergence, in 10-3x substitution per site, as inferred from the cumulative lengths of the branches separating the current *Drosophila* species in the phylogenies of the *ftz* RCPE, ZE and CDS in Figure 3.

Element	*D. melanogaster- D. simulans*	*D. melanogaster- D. yakuba*	*D. simulans-D. yakuba*	*D. melanogaster- D. pseudoobscura*	*D. simulans -D. pseudoobscura*	*D. yakuba-D. pseudoobscura*
RCPE	53.338	143.871	142.284	486.667	485.081	537.003
ZE	9.487	40.211	47.828	286.776	294.393	308.299
*ftz* CDS	31.000	59.300	60.500	460.200	461.400	449.300

In the case of species with multiple haplotypes, the branch lengths were averaged.

**Table 2:** Comparison and test for deviation from neutrality of the RCPE and ZE sequence variability among species.

Element	Species	Total sites	Sites excluding gaps	Nucleotide variability/site		Neutrality tests	
				*Π*±S.D. x10^-3^	*Θ-W* x10^-3^	Tajima's *D*	Fu and Li's *D*
RCPE	*D. melanogaster*	1702	1433	4.71±0.32	5.99	−0.864, *p*>0.1	−0.745, *p*>0.1
	*D. simulans*		1463	7.31±0.51	5.62	1.105, *p*>0.1	**1.730, ** ***p*** **<0.02**
	*D. yakuba*		1482	8.66±0.56	7.59	0.554, *p*>0.1	0.844, *p*>0.1
ZE	*D. melanogaster*	834	803	0.7±0.15	0.94	−0.596, *p*>0.1	−0.263, *p*>0.1
	*D. simulans*		799	3.51±0.34	2.57	1.128, *p*>0.1	1.327, 0.1>*p*>0.05
	*D. yakuba*		823	1.75±0.42	2.33	−0.805, *p*>0.1	0.0075, *p*>0.1

*Π*: Average number of nucleotide differences per site between two sequences (equations 10.5 or 10.6 in [90]), its standard deviation is the square root of its sampling variance (equation 10.7 in [90]). *Θ-W*: Watterson estimator of variability (equation 1.4a in [91]) per site (equation 10.3 in [90]). Tajima's *D*: Suggested in [92] for testing whether all variation is selectively neutral. Fu and Li's *D*: Suggested in [93] for testing whether all variation is selectively neutral. Bold: Significant values.

**Table 3:** Comparison of RCPE and ZE sequence polymorphism between populations.

Element	Species	Population	Sequences (*h*, *hd*)	Total sites	Sites excluding gaps	Nucleotide variability/site		Neutrality tests	
						*π*±S.D. x10^−3^	*θ* x10^−3^	Tajima's *D*	Fu and Li's *D*
RCPE	*D. melanogaster*	Gabon	10 (8, 0.956)	1702	1433	4.25±0.59	5.18	−0.848, *p*>0.1	0.711, *p*>0.1
		Netherlands	10 (6, 0.844)		1488	4.21±0.64	3.80	0.500, *p*>0.1	0.799, *p*>0.1
	*D. simulans*	France	10 (6, 0.889)		1464	6.06±0.62	4.83	1.195, *p*>0.1	0.764, *p*>0.1
		Gabon	10 (6, 0.844)		1463	8.66±0.89	7.25	0.934, *p*>0.1	1.189, *p*>0.1
	*D. yakuba*	Cameroon	10 (7, 0.933)		1491	8.20±0.72	6.64	1.124, *p*>0.1	1.301, 0.1>*p>*0.05
		Gabon	10 (8, 0.956)		1484	8.42±0.87	7.38	0.672, *p*>0.1	0.942, *p*>0.1
ZE	*D. melanogaster*	Gabon	10 (4, 0.733)	834	803	1.13±0.27	1.32	−0.507, *p*>0.1	0.175, *p*>0.1
		Netherlands	10 (2, 0.533)		803	0.66±0.12	0.44	1.303, *p*>0.1	0.804, *p*>0.1
	*D. simulans*	France	10 (5, 0.756)		799	2.20±0.51	2.21	−0.027, *p*>0.1	−0.024, *p*>0.1
		Gabon	10 (6, 0.911)		799	4.09±0.41	3.10	1.356, *p*>0.1	0.880, *p*>0.1
	*D. yakuba*	Cameroon	10 (6, 0.889)		825	1.37±0.39	1.71	−0.762, *p*>0.1	−1.127, *p>*0.1
		Gabon	10 (5, 0.867)		823	1.81±0.56	1.72	0.204, *p*>0.1	0.450, *p*>0.1

All calculations are as in Table 2.

Tajima's *D* and Fu and Li's *D* tests show no significant deviations from neutrality, save for the significantly positive Fu and Li's *D* value of *D. simulans* RCPE sequences (Table 2), normally indicative of population subdivision. However, the significant Fu and Lís *D* value vanishes after correction for multiple testing. In addition, the fly lines analyzed in this work were not derived from a single population and, as noted above, flies from the same species and population seem to have undergone some inbreeding. These would invalidate the panmixia and random mating requirements for the neutrality tests.

Figure 5 shows that most changes in the RCPE are located within the ∼1 kb region identified as part of the *ftz* proximal enhancer [31], [32], [38] meaning that the higher variability of the RCPE does not result from the inclusion of the uncharacterized ∼0.5 kb sequence between *ftz*'s proximal and neurogenic enhancers. In fact, the uncharacterized sequence is less variable than the distal region of the RCPE and shows similar conservation index values to those shown by the ZE, suggesting that it is functionally constrained (Figure 5). Variability within the ZE is scattered along the sequence, and is mostly due to single nucleotide substitutions, while most of the variability within the RCPE is located between positions 100 and 600 of the aligned sequences and includes duplications/deletions of multiple bases (Figure 4, Figure 5 and Figure S1).

### Search for signs of adaptive selection

Both the ZE and the RCPE of the *ftz* gene have been previously tested for transcription factor binding by the use of the DNAse-I footprinting, and a number of regions have been identified as containing sites that bind transcription factors coded by genes with known effect on *ftz* expression; such as *tramtrack*, *ftz*, *caudal*, *trithorax-like* (see [29], [39], [40]). Here, I add a computer search for TFBSs.

Experimentally-detected elements have also been highlighted by the computer-based analyses used in the current work (see Figure 4 and Figure S1) suggesting the absence of significant false negative issues. On the other hand, all the sites identified *in silico* have been scrutinized via an extensive literature search to exclude potential false positives (see Table S3).

In addition to the experimentally-identified sites, the computer-based searches for known transcription factor binding sites in the insects directory of the TRANSFAC® 6.0 database identified the plethora of transcription factor binding sites summarized in Table S3. These include sites corresponding to general transcription factors such as B-Factor (TATA), Trithorax-Like Factor (GAGAG), Zeste and Boundary Element Associated Factor, as well as sites for all the transcription factors coded by genes known to regulate *ftz* activity (such as *even-skipped*, *engrailed*, *hairy*, *ftz*, *tramtrack* and *caudal*). In addition, I identified binding sites for transcription factors coded by developmental genes which interaction with *ftz* is probable, given what we know about *ftz* and *Drosophila* ´s development, but was not previously reported (*i.e.*, not experimentally demonstrated). Examples include *Abdominal-B*, *Antennapedia*, *Bicoid*, *pangolin* and Activating Protein 1 (see Table S3). Among these, binding sites for transcription factors coded by genes which functions include involvement in metamorphosis are to highlight. It is true that none of the factors identified is strictly a metamorphosis one and that *ftz* regulation by the factors which function include involvement in metamorphosis could be restricted to embryogenesis —a possibility in line with the lack of data on *ftz* expression during metamorphosis. Still, the identification of binding sites for transcription factors coded by genes which functions include involvement in metamorphosis supports the need for future analysis of *ftz* regulation and expression during metamorphosis. *ftz* is a segmentation and neurogenic gene which activation during the metamorphosis from the larvae to the adult fly state is logically expected.

As regulatory elements function by recruiting transcription factors, their transcription factor binding sites should, in principle, be functionally constrained, hence conserved. However, comparison between species shows TFBSs being continuously formed or lost. For instance, there is a newly formed EVEN-SKIPPED-binding site in the RCPE in *D. yakuba* and *D. teissieri*, a new TWIST-binding site in the RCPE of *D. orena* and *D. erecta*, and a new HUNCHBACK-binding site in the ZE of these last two species (Figure 4, Figure S1). In the ZE, but not in the RCPE, there is a significant excess (p<0.001) of losses over gains of TFBSs, which could represent a decay in the transcription factor-binding capacities of the sequences through the fixation of weakly deleterious changes.

In the RCPE, 348 of the 458 fixed base substitutions between species do not affect TFBSs, 68 cause their loss or gain, and 42 change one TFBS to another. Averaged across species, 88.09% of the RCPE bases are NTFBSs, the remaining 11.91% are TFBSs. If there was equal selective constraint in both types of sites, one would expect 404 ( = 458*0.8809) of the 458 base substitutions to occur in NTFBSs. A chi-squared test shows that the observed numbers differ significantly from the expected ones due to an excess of fixed substitutions in TFBSs (Chi-squared = 23.292, P<0.00001). Indeed, had TFBSs been at equilibrium such that rates of loss and gain were equal, 11.91% of substitutions would be expected to cause loss or change of site. 76 ( = (68/2)+42) is therefore the best estimate of the number of changes that, at equilibrium, occur in TFBSs, and 1.393 ( = 76/(458×0.1191)) is the best estimate of the proportion of mutations that spread to fixation in TFBSs relative to NTBFSs. This implies a 39.3% ( = (1.393−1)×100) increase in evolutionary rate in TFBSs compared to NTFBSs. Corresponding calculations for the ZE (55 out of the 84 fixed substitutions in NTFBSs, which represent 81.45% of the sequence, 22 making or destroying TFBSs, which are the remaining 18.55.% of the sequence, and 7 changing them) suggest that the evolutionary rate is increased by 15.52% in TFBSs relative to NTFBSs (Chi-squared = 5,130, P = 0,024). The observed increase in evolutionary rate, otherwise seen as indicative of evolutionary relaxation, was detected based on comparison of species-wide fixed substitutions (not polymorphic ones) and may, in this case, be interpreted as indicating greater selective constraint on TFBSs which may be evolving by fixation of adaptive substitutions. Nonetheless, the test for adaptive selection on TFBSs shows no significant differences in the ratio of fixed to polymorphic substitutions between TFBSs and NTFBSs in the sequences analyzed here, suggesting a lack of significant positive selection on TFBSs (Table 4).

**Table 4:** Testing for positive selection on *ftz* RCPE and ZE TFBSs.

Element	Method	Location and nature of the substitution	Fixed substitutions	Polymorphic substitutions	Two tailed Fisher's exact *P*
RCPE	PATCH™ public 1.0	TFBS	110	27	0.415
		NTFBS	348	106	
		Making new TFBS	27	11	0.460
		Destroying ancestral TFBS	41	11	
		Other TFBS changes	42	5	––
	MATCH™ public 1.0	TFBS	283 (123)	84 (31)	0.839 (0.435)
		NTFBS	175 (335)	49 (102)	
	DNase-I footprinting	TFBS	24	6	0.528
		NTFBS	57	16	
	Both	TFBS	300	89	0.836
		NTFBS	158	44	
	Phylogenetic footprinting	TFBS	195	61	1
		NTFBS	182	57	
ZE	PATCH™ public 1.0	TFBS	29	7	0.807
		NTFBS	55	17	
		Making new TFBS	3	1	1
		Destroying ancestral TFBS	19	5	
		Other TFBS changes	7	1	––
	MATCH™ public 1.0	TFBS	31 (13)	10 (4)	0.812 (1)
		NTFBS	53 (71)	14 (20)	
	DNase-I footprinting	TFBS	18	2	0.160
		NTFBS	20	7	
	Both	TFBS	47	14	1
		NTFBS	37	10	
	Phylogenetic footprinting	TFBS	45	8	0.288
		NTFBS	29	10	

The test for adaptive selection was carried out using the adaptation of McDonald and Kreitman test [18] suggested in [3], [20]. The numbers between parentheses refer to base substitutions affecting only the cores of TFBSs. In the case of DNase-I footprinting TFBSs and NTFBSs respectively refer to regions of the alignment corresponding or not to the DNase-I footprints reported for *D. melanogaster* in [29], [39], [40]. For the phylogenetic footprinting, however, TFBSs and NTFBSs respectively refer to regions of the alignment that are either well or badly aligned to *D. pseudoobscura* orthologs (see Materials and Methods).

Possible explanations for not detecting significant evidence for positive selection on TFBSs include uncertainties due to the treatment of each nucleotide of the TFBSs as equal in importance for transcription factor-binding, which would result in seemingly high evolutionary rate. One has also to consider uncertainties causing lower evolutionary rate of the NTFBSs; which could be constrained due to some unknown function, such as the presence of unknown or mismatched but functional TFBSs. While, from the results, false positives do not seem to be an issue for the stringent PATCH™ public 1.0 search, it is probable that the search criteria may have been excessively rigorous leading to false negatives noise. MATCH™ public 1.0 [41] was therefore used as another method to identify TFBSs, based on a minimum 100% core and 70% overall similarity of ≥5 bp regions of RCPE and ZE haplotypes to position weight matrices from the insect directory of the TRANSFAC® 6.0 database. This method allows for mismatches while differentiating between important positions of the TFBS (the core, where a strict 100% similarity cut-off was used) and the less conserved flanking sites. Again, the test for adaptive selection failed to detect significant signs of higher constraint neither on the cores nor on the extended TFBSs (Table 4). The result for TFBS cores is most surprising given that the core of a TFBS is identified based on nucleotide conservation across the different sequences empirically tested for binding to the transcription factor in question. Given that the position weight matrices method identifies a somewhat complementary set of TFBSs to that identified by the former method (see Figure 4, Figure S1), the combined set of TFBSs identified using both PATCH™ public 1.0 and MATCH™ public 1.0 searches was used to test for adaptive selection. Still, the test result was not significant (Table 4).

No significant difference in the ratio of polymorphisms to fixed changes in TFBSs compared NTFBSs was found even when functional allocation of sites was based on the results of DNase-I footprinting experiments reported in [29], [39], [40] (Table 4). Here, the part of the RCPE subjected to DNase-I footprinting is of 396 bases (averaged across species), 144 of which fell in footprints, and contained 81 base substitutions between species, of which 24 were in footprints (Table 4). The rate of evolution of bases in footprints relative to those not in footprints is therefore ∼26% lower. For the ZE, 128 bases out of the 361 analyzed fell in footprints. Since 18 of the 38 base differences between species are in footprints (Table 4), the rate of evolution is ∼64% higher in footprints than outside them. There was thus no evidence of significantly slower rates in footprints than outside them. The same is true for pooled data from both elements.

As a final test, identification of functionally important bases was inferred from phylogenetic footprinting after alignment of the sequences to their *D. pseudoobscura* orthologs. Since the phylogenetic footprinting method is independent of the other TFBSs detection methods, using it was a way to test whether the inability to detect adaptive selection was due to potential uncertainties in TFBSs identification. About half the interspecific base substitutions in the *D .melanogaster* subgroup are in regions well-aligned to *D. pseudoobscura* (195 in the PE, 45 in the ZE) and the other half in poorly-aligned regions (182 in the PE, 29 in the ZE). 65% of the RCPE bases are in well-aligned regions with a relative rate of base substitution evolution of 57.7% ( = (195×0.35)/(0.65×182)) —significantly different from 100%. The corresponding calculation in the ZE reveals a relative rate of 58.2% for well-aligned bases, also significantly different from 100%. Therefore, there is very significant evidence of stronger constraint in bases within the *D. melanogaster* subgroup that are well-aligned to *D. pseudoobscura* orthologs. Conservation in an outgroup thus offers a way of identifying functionally important bases. However, when the dichotomy of well- versus poorly-aligned sites is used to look for differences in fixed to polymorphism ratios for base substitutions, again no significant differences are seen (Table 4).

## Discussion

### ftz promoter and proximal enhancer regions are functionally constrained

Variation in *fushi tarazu*'s promoter and proximal enhancer regions was analyzed to detect functional constraints and to compare the relative strength of selection acting on different sequences and sequence regions. The results show that, just like *D. melanogaster* [33], [34], [35], [36], [37], *D. simulans* is less variable in Europe than in Africa probably due to a major bottleneck period in the evolutionary history associated with the colonization of Europe. *D. melanogaster* is the least variable species, probably due to the smaller effective population size evoked in [42] and references therein. The ZE shows more sequence conservation and seems to evolve less rapidly than the RCPE. If such difference is due to differences in function between a promoter and an enhancer it would reflect a possibly higher functional constraint on promoters than enhancers. Promoters are necessary for initiating gene expression whenever and wherever the gene in question is to be expressed. Enhancers, however, are responsible for the quantitative fine tuning and spatiotemporal differences in gene expression. Alteration of a promoter may result in potentially deleterious changes in gene regulation, whilst mutations in enhancers may have limited pleiotropy (*e.g.*, [43]) and may only cause less harmful quantitative or spatiotemporal variation in gene expression. However, both the RCPE and the ZE diverge at lower rate than that of the synonymous sites of *ftz* CDS. Haddrill and Charlesworth [44] and Haddrill *et al.* [45] reported non-neutral evolution of the non-coding genomic regions, and Andolfatto [19] reported significant conservation of many of these regions relative to synonymous sites. Since many of these regions correspond to promoters and enhancers, the higher conservation reported here for the RCPE and ZE seems a general characteristic of *cis*-regulatory DNAs, part of which may even show ultra-conserved modules (e.g.[46]).

Both the RCPE and ZE trees show similar distribution of the species. The variability of the RCPE (especially the inclusion of its highly variable part) and the close relatedness of the species analyzed here could explain the difference at the *D. melanogaster*-*D.simulans* group level, where the ZE tree is more in line with the predefined species phylogeny than the RCPE tree is. The differences at the fly line level could be attributed to differences between the RCPE and the ZE in haplotype numbers; reflective of the differences in sequence variability. However, some nodes between species branches and most of those between lines of the same species are supported at less than 60%, with the ZE tree showing the least overall bootstrap support. Such uncertainty could be attributed to the conservation of the sequences in question, which results in insufficient number of informative sites for the phylogeny to be robust. In line with the higher conservation of the ZE sequences, less haplotypes were found for this promoter and its phylogenetic tree shows less bootstrap support than that of the RCPE. It is also true that, although the phylogenetic relationship between some *Drosophila* species are clearer, a universally accepted *Drosophila* phylogeny is still not fully agreed upon (as examples see [47], [48], [49]).

In principle, a well supported phylogeny is important for the correct count of substitutions making new transcription factor sites and those destroying preexisting ones as, in case of multiple substitutions, chronology is key to determining the actual substitution that resulted in the making or destruction of the transcription factor binding site (see material and methods). However this is not an issue for this work as, the phylogenetic relationships I report at the species level are reasonably supported (as by the bootstraps) and concordant between the ZE and RCPE trees. Even in case of error, this will be a qualitative not quantitative (*i.e.*, there will be an issue of which among the multiple substitutions is the one responsible for making or destructing a TFBS, but only one substitution will be counted no matter how the phylogenetic relationships between the sequences are considered to be). Uncertainties within species (at the fly line level) would obviously have no effect on the results.

The ∼1.5 kb RCPE sequence is heterogeneous in its variability, with a highly variable 5′ region and a region with similar conservation to that of the ZE. This difference in sequence variability seems to reflect differences in functional constraints. Indeed, part of the variable RCPE region was used by Pick *et al.* [38] to transform *D. melanogaster* embryos but showed no sign of activity, whilst the less variable region contains the ∼400 bp minimal enhancer confirmed by DNase-I footprinting and germline transformation of *D. melanogaster* embryos by Han *et al.* [29], [30]. The uncharacterized ∼500 bp sequence between the proximal enhancer and the neurogenic one shows similar sequence variability to the promoter and minimal enhancer region, suggesting that it may be functionally constrained. Being within the *ftz cis*-regulatory sequence, the potential biological function of this region would be in *ftz* regulation, a possibility supported by the identification of multiple binding sites for developmental transcription factors within it. Confirmation and characterization of such a regulatory function could be achieved *via* germ-line transformation of *D. melanogaster* embryos.

### Lack of evidence for positive selection on the RCPE and ZE

Mutations in *cis*-regulatory elements are known to have phenotypic and evolutionary consequences [12]. Within a *cis*-regulatory element, one would expect TFBSs to be functionally constrained through purifying selection, whereas NTFBSs might, *a priori*, be expected to evolve neutrally. However, regulatory elements are a vital part of the on/off switching and fine-tuning of any gene network, so their sequence evolution may as well not obey such a simple dichotomy. On the one hand, TFBSs may have been selected for flexibility and/or redundancy, to insure robustness and evolvability of gene networks. On the other hand, NTFBSs could be constrained either in their length, as they may act in physically separating transcription factors, or in their base sequence, to allow chromatin bending for transcription factors to interact or to avoid the evolution of unwanted TFBSs in them.

Tests for adaptive evolution aim to disprove null hypotheses based on neutrality by looking for deviations that may be consistent with adaptive evolution. The paradigm from protein coding sequences is that bases that are functionally important (replacement sites) evolve more slowly and often show a higher ratio of fixed changes between species to polymorphisms than do the unconstrained synonymous bases. This logic was applied in [3], [20] to suggest a test based on Fisher's exact statistic. In this case, detection of adaptive selection on regions of the non-coding *cis*-regulatory elements uses the dichotomy between TFBSs (seen as equivalent to replacement sites) and NTFBSs (seen as equivalent to synonymous sites). One source of noise for this kind of tests is the increasing number of weakly deleterious changes that might be included in the polymorphisms as the sample size increases. For this reason, all polymorphisms in which the variant base was found in a single individual (save for those in *D. teissieri*) were removed from the calculations in the current data sets. In no instance did this alter the results.

The major obstacle to applying the logic of comparing the ratio of fixed to polymorphic substitutions between constrained and unconstrained parts of regulatory elements is to find appropriate rules for classification of bases into these two types. *ftz* promoter and proximal enhancer have already been experimentally tested for transcription factor binding in [29], [39], [40]. However, experimental identification of TFBSs poses great challenges, as the results depend on the material used and the experimental conditions. Good results depend on a representative and non-degraded protein sample. Furthermore, stringent conditions for transcription factors to bind to their sites would result in false negatives, whereas non-stringent conditions would cause false positives. One also has to consider that sites for transcription factors that are expressed at low levels or in a small set of cells will be hard to detect. All this makes the experimental approach less than definitive at detecting TFBSs. In an attempt to overcome the difficulties in TFBS identification, four different approaches were used here (see material and methods).

Each of these methods has its advantages and weaknesses. The sequence pattern search, matrix similarity, and the phylogenetic footprinting methods are symmetrical and unbiased. The experimental approach, however, is asymmetrical with respect to the species and might become increasingly noisy, although not biased, especially if species too diverged from *D. melanogaster* are included in the comparison. Nevertheless, this is not a major concern for this work as it uses very closely related species. Since the search was restricted to ≥5 pb perfect matches to experimentally tested sequences of insect TFBSs, the sequence pattern search is less likely to yield false positives. However, it could miss true (*i.e.*, functional) but diverged (*i.e.*, mismatched) TFBSs. These would be identified by the matrix similarity method, though this method relies on the use of adequate similarity cut-offs. In addition, both computational methods suffer from the limitations of the database —databases contain only some of the sequences (*i.e*, forms) of the binding sites of only some transcription factors and only mostly from model species (*D. melanogaster* in this case). However, overall, the computational searches seem neither too restrictive nor too permissive, as they detected binding sites for transcription factors either known to regulate *ftz* or could be doing so given what we know about *Drosophila* developmental gene networks (see Figure 4, Table S3 and Figure S1). They also identified the TFBSs confirmed by DNase-I footprinting in [29], [39], [40]. Interestingly, potentially genuine binding sites for transcription factors hitherto not reported to regulate *ftz* were identified here, thus highlighting that the story of *ftz* expression may still be to complete; especially during metamorphosis. Indeed, *ftz* is both a segmentation and a neurogenic gene which reactivation during metamorphosis could be expected given that, from a larvae to an adult fly, the segments are rearranged and the neural system architecture is changed.

The empirical approach should, in principle be the most reliable. Nonetheless, it also has its uncertainties, as the result depends on the experimental conditions (temperatures, salts concentration…) as well as the quality and representativeness of the protein mix used to footprint the regulatory element. Binding sites for transcription factors expressed at low levels or in developmental stages or tissues underrepresented or not included in the experiment will be missed. Similarly, TFBSs will be missed in stringent experimental conditions (low protein quantities, high temperatures and/or salts concentrations…). In less stringent experimental conditions, however, sites for non-specific DNA-binding proteins, many of them with no involvement in the correct functioning of the regulatory element analysed, will also be footprinted. In addition, in some cases, not every single nucleotide protected from DNase-I digestion actually belongs to the genuine TFBS.

The phylogenetic footprinting, however, depends neither on experimental conditions nor on databases. Its applicability only depends on choosing a neither-so-distant-nor-too-close ‘outgroup’ species and correctly aligning and identifying the footprints. Functionally unconstrained regions may still perfectly align if the outgroup is phylogenetically too close to the sequences being analysed (*i.e.*, the divergence time is not enough for mutations to accumulate in functionally unconstrained regions). However, if the outgoup is too divergent, even functionally constrained regions may have had enough time to accumulate adaptive substitutions and not align to the orthologous regions in the sequences being analysed. In this case, *D. pseudoobscura* is an obviously excellent reference since it is not too close nor too diverged from the species analyzed here (∼25 million years divergence time [50]) and we can easily identify significant stretches of perfectly aligned sites separated by stretches of poorly aligned ones. In addition, a minimum of six base pairs five of which perfectly matching a *D. melanogaster* subgroup ortholog is a realistic estimation for a phylogenetic footprint.

Surprisingly, the test for adaptive selection consistently fails to detect signs of adaptive evolution no matter what method is used to identify the TFBSs (note that both the experimental and the phylogenetic footprinting approaches for TFBS identification are independent from each other and from the two computer-based methods). A high evolution rate of TFBSs could have many causes. Some changes in TFBSs might have little effect on the binding capacity, which would confer flexibility and insure functional robustness of the transcription factor and its binding site. Similarly, functional redundancy could allow changes to accumulate in TFBSs, thus allowing functional robustness of the regulatory element as well as some evolvability. Indeed, TFBSs are known to consist of a consensus of a conserved core flanked by less conserved positions, and almost all the TFBSs occur more than once in the RCPE and ZE (Figure 4, Figure S1). Such finding is in agreement with Hancock *et al.* ´s description of eukaryotic promoters as consisting of “modular and redundant elements that are bound by a number of *trans*-acting regulatory proteins and have been shown to vary in copy number, sequence, interelement spacing, binding affinity, and orientation within and between species in some well-studied cases” [51]. Another explanation could be compensatory mutations. In bottlenecks of low population size, weakly deleterious changes/losses in TFBSs might subsequently be compensated for by selectively-driven gains of advantageous mutations/sites elsewhere in the sequence. Such compensatory changes might be detected by correlations between mutations removing and forming binding sites in a given branch of the phylogenetic tree. The evolutionary changes in the TFBSs could therefore be potentially adaptive, some of which may be compensatory. Indeed, TFBSs can evolve by adaptive mutations [52] that result from the co-evolution of the TFBS and the DNA-binding specificity of its transcription factor. Such co-evolution is probably a contributor to what Andolfatto [19] reported as evolutionary constraints causing sub-estimation of functional relevance. Furthermore, compensatory changes have been suggested for elements such as the stripe 2 enhancer of the pair-rule gene *even-skipped* [2], [53], the enhancer of *tailless* [54] and, among the various other examples of previous works reporting compensatory mutations on *cis*-regulatory elements, one can also cite [54], [55].

Undoubtedly both *cis*-regulatory elements analyzed here show higher conservation levels than the synonymous sites of the *ftz* CDS. There are many potential sources of constraint on regions not known to bind transcription factors; (i) their sequence composition may be important for DNA looping, (ii) they may be under selection to prevent the evolution of TFBSs within them, (iii) they may contain binding sites for structurally important proteins or (iv) for unknown transcription factors, or (v) they may contain mismatched but still functional TFBSs. Certainly, *cis*-regulatory function doesńt depend only on a simple distribution of TFBSs (*e.g.*, [51], [56], [57], [58], [59], [60], [61]). So, among others, *cis*-regulatory function also depends on the number of TFBSs, their positioning, spacing, interactions, as well as the general structure of the element (*e.g.*, its position and looping potential) which also depend on the size, positions and sequences of the NTFBSs. Still, DNA looping shouldn't require strict conservation of sequences (even palindrome can tolerate mismatches) and, probabilistically, most substitutions are more likely to take a sequence away from a ‘target’ sequence (TFBS) than to drive it closer. I therefore think that the conservation of the relatively large DNA stretches identified as NTFBSs is likely due to them containing binding sites for structurally important proteins, or unknown or mismatched but functional TFBSs. This possibility is highly supported by the conservation of some regions identified as NTFBSs between *D. melanogaster* subgroup and the more distantly related *D. pseudoobscura* and *D. virilis* (data not shown). In addition, with so many TBFSs being identified in the sequences, my calculations reveal that, had I allowed for mismatches in the PATCH public 1.0 search, almost all of the DNAs would be identified as TFBSs, most of which are obviously spurious (data not shown).

The current work highlights the high complexity of *cis*-regulatory DNAs and reflects the yet not totally deciphered complexity and spatiotemporal dynamism of gene expression and its *cis*-control which, ultimately, result in the fine tuning of tremendously complex and dynamic gene networks and phenotypes. Indeed, combining the TFBSs search methods, about two thirds of the sites of the sequences analyzed seem to be TFBS; a figure strikingly similar to the ratio of non-synonymous to synonymous sites in coding sequences (*e.g.*, 69% of the *D. melanogaster* ZE and 67% of its RCPE sites are potential TFBSs). With lower nucleotide variability than the neutrally evolving synonymous sites of *ftz* CDS, *cis*-regulatory elements are clearly functionally constrained and not evolving neutrally. An expected result given their function as on/off switches and fine tuners of the spatiotemporal expression of the genes —a function of obvious and vital importance for the survival, adaptation and evolution of species. Yet *cis*-regulatory elements also show signs of flexibility and functional redundancy; which should insure functional robustness and potential for evolvability of the spatiotemporal expression of the genes and the networks they are part of.

The non-detection of significant signs of adaptive selection of the *cis*-regulatory elements analyzed in this work does not mean that these are evolving neutrally. Indeed, the results of this work may even be yet another example of works supporting the idea that DNA sequence evolution is not necessarily governed by a simple, nor easy to detect, neutrality/selection dichotomy. Other processes, including biased gene conversion [62], [63], are to consider. Indeed, non-adaptive processes such as genetic drift, mutation and recombination are suggested as forces acting on the evolution of many quantitative aspects of known transcriptional networks (see [64]). In my opinion, *cis*-regulatory elements, including their TFBSs, are among the best materials for analyzing evolution by molecular drive (for more about the molecular drive hypothesis see [65], [66], [67]) —a process which I previously suggested as the driving force for the evolution of other protein binding sequences [68].

The current work also highlights the need for further analysis of the functional constraints acting on non-coding DNAs as well as more work on structure and function of *cis*-regulatory regions and better characterization of transcription factor-binding sequences and consensuses. As to *ftz* interactions, they seem to be more complex than what we currently know with a probable activity of this gene during metamorphosis. Although none of the identified factors is a strictly metamorphosis one so, in spite of their involvement in metamorphosis, their action on *ftz* expression could be restricted to the embryogenesis period. The finding of binding sites for several transcription factors which functions include involvement in ecdysis and metamorphosis supports *ftz* activation during metamorphosis. Testing such activity should be possible in a number of ways including *in situ* hybridization with labeled *ftz* antisense probes or immunohistochemical staining with an anti-FTZ antibody.

## Materials and Methods

I analyzed the sequence containing *ftz*'s proximal enhancer and ∼0.5 kb of the uncharacterized DNA separating it from *ftz*'s neurogenic enhancer (RCPE) (positions −4964 to −3437 relative to *ftz* start codon in *D. melanogaster*), and the ∼0.8 kb *ftz*'s zebra element (ZE) (positions −794 to +82 relative to start codon) (Figure 1). I used 45 lines from 20 populations of eight *Drosophila* species —most of them kind gifts from Dr. Peter Andolfatto, Dr. Penelope Haddrill, Dr. Harmit Malik and Dr. Rhonda Snook (see Table S1 and Acknowledgments).

### Single fly DNA extraction

Genomic DNA was extracted from single adult flies using a modified version of the method described in [69]. Single adult flies were homogenized in 50 µl of a simplified homogenization buffer containing: 10 mM Tris-HCl [pH 7.5], 60 mM NaCl, 10 mM EDTA, 1 g sucrose, and H_2_O to a final volume of 20 ml. 50 µl of lysis buffer (300 mM Tris-HCl [pH 9], 100 mM EDTA, 0.625% Sodium Dodecyl Sulfate, 1 g sucrose, H_2_O to 20 ml) was then added before mixing and incubating for 15 min at 70°C. Once the lysate cooled to room temperature, 15 µl of 8 M Potassium Acetate was added, and the mixture incubated in ice for 30 minutes. After centrifugation for one minute at 1000 rpm, the supernatant was extracted with an equal volume (115 µl) of phenol/chloroform (1∶1) and centrifuged for three minutes at 5000 rpm. The supernatant was then removed to a new tube and 115 µl of TE, pH 7.6 (10 mM Tris-HCl, pH 7.6 and 1 mM EDTA) added to the remaining organic phase before mixing, centrifuging at 5000 rpm for three minutes, adding the supernatant to the previous one before going through another round of phenol/chloroform extraction. After extraction in an equal volume of chloroform, 23 µl of 3 M Sodium Acetate [pH 4.6] and 575 µl of 95% ethanol were added to the 230 µl of supernatant for DNA precipitation for 30 minutes at 13000 rpm. The DNA pellet was washed with 400 µl 70% ethanol and, once centrifuged at 13000 rpm for 10 minutes and air dried, dissolved in 50 µl of distilled water.

### Polymerase Chain Reactions

Reactions were carried out in 600 µl eppendorf tubes using a Perkin Elmer Cetus DNA Thermal Cycler, and contained: Genomic DNA (3 µl), forward and reverse primers at 100 µM (0.25 µl) each, 4 µl of an equal volume mix of the four dNTPs at 1.25 mM each, 2.5 µl 10×PCR buffer, 25 mM MgCl_2_ (1.25 µl), 0.25 µl of 5 U/µl Taq DNA polymerase, 13.5 µl H_2_O and a drop of DNase-free mineral oil (Sigma). The primers used and their melting temperatures can be found in Table S2. All PCR cycles were as follows: 5 minutes at 94°C, (30 seconds at 94°C, 30 seconds at 60°C, 1 minute at 72°C)×5, (30 seconds at 94°C, 30 seconds at 55°C, 1 minute at 72°C)×5, (30 seconds at 94°C, 30 seconds at 50°C, 1 minute at 72°C)×20, and 5 minutes at 72°C.

### Agarose gel electrophoresis and DNA extraction

After electrophoresis in 1% low-melting temperature agarose in 0.5×TBE (Tris 5.4 g, Boric Acid 2.75 g, EDTA 0.465 g and H_2_O to 1 liter), slices were cut from the gel and the DNA extracted using a modified version of the freeze and thaw technique described in [70]. Each gel slice was frozen in a 1.5 ml eppendorf tube then, after it came to room temperature, centrifuged for 5 minutes at 13000 rpm, and the liquid phase saved. 500 µl TE (pH 7.6 —see above) was added to the remaining solid phase for melting. Once melted, the content of the tube was mixed, and frozen in an inverted position. Once defrost, the tube was centrifuged at 13000 rpm for 5 minutes. The pooled liquid phases were ethanol precipitated as described above.

### DNA sequencing

40 cycles sequencing reactions were carried out in 10 µl reaction volumes containing: DNA (0.5 µl), primer at 100 µM (0.25 µl) (Table S2), Applied Biosystems Big Dye terminator 3.1 mix (2 µl), 5×Applied Biosystems Big Dye terminator 3.1 sequencing buffer (2 µl), H_2_O (5.25 µl) and a drop of DNase-free mineral oil (Sigma). The cycles were 96°C for 1 minute, (96°C for 10 seconds, 50°C for 5 seconds, 60°C for 4 minutes)×40, 4°C until DNA precipitation. Ethanol precipitation was carried out for 30 to 45 minutes on ice using 26 µl of the 95% ethanol-sodium acetate mixture described above. This was followed by centrifugation at 13000 rpm for 30 minutes, washing with 250 µl 70% ethanol and centrifugation for 10 minutes at 13000 rpm. The DNA was then dried at 90°C for 1 to 3 minutes. Sequence reading was carried out in an Applied Biosystems 3730xl DNA Analyzer of the Department of Biochemistry, University of Oxford. Both strands of each DNA fragment were sequenced, and most fragments were sequenced more than once.

### Sequence analyses

Sequences were edited using the sequence alignment editor BioEdit 7.0.1 [71] and aligned using CLUSTAL W [72]. For accuracy, sequence alignments were manually edited and the result compared to that obtained using DiAlign version 2.2.1 [73]. PHASE 2.1 [74], [75] was used for estimating haplotypes from genotype data, and phylogenetic trees were constructed using the maximum likelihood method in the program PHYLIP [76] and the sequence of *D. pseudoobasura* as outgroup (accession number AY190944).

The program DnaSP 4.0 [77] was used to perform analyses of the intra- and inter-specific variability of the sequences. A modified version of the equation suggested by Schneider and Stephens [78] for constructing sequence logos was used to quantify conservation along the alignment of the sequences. For each nucleotide at each position, excluding gaps, a conservation index was calculated as *(n/s)(log_2_(1/s)-log_2_(n/s))/log_2_(1/s)*, where *n* is the number of sequences bearing that nucleotide at that position, and *s* is the total number of sequences in the alignment. The maximum conservation index is 1, and 0 was attributed to unrepresented nucleotides. The sum of the conservation indexes of the nucleotides at each position of the alignment reflects the conservation at that particular position.

Searching for TBSs *in silico* depends both on the database of transcription factor binding sequences and on the algorithm used for searching that database. Here, I relayed on the TRANSFAC^®^ 6.0 database —which is the best and most extensive available database of experimentally tested transcription factor-binding sequences [79], [80], [81]. Initially, the program PATCH™ public 1.0 (Jochen Striepe and Ellen Goessling, Biobase, GmbH) was used to identify the TFBSs in each RCPE and ZE haplotype by alignment to actual sequences in the TRANSFAC® 6.0 database. To avoid false positives, the search was limited to transcription factor binding sequences belonging to insects, sequences had to be at least 5 bp in length and no mismatch was allowed (*i.e*., perfect alignments). To further avoid false positives, an extensive literature search was performed and the authenticity of the TFBSs identified was evaluated based on our knowledge of *ftz* expression and interactions (see Table S3).

Fixed and polymorphic substitutions, affecting TFBSs and not affecting TFBSs, were counted. The rule was that a base change is classified as affecting a TFBS if it affects a sequence of bases identified as TFBS either before or after that change happened. Based on the phylogeny of the analyzed species, only the chronologically first substitution was counted in case of multiple substitutions eliminating a pre-existing TFBS, and only the last one was counted in case of multiple substitutions forming new TFBSs. This was aimed at reducing the noise that can originate from redundancies and misplacements when allocating substitutions to TFBSs and NTFBSs. Testing for positive selection acting on TFBSs was carried out using the adaptation of McDonald and Kreitman test [18] suggested in [3], [20].

Given that no significant sign of adaptive selection was detected on the TFBSs identified using PATCH™ public 1.0. I decided to complement and test the results using other methods of TFBSs identification. So, to take into account that transcription factors usually do not bind just one type of sequence and that the PATCH method will inevitably result in some false negatives, I added a computational method that relays on position weight matrices and, thus, takes into account the ‘degeneracy’ of the TFBSs. For this, I used the program MATCH™ public 1.0 (Alexander Kel and Ellen Goessling, Biobase GmbH [41]) to identify the TFBSs in the RCPE and ZE haplotypes this time as ≥5 bp sequences having 100% core and 70% overall similarity to position weight matrices (PMWs) of insect transcription factor binding sequences from the TRANSFAC® 6.0 database. As with the PATCH™ public 1.0 results, the authenticity of the TFBSs identified using MATCH™ public 1.0 was also evaluated based on extensive literature searches and our knowledge of *ftz* expression and interactions (see Table S3).

Another way of identifying TFBSs is by experimentally testing their binding capacity using DNase-I footprinting experiments (*e.g.*, [82], [83]). Luckily, this has already been done in *D. melanogaster* for regions of both the RCPE and ZE [29], [39], [40]. These experimental data were therefore used to determine the fixed and polymorphic substitutions that affect regions of the haplotypes corresponding to the *D. melanogaster* footprints (TFBSs) and those that affect regions not footprinted (NTFBSs).

A final approach identified functionally important bases through conservation in more distant comparisons —a method referred to as phylogenetic footprinting (*e.g.*, [84], [85], [86], [87]). Alignment of the sequences with *D. pseudoobscura* othologs is possible, although it is expectedly uncertain in some regions. A statistic was thus developed to separates sequence parts into regions well-aligned, defined as any stretch of at least six *D. pseudoobscura* bases at least five of which match to at least one of the *D. melanogaster* subgroup orthologs, and regions badly-aligned.

## Supporting Information

Figure S1 **Haplotypes and TFBSs of the RCPE (A) and ZE (B) from the eight** ***Drosophila*** **species studied in this work.** Shaded positions are those indentified by PATCH searches as 5 bp minimum sequences that are perfect matches to transcription factor binding sequences at the insect directory of the TRANSFAC database. Boxes delimit TFBSs identified by MATCH searches as having 100% similarity at the core and at least 70% overall similarity to position weight matrices at the insect directory of the TRANSFAC database. h1, h2…: Haplotype 1, 2…. me: *D. melanogaster*, si: *D. simulans*, se: *D. sechellia*, ma: *D. mauritiana*, ya: *D. yakuba*, te: *D. teissieri*, or: *D. orena*, er: *D. erecta*. Underlined: Nucleotides shared between two different transcription factor-binding regions identified by PATCH. Double underlined: nucleotides shared between three different transcription factor-binding regions identified by PATCH. Strikethrough: Nucleotides at the core sequence of a TFBS identified by MATCH. DNase-I footprinting data are those in references [29], [39], [40] of the manuscript. S: Start of the DNase-I footprinted sequence. E: End of the DNase-I footprinted sequence. P: Transcription factor binding position. The arrow marks the *ftz*'s transcription start. ^1^: Here it is assumed that the ancestor was polymorphic for the nucleotides at this position some of which were subsequently fixed in some clades/branches. ^2^: It is assumed that a nucleotide that is polymorphic in *D. simulans* or in *D. yakuba* —of which more than one line were analyzed— is very likely also polymorphic in their respective geographical daughter/sister species *D. sechellia*, *D. mauritiana* or in *D. teissieri* —of which only a single line was analyzed. The aim of rules ^1^ and ^2^ is to avoid noise caused by false positives in fixed substitutions. Sequences in GenBank accession numbers HQ693575- HQ693658.
(DOC)

Table S1 **Fly species, populations and lines, their origin and the colleagues who kindly offered them.**
(DOC)

Table S2 **Primers used for PCR amplification and cycle sequencing of the RCPE and ZE DNAs.** Selection of the primers was based on conservation between the aligned sequences of *D. melanogaster* and *D. pseudoobscura* available at GenBank (accession numbers AE003673 and AY190944). *: Both *D. orena* and *D. erecta* have a 9 bp deletion within the RCPE reverse primer, underlined bases, so RCPE reverse2 was used as alternative reverse primer for amplifying the RCPE from these two species.
(DOC)

Table S3 **Transcription factor binding sites identified in haplotypes of the** ***Drosophila*** **region containing** ***ftz*** **proximal enhancer (RCPE) and zebra element (ZE) and estimation of the likelihood of** ***ftz*** **regulation by their binding proteins based on our knowledge on gene expression and function.** The search was performed using the insect directory of the TRANSFAC® 6.0 database [99,100,101,102,103] with the help of the programs PATCH™ public 1.0 (limited to perfect matches of at least 5 bases) and MATCH™ public 1.0 [104] (using 70% as a minimum overall similarity and 100% as core similarity cut-offs). *ftz* is a segmentation gene with neurogenic involvement it is known to be active late in development [105] but not studied during metamorphosis; although it wouldńt be unwise to expect it to be expressed throughout that period, especially since it is activated by the nuclear hormone receptor FTZ-F1 (see above). It has multiple positive and negative regulators that shape the variation of its spacio-temporal expression. When a gene is not known to regulate *ftz* it was assessed based on its spacio-temporal expression, its position downstream of *ftz* and the possibility of a feedback, its involvement in segmentation, neurogenesis or molting/metamorphosis. It is also worth mentioning that, while the zebra element and the proximal enhancer-containing sequences analyzed in this work are of ∼800 bp and 1500 bp respectively, the experimental testing of transcription factor binding to these elements has been performed only for parts of these sequences (about 400 bp (see [22,98,106])).
(DOC)
