# Supplementary material for: Microevolution of cis-Regulatory Elements: An Example from the Pair-Rule Segmentation Gene fushi tarazu in the Drosophila melanogaster Subgroup
Source: PLoS One. 2011 Nov 3;6(11):e27376. doi: 10.1371/journal.pone.0027376 (PMC3207857; doi:10.1371/journal.pone.0027376)
Supplement: Figure S1 — Haplotypes and TFBSs of the RCPE (A) and ZE (B) from the eight Drosophila species studied in this work. Shaded positions are those indentified by PATCH searches as 5 bp minimum sequences that are perfect matches to transcription factor binding sequences at the insect directory of the TRANSFAC database. Boxes delimit TFBSs identified by MATCH searches as having 100% similarity at the core and at least 70% overall similarity to position weight matrices at the insect directory of the TRANSFAC database. h1, h2…: Haplotype 1, 2…. me: D. melanogaster, si: D. simulans, se: D. sechellia, ma: D. mauritiana, ya: D. yakuba, te: D. teissieri, or: D. orena, er: D. erecta. Underlined: Nucleotides shared between two different transcription factor-binding regions identified by PATCH. Double underlined: nucleotides shared between three different transcription factor-binding regions identified by PATCH. Strikethrough: Nucleotides at the core sequence of a TFBS identified by MATCH. DNase-I footprinting data are those in references [29], [39], [40] of the manuscript. S: Start of the DNase-I footprinted sequence. E: End of the DNase-I footprinted sequence. P: Transcription factor binding position. The arrow marks the ftz's transcription start. 1: Here it is assumed that the ancestor was polymorphic for the nucleotides at this position some of which were subsequently fixed in some clades/branches. 2: It is assumed that a nucleotide that is polymorphic in D. simulans or in D. yakuba —of which more than one line were analyzed— is very likely also polymorphic in their respective geographical daughter/sister species D. sechellia, D. mauritiana or in D. teissieri —of which only a single line was analyzed. The aim of rules 1 and 2 is to avoid noise caused by false positives in fixed substitutions. Sequences in GenBank accession numbers HQ693575- HQ693658. (DOC) [file pone.0027376.s001.doc]

10 20 30 40 50 60 70 80 90 100

....|....|....|....|....|....|....|....|....|....|....|....|....|....|....|....|....|....|....|....|

**RCPE me h1**  **CGTACGACTCGTCA~~GCGGAA~~TCGCATTGACAGCCTACGCCGAACTCCCCGGAGACCT~~CCGTTCCG~~ATCATTG~~TTAATG~~GAC~~GTTAT~~CCTTATTAG~~ATGTT~~**

**RCPE me h2**  **....................................................................................................**

**RCPE me h3**  **.......................................A............................................................**

**RCPE me h4**  **....................................................................................................**

**RCPE me h5**  **.......................................A............................................................**

**RCPE me h6**  **....................................A..A............................................................**

**RCPE me h7**  **....................................................................................................**

**RCPE me h8**  **....................................................................................................**

**RCPE me h9**  **....................................................................................................**

**RCPE me h10**  **....................................................................................................**

**RCPE me h11**  **....................................................................................................**

**RCPE me h12**  **....................................................................................................**

**RCPE me h13**  **....................................................................................................**

**RCPE me h14**  **....................................................................................................**

**RCPE me h15**  **....................................................................................................**

**RCPE me h16**  **....................................................................................................**

**RCPE me h17**  **....................................................................................................**

**RCPE me h18**  **....................................................................................................**

**RCPE me h19**  **....................................................................................................**

**RCPE me h20**  **....................................A..A............................................................**

**RCPE si h1**  **......................................T........................................................C....**

**RCPE si h2**  **......................................T........................................................C....**

**RCPE si h3**  **......................................T........................................................C....**

**RCPE si h4**  **......................................T........................................................C....**

**RCPE si h5**  **......................................T........................................................C....**

**RCPE si h6**  **......................................T........................................................C....**

**RCPE si h7**  **......................................T........................................................C....**

**RCPE si h8**  **......................................T........................................................C....**

**RCPE si h9**  **......................................T........................................................C....**

**RCPE si h10**  **......................................T........................................................C....**

**RCPE si h11**  **......................................T........................................................C....**

**RCPE si h12**  **......................................T........................................................C....**

**RCPE si h13**  **......................................T........................................................C....**

**RCPE se h1**  **......................................T......T.................................................C....**

**RCPE ma h1**  **......................................T...................T....................................C....**

**RCPE ya h1**  **............T..................................................................C.T..G..........C....**

**RCPE ya h2**  **............T..................................................................C.T..G..........C....**

**RCPE ya h3**  **............T..................................................................C.T..G..........C....**

**RCPE ya h4**  **............T..................................................................C.T..G..........C....**

**RCPE ya h5**  **............T..................................................................C.T..G..........C....**

**RCPE ya h6**  **............T..................................................................C.T..G..........C....**

**RCPE ya h7**  **............T..................................................................C.T..G..........C....**

**RCPE ya h8**  **............T..................................................................C.T..G..........C....**

**RCPE ya h9**  **............T..................................................................C.T..G..........C....**

**RCPE ya h10**  **............T..................................................................C.T..G..........C....**

**RCPE ya h11**  **............T..................................................................C.T..G..........C....**

**RCPE ya h12**  **............T..................................................................C.T..G..........C....**

**RCPE ya h13**  **............T..................................................................C.T..G..........C....**

**RCPE ya h14**  **............T..................................................................C.T..G..........C....**

**RCPE ya h15**  **............T..................................................................C.T..G..........C....**

**RCPE ya h16**  **............T..................................................................C.T..G..........C....**

**RCPE te h1**  **............T..................................................................C.T..G..........C....**

**RCPE te h2**  **............T..................................................................C.T..G..........C....**

**RCPE or h1**  **..........................G...........T......................C.T...............C.T..G..........CC...**

**RCPE er h1**  **......................................T........................T...............C.T..G..........C....**

**Fixed substitutions**  **____________1_____________1___________1^1^_____1____________1__1_1_______________1_1__1__________11___**

**Polymorphic substitutions** **____________________________________1__1____________________________________________________________**

**DNAse footprinting data ____________________________________________________________________________________________________**

110 120 130 140 150 160 170 180 190 200

....|....|....|....|....|....|....|....|....|....|....|....|....|....|....|....|....|....|....|....|

**RCPE me h1**  **GATGT~~CCCAC~~GATGTC------------G~~AA-TA~~----------------------------~~G~~C~~TACGG~~GACATACATATCTA~~CATATAT~~----------**

**RCPE me h2**  **..............C.------------...-..----------------------------............................----------**

**RCPE me h3**  **................------------...-..----------------------------.........................C..----------**

**RCPE me h4**  **................------------...-..----------------------------............................----------**

**RCPE me h5**  **..............C.------------...-..----------------------------.........................C..----------**

**RCPE me h6**  **...............T------------...-..----------------------------............................----------**

**RCPE me h7**  **...............T------------...-..----------------------------............................----------**

**RCPE me h8**  **...............T------------...-..----------------------------............................----------**

**RCPE me h9**  **................------------...-..----------------------------............................----------**

**RCPE me h10**  **................------------...-..----------------------------............................----------**

**RCPE me h11**  **..............C.------------...-..----------------------------.........................C..----------**

**RCPE me h12**  **..............C.------------...-..----------------------------.........................C..----------**

**RCPE me h13**  **................------------...-..----------------------------............................----------**

**RCPE me h14**  **..............C.------------...-..----------------------------............................----------**

**RCPE me h15**  **................------------...-..----------------------------............................----------**

**RCPE me h16**  **................------------...-..----------------------------............................----------**

**RCPE me h17**  **................------------...-..----------------------------............................----------**

**RCPE me h18**  **................------------...-..----------------------------.........................CG.----------**

**RCPE me h19**  **................------------...-..----------------------------.........................CG.----------**

**RCPE me h20**  **...............T------------...-..----------------------------............................----------**

**RCPE si h1**  **................------------...-..----------------------------.............----...--------GCCCT---GC**

**RCPE si h2**  **................------------...-..----------------------------.............----...--------GCCCT---GC**

**RCPE si h3**  **................------------...-..----------------------------.............----...--------GCCCT---GC**

**RCPE si h4**  **................------------...-..----------------------------.............----...--------GCTCT---GC**

**RCPE si h5**  **................------------...-..----------------------------.............----...--------GCCCT---GC**

**RCPE si h6**  **................------------...-..----------------------------.............----...--------GCCCT---GC**

**RCPE si h7**  **................------------...-..----------------------------.............----...--------GCCCT---GC**

**RCPE si h8**  **................------------...-..----------------------------.............----...--------GCTCT---GC**

**RCPE si h9**  **................------------...-..----------------------------.............----...--------GCCCT---GC**

**RCPE si h10**  **................------------...-..----------------------------.............----...--------GCTCT---GC**

**RCPE si h11**  **................------------...-..----------------------------.............----...--------GCCCT---GC**

**RCPE si h12**  **................------------...-..----------------------------.............----...--------GCCCT---GC**

**RCPE si h13**  **................------------...-..----------------------------.............----...--------GCCCT---GC**

**RCPE se h1**  **................------------...-..----------------------------.............----...--------GCCCT---GC**

**RCPE ma h1**  **................------------...-..----------------------------.............----...--------GCCCT---GC**

**RCPE ya h1**  **................------GATGTC...-..TG----TCCGTGTCGGCAAA-------------------------...--------GCCGT---GC**

**RCPE ya h2**  **................------GATGTC...-..TG----TCCGTGTCGGCAAA-------------------------...--------GCCGT---GC**

**RCPE ya h3**  **................GATGTTGATGTC...-..TG----TCCGTGTCGGCAAA-------------------------...--------GCCGT---GC**

**RCPE ya h4**  **................GATGTCGATGTC...-..TG----TCCGTTTCGGCAAA-------------------------...--------GCCGT---GC**

**RCPE ya h5**  **................------GATGTC...-..TG----TCCGTGTCGGCAAA-------------------------...--------GCCGT---GC**

**RCPE ya h6**  **................------GATGTC...-..TG----TCCGTGTCGGCAAA-------------------------...--------GCCGT---GC**

**RCPE ya h7**  **................GATGTCGATGTC...-..TG----TCCGTGTCGGCAAA-------------------------...--------GCCGT---GC**

**RCPE ya h8**  **................GATGTTGATGTC...-..TG----TCCGTGTCGGCAAA-------------------------...--------GCCGT---GC**

**RCPE ya h9**  **................GATGTTGATGTC...-..TG----TCCGTTTCGGCAAA-------------------------...--------GCCGT---GC**

**RCPE ya h10**  **................GATGTCGATGTC...-..TG----TCCGTGTCGGCAAA-------------------------...--------GCCGT---GC**

**RCPE ya h11**  **................------GATGTC...-..TG----TCCGTGTCGGCAAA-------------------------...--------GCCGT---GC**

**RCPE ya h12**  **................------GATGTC...-..TG----TCCGTGTCGGCAAA-------------------------...--------GCCGT---GC**

**RCPE ya h13**  **................GATGTCGATGTC...-..TG----TCCGTGTCGGCAAA-------------------------...--------GCCGT---GC**

**RCPE ya h14**  **................GATGTCGATGTC...-..TG----TCCGTGTCGGCAAA-------------------------...--------GCCGT---GC**

**RCPE ya h15**  **................GATGTCGATGTC...-..TG----TCCGTGTCGGCAAA-------------------------...--------GCCGT---GC**

**RCPE ya h16**  **................GATGTCGATGTC...-..TG----TCCGT~~TGCGGGAAA~~-------------------------...--------GCCGT---GC**

**RCPE te h1**  **...............-------------...C..TGTATGTCTGTGTCGGCAAA-------------------------...--------GTCTT---GC**

**RCPE te h2**  **...............-------------...C..TGTATGTCTGTGTCGGCAAA-------------------------...--------GTCTT---GC**

**RCPE or h1**  **................------------...C-.T-------------------GGGACTTGT..........CG----...--------GCCAA---GC**

**RCPE er h1**  **..............C.------------...-----------------------------~~TA~~...G.........----...--------GCCAACAAGC**

**Fixed substitutions**  **______________11­______1________21_111_____1___________1_____111_­_1_______111______1_______11_311____**

**Polymorphic substitutions** **______________111____1_______________________11___1____________________________________11___1_______**

**DNAse footprinting data ____________________________________________________________________________________________________**

210 220 230 240 250 260 270 280 290 300

....|....|....|....|....|....|....|....|....|....|....|....|....|....|....|....|....|....|....|....|

**RCPE me h1**  **-----~~AAAAGATAT~~G-----------CCCATGT~~TAGGTATTAA~~GATCTGATTCAGTTCA--TGTTCAGCTT~~CATTATATTA~~--T~~AGATTAAA~~ATTATGT~~G~~**

**RCPE me h2**  **-----..........-----------.................................--....................--.................**

**RCPE me h3**  **-----..........-----------.................................--....................--.................**

**RCPE me h4**  **-----..........-----------.................................--....................--.................**

**RCPE me h5**  **-----..........-----------.................................--....................--.................**

**RCPE me h6**  **-----..........-----------.................................--....................--.................**

**RCPE me h7**  **-----..........-----------.................................--....................--.................**

**RCPE me h8**  **-----..........-----------.................................--....................--.................**

**RCPE me h9**  **-----..........-----------.................................--....................--.................**

**RCPE me h10**  **-----..........-----------.................................--....................--.................**

**RCPE me h11**  **-----..........-----------.................................--....................--.................**

**RCPE me h12**  **-----..........-----------.................................--....................--.................**

**RCPE me h13**  **-----..........-----------.................................--....................--.................**

**RCPE me h14**  **-----..........-----------............................------------------------------------------....**

**RCPE me h15**  **-----..........-----------.................................--....................--.................**

**RCPE me h16**  **-----..........-----------.................................--....................--.................**

**RCPE me h17**  **-----..........-----------.................................--....................--.................**

**RCPE me h18**  **-----..........-----------.................................--....................--.................**

**RCPE me h19**  **-----..........-----------.................................--....................--.................**

**RCPE me h20**  **-----..........-----------.................................--....................--.................**

**RCPE si h1**  **AAAGG..........CTTATGCGTAC.....C..G..C........G..........--GT......T........A...G--.........G.......**

**RCPE si h2**  **AAAGG..........CTTATGCGTAC.....C..G..C........G..........--GT......T........A...G--.........G.......**

**RCPE si h3**  **AAAGG..........CTTATGCGTAC.....C..G..C........G..........--GT......T........A...G--A........G.......**

**RCPE si h4**  **AAAGG..........CTTATGCGTAC.....C..G..C........G..........--GT......T........A...G--A........G.......**

**RCPE si h5**  **AAAGG..........CTTATGCGTAC.....C..G..C........G..........--GT......T........A...G--.........G.......**

**RCPE si h6**  **AAAGG..........CTTATGCGTAC.....C..G..C........G..........--GT......T........A...G--.........G.......**

**RCPE si h7**  **AAAGG..........CTTATGCGTAC.....C..G..C........G..........--GT......T........A...G--.........G.......**

**RCPE si h8**  **AAAGG..........CTTATGCGTAC.....C..G..C........G..........--GT......T........A...G--A........G.......**

**RCPE si h9**  **AAAGG....T.....CTTATGCGTAC.....C..G..C........G..........--GT......T........A...G--.........G.......**

**RCPE si h10**  **AAAGG..........CTTATGCGTAC.....C..G..C........G..........--GT......T........A...G--A........G.......**

**RCPE si h11**  **AAAGG..........CTTATGCGTAC.....C..G..C........G..........--GT......T........A...G--.........G.......**

**RCPE si h12**  **AAAGG..........CTTATGCGTAC.....C..G..C........G..........--GT......T........A...G--.........G.......**

**RCPE si h13**  **AAAGG..........CTTATGCGTAC.....C..G..C........G..........--GT......T........A...G--.........G.......**

**RCPE se h1**  **AAAGG....T.....CTTATGCGTAC.....C..G..C........G..........--GT......T.....A..A...G--.........G.......**

**RCPE ma h1**  **AAAGG..........CTTATGCGTAC.....C..G..C........G..........--GT......T...-----------------------------**

**RCPE ya h1**  **T~~AAAA~~..T..~~T.~~...CTTGTGCTTGC.TT.CC....T..A......G......G...--GT......T.A......A..GTTG.C..-A...G.GT...T**

**RCPE ya h2**  **TAAAA..T..T....CTTGTGCTTGC.TT.CC....T.........G......G...--GT......T.A......A..GTAG.C..-A...G.GT...T**

**RCPE ya h3**  **TAAAA..T..T....CTTGTGCTTGC.TT.CC....T..A......G......G...--GT......T.A......A..GTTG.C..-A...G.GT...T**

**RCPE ya h4**  **TAAAA..T..T....CTTGTGCTTGC.TT.CC....T.........G......G...--GT......T.A......A..GTAG.C..-A...G.GT...T**

**RCPE ya h5**  **TAAAA..T..T....CTTGTGCTTGC.TT.CC....T..A......G......G...--GT......T.A......A..GTTG.C..-A...G.GT...T**

**RCPE ya h6**  **TAAAA..T..T....CTTGTGCTTGC.TT.CC....T..A......G......G...--GT......T.A......A..GTTG.C..-A...G.GT...T**

**RCPE ya h7**  **TAAAA..T..T....CTTGTGCTTGC.TT.CC....T..A......G......G...--GT......T.A......A..GTTG.C..-A...G.GT...T**

**RCPE ya h8**  **TAAAA..T..T....CTTGTGCTTGC.TT.CC....T..A......G......G...--GT......T.A......A..GTTG.C..-A...G.GT...T**

**RCPE ya h9**  **TAAAA..T..T....CTTGTGCTTGC.TT.CC....T.........G......G...--GT......T.A......A..GTAG.C..-A...G.GT...T**

**RCPE ya h10**  **TAAAA..T..T....CTTGTGCTTGC.TT.CC....T.........G......G...--GT......T.A......A..GTAG.C..-A...G.GT...T**

**RCPE ya h11**  **TAAAA..T..T....CTTGTGCTTGC.TT.CC....T.........G......G...--GT......T.A......A..GTAG.C..-A...G.GT...T**

**RCPE ya h12**  **TAAAA..T..T....CTTGTGCTTGC.TT.CC....T..A......G......G...--GT......T.A......A..GTTGCC..-A...G.GT...T**

**RCPE ya h13**  **TAAAA..T..T....CTTGTGCTTGC.TT.CC....T..A......G......G...--GT......T.A......A..GTTG.C..-A...G.GT...T**

**RCPE ya h14**  **TAAAA..T..T....CTTGTGCTTGC.TT.CC....T..A......G......G...--GT......T.A......A..GTTGCC..-A...G.GT...T**

**RCPE ya h15**  **TAAAA..T..T....CTTGTGCTTGC.TT.CC....T.........G......G...--GT......T.A......A..GTAG.C..-A...G.GT...T**

**RCPE ya h16**  **TAAAA..T..T....CTTGTGCTTGC.TT.CC....T.........G......G...--GT......T.A......A..GTAG.C..-A...G.GT...T**

**RCPE te h1**  **TAAAA..T..T....CTTGCGCTTGC.TT.CC....T....T....G..........--GT......T.A......A..GTTG.C..-AG..G.GTG..T**

**RCPE te h2**  **TAAAA..T..T....CTTGCGCTTGC.TT.CC....T....T....G..........--GT......T.A......A..GTTG.C..-AG..G.GTG..T**

**RCPE or h1**  **TAAAA..TG.---..CTTG------C.....C....T.----------------------------------....A.AGTTGCC.G-A...G.GTG..T**

**RCPE er h1**  **TAAAA..TG.T.-..CTTGTG----C.....C....T.----------------------------------....C.AGTTGCC.G-A...G.GTA..T**

**Fixed substitutions**  **1__11__11_2_1__1__12_11_1__11_11__1_111__1____1______1___1_1_______1_1_1_1__2_1121_11_1111__1_112__1**

**Polymorphic substitutions** **_________1^2^____________________________1______________1__________________________1_2________________**

**DNAse footprinting data ____________________________________________________________________________________________________**

310 320 330 340 350 360 370 380 390 400

....|....|....|....|....|....|....|....|....|....|....|....|....|....|....|....|....|....|....|....|

**RCPE me h1**  **~~AATG~~CT~~TTTATTTCTATTTCCGTTTC-----~~--------------------~~ATCAGTT~~---~~T~~G-------------------------------------**

**RCPE me h2**  **..........................-------------------------.......---..-------------------------------------**

**RCPE me h3**  **..........................-------------------------.......---..-------------------------------------**

**RCPE me h4**  **..........................-------------------------.......---..-------------------------------------**

**RCPE me h5**  **..........................-------------------------.......---..-------------------------------------**

**RCPE me h6**  **..........................-------------------------.......---..-------------------------------------**

**RCPE me h7**  **..........................-------------------------.......---..-------------------------------------**

**RCPE me h8**  **..........................-------------------------.......---..-------------------------------------**

**RCPE me h9**  **..........................-------------------------.......---..-------------------------------------**

**RCPE me h10**  **..........................-------------------------.......---..-------------------------------------**

**RCPE me h11**  **..........................-------------------------.......---..-------------------------------------**

**RCPE me h12**  **..........................-------------------------.......---..-------------------------------------**

**RCPE me h13**  **..........................-------------------------.......---..-------------------------------------**

**RCPE me h14**  **..........................-------------------------.......---..-------------------------------------**

**RCPE me h15**  **..........................-------------------------.......---..-------------------------------------**

**RCPE me h16**  **..........................-------------------------.......---..-------------------------------------**

**RCPE me h17**  **..........................-------------------------.......---..-------------------------------------**

**RCPE me h18**  **..........................-------------------------.......---..-------------------------------------**

**RCPE me h19**  **..........................-------------------------.......---..-------------------------------------**

**RCPE me h20**  **..........................-------------------------.......---..-------------------------------------**

**RCPE si h1**  **...TT.G.G.........A..C...T------------------TAAAAAA....C..---..-------------------------------------**

**RCPE si h2**  **...TT.G.G.........A..C...T------------------TAAAAAA....C..---..-------------------------------------**

**RCPE si h3**  **...TT.G.G.........A......T-------------------AAAAAA....C..---..-------------------------------------**

**RCPE si h4**  **...TT.G.G.........A......T-------------------AAAAAA....C..---..-------------------------------------**

**RCPE si h5**  **...TT.G.G.........A..C...T------------------TAAAAAA....C..---..-------------------------------------**

**RCPE si h6**  **...TT.GGG.........A......T-------------------AAAAAA....C..---..-------------------------------------**

**RCPE si h7**  **...TT.G.G.........A..C...T------------------TAAAAAA....C..---..-------------------------------------**

**RCPE si h8**  **...TT.GGG.........A......T-------------------AAAAAA....C..---..-------------------------------------**

**RCPE si h9**  **...TT.G.G.........A......T-------------------AAAAAA....C..---..-------------------------------------**

**RCPE si h10**  **...TT.G.G.........A......T-------------------AAAAAA....C..---..-------------------------------------**

**RCPE si h11**  **...TT.G.G.........A.....~~.TAATAG~~GCC-----------AAAATT..A.AA.---..-------------------------------------**

**RCPE si h12**  **...TT.G.G.........A..C...T------------------TAAAAAA....C..---..-------------------------------------**

**RCPE si h13**  **...TT.G.G.........A..C...T------------------TAAAAAA....C..---..-------------------------------------**

**RCPE se h1**  **...TT.G.G.....------.....T-------------------AAAAAA....C..---..-------------------------------------**

**RCPE ma h1**  **--------------------------------------------------------------.-------------------------------------**

**RCPE ya h1**  **..AT.A.AG.C...--------------------------------------------A--..AATTTCGTGA~~TTTCTTTTTCCA~~TGTCAT---------**

**RCPE ya h2**  **..AT.A.AG.C...--------------------------------------------A--..AATTTCGTGATATCTTTTTCCATGTCAT---------**

**RCPE ya h3**  **..AT.A.AG.C...--------------------------------------------A--..AATTTCGTGATTTCTTTTTCCATGTCAT---------**

**RCPE ya h4**  **..AT.A.AG.C...--------------------------------------------A--..AATTTCGTGATTTCTTTTTCCATGTCAT---------**

**RCPE ya h5**  **..AT.A.AG.C...--------------------------------------------A--..AATTTCGTGATTTCTTTTTCCATGTCAT---------**

**RCPE ya h6**  **..AT.A.AG.C...--------------------------------------------A--..AATTTCGTGATTTCTTTTTCCATGTCAT---------**

**RCPE ya h7**  **..AT.A.AG.C...--------------------------------------------A--..AATTTCGTGATTTCTTTTTCCATGTCAT---------**

**RCPE ya h8**  **..AT.A.AG.C...--------------------------------------------A--..AATTTCGTGATTTCTTTTTCCATGTCAT---------**

**RCPE ya h9**  **..AT.A.AG.C...--------------------------------------------A--..AATTTCGTGATTTCTTTTTCCATGTCAT---------**

**RCPE ya h10**  **..AT.A.AG.C...--------------------------------------------A--..AATTTCGTGATTTCTTTTTCCATGTCAT---------**

**RCPE ya h11**  **..AT.A.AG.C...--------------------------------------------A--..AATTTCGTGATTTCTTTTTCCATGTCAT---------**

**RCPE ya h12**  **..AT.A.AG.C...--------------------------------------------A--..AATTTCGTGATTTCTTTTTCCATGTCAT---------**

**RCPE ya h13**  **..AT.A.AG.C...--------------------------------------------A--..AATTTCGTGATTTCTTTTTCCATGTCAT---------**

**RCPE ya h14**  **..AT.A.AG.C...--------------------------------------------A--..AATTTCGTGATTTCTTTTTCCATGTCAT---------**

**RCPE ya h15**  **..AT.A.AG.C...--------------------------------------------A--..AATTTCGTGATTTCTTTTTCCATGTCAT---------**

**RCPE ya h16**  **..AT.A.AG.C...--------------------------------------------A--..AATTTCGTGATTTCTTTTTCCATGTCAT---------**

**RCPE te h1**  **..AT.A.AG.C..-------------AGAC----~~ATAAT~~-------~~GGAAA.AA~~G..GATG..AAT~~TTTGT~~GATTTCTTTTTCCGTGACAC---------**

**RCPE te h2**  **..AT.A.AG.C..-------------AGAC----ATAAT-------GGAAA.AAG..GATG..AATTTTGTGATTTCTTTTTCCGTGACAC---------**

**RCPE or h1**  **..ATTA.AG.C.GA------------~~AACA~~----~~GTAAT~~ACACATAG~~AGAA.AA~~G...-~~TG~~..AAT~~TTTATGGTTTTTTT~~GCTCGTGTCATAATTTGCTG**

**RCPE er h1**  **..ATTA.AG.C.A.------------AACA----TTAATATACAT~~AG~~AAAG.AAG...-TG..CATTTTATGATTTTTT-GTTCGTGTCATATTTTGCTG**

**Fixed substitutions**  **__1111111_1_223___1______11111____2____11____1111_1_1111_111___2____11__1___1__1111_1__1__111_______**

**Polymorphic substitutions** **_______1_____________1____1_________________1____11__1_11_________________1_________________________**

**DNAse footprinting data ____________________________________________________________________________________________________**

410 420 430 440 450 460 470 480 490 500

....|....|....|....|....|....|....|....|....|....|....|....|....|....|....|....|....|....|....|....|

**RCPE me h1**  **--C~~ATTTTATATTCCAAATTGT~~A~~TAATC~~C--------------~~TTTAAATT--ATTAAAATTGTT~~A~~TAGTG~~CACGG~~ACCT~~-----~~TC~~GAG~~CCTG~~C~~TT-AA~~**

**RCPE me h2**  **--...........................--------------........--...........................-----............-..**

**RCPE me h3**  **--.....A.....................--------------........--...................T.......-----............-..**

**RCPE me h4**  **--...........................--------------........--...........................-----............-..**

**RCPE me h5**  **--.....A.....................--------------........--...................T.......-----............-..**

**RCPE me h6**  **--...........................--------------........--...........................-----............-..**

**RCPE me h7**  **--...........................--------------........--...........................-----............-..**

**RCPE me h8**  **--...........................--------------........--...........................-----............-..**

**RCPE me h9**  **--...........................--------------........--...........................-----............-..**

**RCPE me h10**  **--..........................T--------------........--...........................-----............-..**

**RCPE me h11**  **--...........................--------------........--...........................-----............-..**

**RCPE me h12**  **--...........................--------------........--...........................-----............-..**

**RCPE me h13**  **--...........................--------------........--...........................-----............-..**

**RCPE me h14**  **--...........................--------------........--...........................-----............-..**

**RCPE me h15**  **--.....A.....................--------------........--...................T.......-----............-..**

**RCPE me h16**  **--.....................A.....--------------........--...........................-----............-..**

**RCPE me h17**  **--.....................A.....--------------........--...........................-----............-..**

**RCPE me h18**  **--.....A.....................--------------........--...................T.......-----............-..**

**RCPE me h19**  **--.....A.....................--------------........--...................T.......-----............-..**

**RCPE me h20**  **--...........................--------------........--...........................-----............-..**

**RCPE si h1**  **--.........GGGT.............TCC~~TAAATTT~~--~~TC~~T........--.......----------------....-----.......C.G..-..**

**RCPE si h2**  **--.........GGGT.............TCCTAAATTT--TCT........--.......----------------....-----.......C.G..-..**

**RCPE si h3**  **--.T.......GG...............TCCTAAATTT--TCT.......ATA..A....----------------.T..-----.......C.G..-..**

**RCPE si h4**  **--.T.......GG...............TCCTAAATTT--TCT.......ATA..A....----------------.T..-----.......C.G..-..**

**RCPE si h5**  **--.........GGGT.............TCCTAAATTT--TCT........--.......----------------....-----.......C.G..-..**

**RCPE si h6**  **--.........GG...............TCCTAAATTT--TCT........--.......----------------....-----.......C.G..-..**

**RCPE si h7**  **--.........GGGT.............TCCTAAATTT--TCT........--.......----------------....-----.......C.G..-..**

**RCPE si h8**  **--.T.......GG...............TCCTAAATTT--TCT.......ATA..A....----------------.T..-----.......C.G..-..**

**RCPE si h9**  **--.........GG...............TCCTAAATTT--TCT........--.......----------------....-----.......C.G..-..**

**RCPE si h10**  **--.T.......GG...............TCCTAAATTT--TCT.......ATA..A....----------------.T..-----.......C.G..-..**

**RCPE si h11**  **--.........GG...............TCCTAAATTT--TCT........--.......----------------....-----.........G..-..**

**RCPE si h12**  **--.........GGGT.............TCCTAAATTT--TCT........--.......----------------....-----.......C.G..-..**

**RCPE si h13**  **--.........GGGT.............TCCTAAATTT--TCT........--.......----------------....-----.......C.G..-..**

**RCPE se h1**  **--.........GG...............TCCTAAATTT--TCT........--.......----------------....-----.........G..-..**

**RCPE ma h1**  **--.........GG.............-.TCCTAAATTT--TCT........--.......----------------....-----.........G..-..**

**RCPE ya h1**  **--.........AA..C........G...TCTAA~~AA~~---------.......--.......---....GA..TTG......-----.TA.........T..**

**RCPE ya h2**  **--.........AA..C..G.....G...TCTAAAA~~TTTGT~~TC~~A....~~....--.......---....GA.CTTG......-----.TA.........T..**

**RCPE ya h3**  **--.........AA..C........G...TCTAAAATTTGTTCA........--.......---....GA..TTG......-----.GA.........T..**

**RCPE ya h4**  **--.........AA..C..G.....G...TCTAAAATTTGTTCA........--.......---....GA..TTG......-----.TA.........T..**

**RCPE ya h5**  **--.........AA..C........G....CTAAAATTTGTTCA........--.......---....GA..TTG......-----.TA.........T..**

**RCPE ya h6**  **--.........AA..C........G...TCTAAAATTTGTTCA........--.......---....GA..TTG......-----.TA.........T..**

**RCPE ya h7**  **--.........AA..C........G...TCTAAAATTTGTTCA........--.......---....GA..TTG......-----.GA.........T..**

**RCPE ya h8**  **--.........AA..C........G...TCTAAAATTTGTTCA........--.......---....GA..TTG......-----.GA.........T..**

**RCPE ya h9**  **--.........AA..C..G.....G...TCTAAAATTTGTTCA........--.......---....GA..TTG......-----.TA.........T..**

**RCPE ya h10**  **--.........AA..C..G.....G...TCTAAAATTTGTTCA........--.......---....GA..TTG......-----.GA.........T..**

**RCPE ya h11**  **--.........AA..C..G.....G...TCTAAAATTTGTTCA........--.......---....GA..TTG......-----.TA.........T..**

**RCPE ya h12**  **--.........AA..C........G...TCTAAAATTTGTTCA........--.......---....GA..TTG......-----.GA.........T..**

**RCPE ya h13**  **--.....-...AA..C..G.....G...TCTAAAATTTGTTCA........--.......---....GA..TTG......-----.TA.........T..**

**RCPE ya h14**  **--.........AA..C........G...TCTAAAATTTGTTCA........--.......---....GA..TTG......-----.GA.........T..**

**RCPE ya h15**  **--.........AA..C..G.....G...TCTAAAATTTGTTCA........--.......---....GA..TTG......-----.TA.........T..**

**RCPE ya h16**  **--.........AA..C..G.....G...TCTAAAATTTGTTCA........--.......---....GA..TTG......-----.TA.........T..**

**RCPE te h1**  **--.........AA..C........G...TCTACAAT~~TTTGT~~CA........--...G...---....GA...TG......~~GCGG~~G.----.......T..**

**RCPE te h2**  **--.........AA..C........G...TCTACAATTTTGTCA........--...G...---....GA...TG......GCGGG.----.......T..**

**RCPE or h1**  **TG...A.....AA.A..T......G...TCT~~TAAAT~~AT--TCT........--...C...A.A...A.A..TT....G..-----.AA.........~~T~~TT**

**RCPE er h1**  **TG...A..T..AATAC.------------------------CT...G...----------A.A.....A..TT.......-----.TA.......-----**

**Fixed substitutions**  **_____1__1__22111^1^2______1___11111___1_21__1___1___1_____2___2_1___111__1_1___1__1_____31______11_111**

**Polymorphic substitutions ___1___2_____11___1____1____2______1______________11___1________1_____1_1____1________1_____1_______**

**DNAse footprinting data ____________________________________________________________________________________________________**

510 520 530 540 550 560 570 580 590 600

....|....|....|....|....|....|....|....|....|....|....|....|....|....|....|....|....|....|....|....|

**RCPE me h1**  **~~AGAA~~--TCGAA~~ACT--~~---~~TTAAATTAC~~T~~A~~CAC~~AAGGTA~~CAAGGTATTAGGAGA~~ATT~~--------------~~AACATAGTTTCAC~~-~~ATG~~A~~CATTATTTATC~~**

**RCPE me h2**  **....--........-----......................................--------------.............-...............**

**RCPE me h3**  **....--........-----......................................--------------.............-...............**

**RCPE me h4**  **....--........-----......................................--------------.............-...............**

**RCPE me h5**  **....--........-----......................................--------------.............-...............**

**RCPE me h6**  **....--........-----......................................--------------.............-...............**

**RCPE me h7**  **....--........-----......................................--------------.............-...............**

**RCPE me h8**  **....--........-----......................................--------------.............-...............**

**RCPE me h9**  **....--........-----......................................--------------.............-...............**

**RCPE me h10**  **....--........-----......................................--------------.............-...............**

**RCPE me h11**  **....--........-----......................................--------------.............-...............**

**RCPE me h12**  **....--........-----..........................--------------------------.............-...............**

**RCPE me h13**  **....--........-----......................................--------------.............-...............**

**RCPE me h14**  **....--........-----......................................--------------.............-...............**

**RCPE me h15**  **....--........-----......................................--------------.............-...............**

**RCPE me h16**  **....--........-----..........................--------------------------.............-...............**

**RCPE me h17**  **....--........-----..........................--------------------------.............-...............**

**RCPE me h18**  **....--........-----......................................--------------.............-...............**

**RCPE me h19**  **....--........-----......................................--------------.............-...............**

**RCPE me h20**  **....--........-----......................................--------------.............-...............**

**RCPE si h1**  **.T..-------------------------.....GAA..--------------------------------....A.....A.---.A............**

**RCPE si h2**  **.T..-------------------------.....GAA..--------------------------------....A.....A.---.A............**

**RCPE si h3**  **.T..-------------------------.....GAA..--------------------------------....A.....A.---.A............**

**RCPE si h4**  **.T..-------------------------.....GAA..--------------------------------....A.....A.---.A............**

**RCPE si h5**  **.T..-------------------------.....GAA..--------------------------------....A.....A.---.A............**

**RCPE si h6**  **.T..-------------------------.....GAA..--------------------------------....A.....A.---.A............**

**RCPE si h7**  **.T..-------------------------.....GAA..--------------------------------....A.....A.---.A............**

**RCPE si h8**  **.T..-------------------------.....GAA..--------------------------------....A.....A.---.A............**

**RCPE si h9**  **.T..-------------------------.....GAA..--------------------------------....A.....A.---.A............**

**RCPE si h10**  **.T..-------------------------.....GAA..--------------------------------....A.....A.---.A............**

**RCPE si h11**  **.T..-------------------------.....GAA..--------------------------------....A.....A.---.A............**

**RCPE si h12**  **.T..-------------------------.....GAA..--------------------------------....A.....A.---.A............**

**RCPE si h13**  **.T..-------------------------.....GAA..--------------------------------....A.....A.---.A............**

**RCPE se h1**  **.T..-------------------------.....GAA..--------------------------------....A.....A.---AA............**

**RCPE ma h1**  **.T..-------------------------.....GAA..--------------------------------....A.....A.---.A............**

**RCPE ya h1**  **.TTT---.A...T.TGAAA.A.C..-----....G..C.------------------TCCTTAGAT~~GAAAA..~~T..T...-A..CG.AG..........T**

**RCPE ya h2**  **.TTT----------------~~A~~....A---.....G....------------------TCCT~~TAAAT~~GAA~~A~~--------------------------~~...A~~**

**RCPE ya h3**  **.TTT----------------A....A---.....G....------------------TCCTTAAATGAAA--------------------------...A**

**RCPE ya h4**  **.TTT----------------A....A---.....G....------------------TCCTTAAATGAAA--------------------------...A**

**RCPE ya h5**  **.TTT---.A...T.TGAAA.A.C..-----....G....------------------TCCTTAGATGAAAA..T..T...-A..CG.AG..........T**

**RCPE ya h6**  **.TTT---.A...T.TTAAA.A.C..-----....G....------------------TCCTTAGATGAAAA..T..T...-A..CG.AG..........T**

**RCPE ya h7**  **.TTT----------------A....A---.....G....------------------TCCTTAAATGAAA--------------------------...A**

**RCPE ya h8**  **.TTT----------------A....A---.....G....------------------TCCTTAAATGAAA--------------------------...A**

**RCPE ya h9**  **.TTT---.A...T.TGAAA.A.C..-----....G..C.------------------TCCTTAGATGAAAA..T..T...-A..-G.AG..........T**

**RCPE ya h10**  **.TTT----------------A....A---.....G....------------------TCCTTAAATGAAA--------------------------...A**

**RCPE ya h11**  **.TTT---.A...T.TGAAA.A.C..-----....G..C.------------------TCCTTAGATGAAAA..T..T...-A..CG.AG..........T**

**RCPE ya h12**  **.TTT----------------A....A---.....G....------------------TCCTTAAATGAAA--------------------------...A**

**RCPE ya h13**  **.TTT---.A...T.TGAAA.A.C..-----....G..C.------------------TCCTTAGATGAAAA..T..T...-A..CG.AG..........T**

**RCPE ya h14**  **.TTT----------------A....A---.....G....------------------TCCTTAAATGAAA--------------------------...A**

**RCPE ya h15**  **.TTT----------------A....A---.....G....------------------TCCTTAAATGAAA--------------------------...A**

**RCPE ya h16**  **.TTT----------------A....A---.....G....------------------TCCTTAAATGAAA--------------------------...A**

**RCPE te h1**  **-TT.--......~~T.TAT~~AA.A....A..A.....G....------------------TCCTTAAATGAAAA..T..T...-AG.CG.A...........T**

**RCPE te h2**  **-TT.--......T.TATAA.A....A..A.....G....------------------TCCTTAAATGAAAA..T..T...-AG.CG.A...........T**

**RCPE or h1**  **-TT.~~T~~T..--..T.-----AA.......A..T.......------------------TCCCTATAAGTAAAG.T......-A.-----------------**

**RCPE er h1**  **-----------.T.-----AA.......A....C.....------------------TCCCTATAAGTAGC..T......CA------------------**

**Fixed substitutions**  **11111___1_____111__11_____1_1__1_1111__1_________________1__1__1_1_1_111_1_11___21221_111__________1**

**Polymorphic substitutions** **____2___1___1_11______1__1^2^__1_______1_______1_________________1______1_____________1______________1**

**DNAse footprinting data ____________________________________________________________________________________________________**

610 620 630 640 650 660 670 680 690 700

....|....|....|....|....|....|....|....|....|....|....|....|....|....|....|....|....|....|....|....|

**RCPE me h1**  **~~AATAGTT~~--~~TTTAAC~~-~~TGATG~~---A~~CACTCTGACATTTTTCAAAAA~~GC~~CATCAATC~~TTTGAGCTCAGTTGGAGCGTCCGT~~AACAGAA~~GGTGTTAACGAGG**

**RCPE me h2**  **.......--......-.....---............................................................................**

**RCPE me h3**  **.......--......-.....---............................................................................**

**RCPE me h4**  **.......--......-.....---............................................................................**

**RCPE me h5**  **.......--......-.....---............................................................................**

**RCPE me h6**  **.......--......-.....---............................................................................**

**RCPE me h7**  **.......--......-.....---............................................................................**

**RCPE me h8**  **.......--......-.....---............................................................................**

**RCPE me h9**  **.......--......-.....---..................................................A.........................**

**RCPE me h10**  **.......--......-.....---..................................................A.........................**

**RCPE me h11**  **.......--......-.....---............................................................................**

**RCPE me h12**  **.......--......-.....---............................................................................**

**RCPE me h13**  **.......--......-.....---............................................................................**

**RCPE me h14**  **.......--......-.....---............................................................................**

**RCPE me h15**  **.......--......-.....---............................................................................**

**RCPE me h16**  **.......--......-.....---............................................................................**

**RCPE me h17**  **.......--......-.....---............................................................................**

**RCPE me h18**  **.......--......-.....---............................................................................**

**RCPE me h19**  **.......--......-.....---............................................................................**

**RCPE me h20**  **.......--......-.....---............................................................................**

**RCPE si h1**  **.....A.--.....G-.....---...............G............................................................**

**RCPE si h2**  **.....A.--.....G-.....---...............G............................................................**

**RCPE si h3**  **.....A.--.....G-.....---...............G............................................................**

**RCPE si h4**  **.....A.--.....G-.....---...............G............................................................**

**RCPE si h5**  **.....A.--.....G-.....---...............G............................................................**

**RCPE si h6**  **.....A.--.....G-.....---...............G............................................................**

**RCPE si h7**  **.....A.--.....G-.....---...............G............................................................**

**RCPE si h8**  **.....A.--.....G-.....---...............G............................................................**

**RCPE si h9**  **.....A.--.....G-.....---...............G............................................................**

**RCPE si h10**  **.....A.--.....G-.....---...............G............................................................**

**RCPE si h11**  **.....A.--.....G-.....---...............G............................................................**

**RCPE si h12**  **.....A.--.....G-.....---...............G............................................................**

**RCPE si h13**  **.....A.--.....G-.....---...............G............................................................**

**RCPE se h1**  **.....A.--.....G-.....---...............G..................--........................................**

**RCPE ma h1**  **.....A.--.....G-.....---...............G............................................................**

**RCPE ya h1**  **....TACAC.....G-ATT.AG---..T.T.A.A......T..............................G......T.C..T................**

**RCPE ya h2**  **~~...~~.TACAC.....G-AT...G---..T.TAA.A......T..........T...................G......T.C..T................**

**RCPE ya h3**  **....TACAC.....G-AT...G---..T.TAA.A......T..........T...................G......T.C..T................**

**RCPE ya h4**  **....TACAC.....G-AT...G---..T.TAA.A......T..........T....C..............G......T.C..T................**

**RCPE ya h5**  **....TACAC.....G-ATT..G---..T.TAA.A......T..............................G......T.C..T................**

**RCPE ya h6**  **....TACAC.....G-ATT.AG---..T.TAA.A......T..............................G......T.C..T................**

**RCPE ya h7**  **....TACAC.....G-AT...G---..T.TAA.A......T..........T...................G......T.C..T................**

**RCPE ya h8**  **....TACAC.....G-AT...G---..T.TAA.A......T..........T...................G......T.C..T................**

**RCPE ya h9**  **....TACAC.....G-ATT..G---..T.T.A.A......T..............................G......T.C..T................**

**RCPE ya h10**  **....TACAC.....G-AT...G---..T.TAA.A......T..........T...................G......T.C..T................**

**RCPE ya h11**  **....TACAC.....G-ATT.AG---..T.T.A.A......T..............................G......T.C..T................**

**RCPE ya h12**  **....TACAC.....G-AT...G---..T.TAA.A......T..........T....C..............G......T.C..T................**

**RCPE ya h13**  **....TACAC.....G-ATT..G---..T.T.A.A......T..............................G......T.C..T................**

**RCPE ya h14**  **....TACAC.....G-AT...G---..T.TAA.A......T..........T....C..............G......T.C..T................**

**RCPE ya h15**  **....TACAC.....G-AT...G---..T.TAA.A......T..........T....C..............G......T.C..T................**

**RCPE ya h16**  **....TACAC.....G-AT...G---..T.TAA.A......T..........T....C..............G......T.C..T................**

**RCPE te h1**  **....TACAC....GG-.T...G---..T.T.A...................T...................G........C..T................**

**RCPE te h2**  **....TACAC....GG-.T...G---..T.T.A...................T...................G........C..T................**

**RCPE or h1**  **T....C.AA.A...T~~A~~.AG..ACT...T...............................-...........G.....T..C..G................**

**RCPE er h1**  **T......AA.AC..TA.AG..ACT...T................G..............C...........G.....T..C..T................**

**Fixed substitutions**  **1___12111_11_121121__21____1_1_1_1_____11___1_____________12___________1_____11_1__2________________**

**Polymorphic substitutions** **__________________1_1_________1____________________1^2^___1_________________1_________________________**

**DNAse footprinting data ____________________________________________________________________________________________________**

710 720 730 740 750 760 770 780 790 800

....|....|....|....|....|....|....|....|....|....|....|....|....|....|....|....|....|....|....|....|

**RCPE me h1**  **TCCTAGCTGAG~~ATTTAAACAA~~TTCGAA~~ATTTATATCTAGG~~GGC~~CATAAGCAG~~TA~~CGAT-~~GCGA~~TGATG~~CACA~~CAGTTCACAT~~TTCATTCAGCTTAC~~GGGG~~**

**RCPE me h2**  **..........................................................-.........................................**

**RCPE me h3**  **..........................................................-.........................................**

**RCPE me h4**  **..........................................................-.........................................**

**RCPE me h5**  **..........................................................-.........................................**

**RCPE me h6**  **..........................................................-.........................................**

**RCPE me h7**  **..........................................................-.........................................**

**RCPE me h8**  **..........................................................-.........................................**

**RCPE me h9**  **..........................................................-........................-----............**

**RCPE me h10**  **..........................................................-........................-----............**

**RCPE me h11**  **..........................................................-.........................................**

**RCPE me h12**  **..........................................................-.........................................**

**RCPE me h13**  **..........................................................-.........................................**

**RCPE me h14**  **..........................................................-.....A.......T...........................**

**RCPE me h15**  **..........................................................-.........................................**

**RCPE me h16**  **..........................................................-.........................................**

**RCPE me h17**  **..........................................................-.........................................**

**RCPE me h18**  **..........................................................-................A........................**

**RCPE me h19**  **..........................................................-................A........................**

**RCPE me h20**  **..........................................................-.........................................**

**RCPE si h1**  **...............G....G.........................T........T..T......-----..T....T......................**

**RCPE si h2**  **...............G....G.........................T........T..T......-----..T....T......................**

**RCPE si h3**  **...............G....G.................TT......T........T..T......-----..T....T......................**

**RCPE si h4**  **...............G....G.................TT......T........T..T......-----..T....T......................**

**RCPE si h5**  **...............G....G.........................T........T..T......-----..T....T......................**

**RCPE si h6**  **...............G....G.........................C........T..T......-----..T....T......................**

**RCPE si h7**  **...............G....G.........................T........T..T......-----..T....T......................**

**RCPE si h8**  **...............G....G.................TT......T........T..T......-----..T....T......................**

**RCPE si h9**  **...............G..............................T........T..T......-----..T....T......................**

**RCPE si h10**  **...............G....G.........................T........T..T......-----..T....T......................**

**RCPE si h11**  **...............G....G.................TT......T........T..T......-----..T....T......................**

**RCPE si h12**  **...............G....G.........................T........T..T......-----..T....T......................**

**RCPE si h13**  **...............G....G.........................T........T..T......-----..T....T......................**

**RCPE se h1**  **...............G....G.........AT..............T........T..T......-----..T....T.....................-**

**RCPE ma h1**  **...............G....G.........................T........T..T......-----..T....T......................**

**RCPE ya h1**  **...A.........C.G.......T.....G..............G...A...A..C..T......-----...CA...C.....................**

**RCPE ya h2**  **...A.........C.G.......T.....G..............G...A...A..C..T......-----...CA...C.....................**

**RCPE ya h3**  **...A.........C.T.......T.....G..............G...A...A..C..T......-----...CA...C.....................**

**RCPE ya h4**  **...A.........C.G.......T.....G..............GA..A...A..C..T......-----...CA...C.....................**

**RCPE ya h5**  **...A.........C.G.......T.....G..............GA..A...A..C..T......-----...CA...C.....................**

**RCPE ya h6**  **...A.........C.G.......T.....G..............G...A...A..C..T......-----...CA...C.....................**

**RCPE ya h7**  **...A.........C.T.......T.....G..............G...A...A..C..T......-----...CA...C.....................**

**RCPE ya h8**  **...A.........C.T.......T.....G..............G...A...A..C..T......-----...CA...C.....................**

**RCPE ya h9**  **...A.........C.G.......T.....G..............G...A...A..C..T......-----...CA...C.....................**

**RCPE ya h10**  **...A.........C.G.......T.....G..............G...A...A..C..T......-----...CA...C.....................**

**RCPE ya h11**  **...A.........C.T.......T.....G..............G...A...A..C..T......-----...CA...C.....................**

**RCPE ya h12**  **...A.........C.T.......T.....G..............G...A...A..C..T......-----...CA...C.....................**

**RCPE ya h13**  **...A.........C.G.......T.....G..............G...A...A..C..T......-----...CA...C.....................**

**RCPE ya h14**  **...A.........C.G.......T.....G..............G...A...A..C..T......-----...CA...C.....................**

**RCPE ya h15**  **...A.........C.G.......T.....G..............GA..A...A..C..T......-----...CA...C.....................**

**RCPE ya h16**  **...A.........C.G.......T.....G..............GA..A...A..C..T......-----...TA...C.....................**

**RCPE te h1**  **...A...................T.....G..........C-..G...A...A..C..T..A...-----T..CA...C..C..................**

**RCPE te h2**  **...A...................T.....G..........C-..G...A...A..C..T..A...-----T..CA...C..C..................**

**RCPE or h1**  **...A...........G...........-.AC....T..T------------------.T......-----....A...C.....................**

**RCPE er h1**  **...A...........G...G.........G.....T........G...A......C..T......-----A...A...C.....................**

**Fixed substitutions**  **___1_________1_1^1^__1_______1_221___1__1111__1_1_1___1__2__1__1___1____2_111__11__1__________________1**

**Polymorphic substitutions** **_______________1____1^2^________________11_____11_________________1_______11_1_______1________________**

**DNAse footprinting data ____________________________________________________________________S_______________________________**

810 820 830 840 850 860 870 880 890 900

....|....|....|....|....|....|....|....|....|....|....|....|....|....|....|....|....|....|....|....|

**RCPE me h1**  **~~T~~CA~~TCATT~~GCT~~ATTTAAAA~~GCGG~~CAGGAC~~AATTCGAATAAGTGTGA~~CAGGA~~GC~~AATTAC~~AGCCTTA~~TCCTGATGTCTTC~~GATGTCAACAC~~ACCAG~~TGCTC**

**RCPE me h2**  **....................................................................................................**

**RCPE me h3**  **............----......C........................................................A....................**

**RCPE me h4**  **....................................................................................................**

**RCPE me h5**  **............----....................................................................................**

**RCPE me h6**  **............----....................................................................................**

**RCPE me h7**  **....................................................................................................**

**RCPE me h8**  **....................................................................................................**

**RCPE me h9**  **....................................................................................................**

**RCPE me h10**  **....................................................................................................**

**RCPE me h11**  **....................................................................................................**

**RCPE me h12**  **....................................................................................................**

**RCPE me h13**  **....................................................................................................**

**RCPE me h14**  **....................................................................................................**

**RCPE me h15**  **....................................................................................................**

**RCPE me h16**  **....................................................................................................**

**RCPE me h17**  **............----....................................................................................**

**RCPE me h18**  **....................................................................................................**

**RCPE me h19**  **....................................................................................................**

**RCPE me h20**  **............----....................................................................................**

**RCPE si h1**  **................................................................G..........................A........**

**RCPE si h2**  **..............A.................................................G..........................A........**

**RCPE si h3**  **................................................................G..........................A........**

**RCPE si h4**  **................................................................G..........................A........**

**RCPE si h5**  **................................................................G..........................A........**

**RCPE si h6**  **................................................................G..........................A........**

**RCPE si h7**  **..............A.................................................G..........................A........**

**RCPE si h8**  **................................................................G..........................A........**

**RCPE si h9**  **................................................................G..........................A........**

**RCPE si h10**  **................................................................G..........................A........**

**RCPE si h11**  **................................................................G..........................A........**

**RCPE si h12**  **................................................................G..........................A........**

**RCPE si h13**  **................................................................G..........................A........**

**RCPE se h1**  **...............T..........A..........................C..........G..........................A........**

**RCPE ma h1**  **................................................................G..........................A........**

**RCPE ya h1**  **.......C......G......................................G..........G.....A....T...............A........**

**RCPE ya h2**  **.......C......G......................................G..........G.....A....T...............A........**

**RCPE ya h3**  **.......C......G......................................G..........G.....A....T...............A........**

**RCPE ya h4**  **......AC......G......................................G..........G.....A....T...............A........**

**RCPE ya h5**  **......AC......G......................................G..........G.....A....T...............A........**

**RCPE ya h6**  **.......C......G......................................G..........G.....A....T...............A........**

**RCPE ya h7**  **.......C......G......................................G..........G.....A....T...............A........**

**RCPE ya h8**  **.......C......G......................................G..........G.....A....T...............A........**

**RCPE ya h9**  **......AC......G......................................G..........G.....A....T...............A........**

**RCPE ya h10**  **.......C......G......................................G..........G.....A....T...............A........**

**RCPE ya h11**  **.......C......G......................................G..........G.....A....T...............A........**

**RCPE ya h12**  **.......C......G......................................G..........G.....A....T...............A........**

**RCPE ya h13**  **......AC......G......................................G..........G.....A....T...............A........**

**RCPE ya h14**  **.......C......G......................................G..........G.....A....T...............A........**

**RCPE ya h15**  **......AC......G......................................G..........G.....A....T..T............A........**

**RCPE ya h16**  **......AC......G......................................G..........G.....A....T..T............A........**

**RCPE te h1**  **....A..C......G.................................................G.....A....T...............A........**

**RCPE te h2**  **....A..C......G.................................................G.....A....T...............A........**

**RCPE or h1**  **.......C......G...G.............................................G.....A....................A........**

**RCPE er h1**  **.......C......G..................G...............A.............AG.....A....................A........**

**Fixed substitutions**  **____1__1______11__1_______1______1_______________1___2_________11_____1____1_______________1________**

**Polymorphic substitutions** **______1_____1_1_______1_______________________________________________________11____________________**

**DNAse footprinting data ____________________PPPPPPPPPPPPPP__________PPPPPPPPPPPPPP_PPPPPPPPPPPPPPPP_________________________**

910 920 930 940 950 960 970 980 990 1000

....|....|....|....|....|....|....|....|....|....|....|....|....|....|....|....|....|....|....|....|

**RCPE me h1**  **AAGACATCGCAGGCAATTCAAGGATATG~~TAGGA~~CGCA~~CAGGA~~CCT~~CGAACAG~~A~~AGCCAA~~GGACACAGGCGAC~~GCGTG~~ACGCATTG~~GGAAAA~~TA~~TTTGT~~AC**

**RCPE me h2**  **....................................................................................................**

**RCPE me h3**  **....................................................................................................**

**RCPE me h4**  **....................................................................................................**

**RCPE me h5**  **....................................................................................................**

**RCPE me h6**  **....................................................................................................**

**RCPE me h7**  **....................................................................................................**

**RCPE me h8**  **....................................................................................................**

**RCPE me h9**  **....................................................................................................**

**RCPE me h10**  **....................................................................................................**

**RCPE me h11**  **....................................................................................................**

**RCPE me h12**  **....................................................................................................**

**RCPE me h13**  **....................................................................................................**

**RCPE me h14**  **....................................................................................................**

**RCPE me h15**  **....................................................................................................**

**RCPE me h16**  **....................................................................................................**

**RCPE me h17**  **....................................................................................................**

**RCPE me h18**  **....................................................................................................**

**RCPE me h19**  **....................................................................................................**

**RCPE me h20**  **....................................................................................................**

**RCPE si h1**  **....................G...............................................................................**

**RCPE si h2**  **....................G...............................................................................**

**RCPE si h3**  **....................G...............................................................................**

**RCPE si h4**  **....................G...............................................................................**

**RCPE si h5**  **....................G.............................G.................................................**

**RCPE si h6**  **....................G...............................................................................**

**RCPE si h7**  **....................G...............................................................................**

**RCPE si h8**  **....................G...............................................................................**

**RCPE si h9**  **....................G.............................G.................................................**

**RCPE si h10**  **....................G...............................................................................**

**RCPE si h11**  **....................G...............................................................................**

**RCPE si h12**  **....................G...............................................................................**

**RCPE si h13**  **....................G...............................................................................**

**RCPE se h1**  **....................G...............................................................................**

**RCPE ma h1**  **....................G...............................................................................**

**RCPE ya h1**  **.........................G..C....................................................................C..**

**RCPE ya h2**  **.........................G..C....................................................................C..**

**RCPE ya h3**  **.........................G..C....................................................................C..**

**RCPE ya h4**  **.........................G..C....................................................................C..**

**RCPE ya h5**  **.........................G..C....................................................................C..**

**RCPE ya h6**  **.........................G..C....................................................................C..**

**RCPE ya h7**  **.........................G..C....................................................................C..**

**RCPE ya h8**  **.........................G..C....................................................................C..**

**RCPE ya h9**  **.........................G..C....................................................................C..**

**RCPE ya h10**  **.........................G..C....................................................................C..**

**RCPE ya h11**  **.........................G..C....................................................................C..**

**RCPE ya h12**  **.........................G..C....................................................................C..**

**RCPE ya h13**  **.........................G..C....................................................................C..**

**RCPE ya h14**  **.........................G..C....................................................................C..**

**RCPE ya h15**  **.........................G..C....................................................................C..**

**RCPE ya h16**  **.........................G..C....................................................................C..**

**RCPE te h1**  **.................................................................................................C..**

**RCPE te h2**  **.......................................................AA............................A...........C..**

**RCPE or h1**  **..........C.T.GT............C..........AT.A..........................A...........................C..**

**RCPE er h1**  **..........C.T..T............C..........A..G......................G.A.A...........................C..**

**Fixed substitutions**  **__________1_1_11____1____1__1^1^_________11_2______________________1_1_1___________________________1__**

**Polymorphic substitutions** **__________________________________________________1____11____________________________1______________**

**DNAse footprinting data _____________PPPPPPPPPPPPPPPPPPPPPPPPPPPPPPPPPPPP_PPPPPPPPPPPPPPPPPPP_______________________________**

1010 1020 1030 1040 1050 1060 1070 1080 1090 1100

....|....|....|....|....|....|....|....|....|....|....|....|....|....|....|....|....|....|....|....|

**RCPE me h1**  **AA~~AACAT~~TGA~~GGATA~~TCT~~CAAAA~~GTAGCACAGCGTTTCGGC--------ATCCTTGACTTTGATTGGTGCCGATCCAAGGACG~~AAACGCTATAT~~CCACCC**

**RCPE me h2**  **.........................................--------...................................................**

**RCPE me h3**  **.........................................--------...................................................**

**RCPE me h4**  **.........................................--------...................................................**

**RCPE me h5**  **.........................................--------...................................................**

**RCPE me h6**  **.........................................--------...................................................**

**RCPE me h7**  **.........................................--------...................................................**

**RCPE me h8**  **.........................................--------...................................................**

**RCPE me h9**  **.........................................--------...................................................**

**RCPE me h10**  **.........................................--------...................................................**

**RCPE me h11**  **.........................................--------...................................................**

**RCPE me h12**  **.........................................--------...................................................**

**RCPE me h13**  **.........................................--------...................................................**

**RCPE me h14**  **.........................................--------...................................................**

**RCPE me h15**  **.........................................--------...................................................**

**RCPE me h16**  **.........................................--------...................................................**

**RCPE me h17**  **.........................................--------...................................................**

**RCPE me h18**  **.........................................--------...................................................**

**RCPE me h19**  **.........................................--------...................................................**

**RCPE me h20**  **.........................................--------...................................................**

**RCPE si h1**  **.............A....G.....C..........C.....--------...................................................**

**RCPE si h2**  **.............A....G.....C..........C.....--------...................................................**

**RCPE si h3**  **.............A....G.....C..........C.....--------...................................................**

**RCPE si h4**  **.............A....G.....C..........C.....--------...................................................**

**RCPE si h5**  **.............A....G.....C..........C.....--------...................................................**

**RCPE si h6**  **.............A....G.....C..........C.....--------...................................................**

**RCPE si h7**  **.............A....G.....C..........C.....--------...................................................**

**RCPE si h8**  **.............A....G.....C..........C.....--------...................................................**

**RCPE si h9**  **.............A....G.....C..........C.....--------...................................................**

**RCPE si h10**  **.............A....G.....C..........C.....--------...................................................**

**RCPE si h11**  **.............A....G.....C..........C.....--------...................................................**

**RCPE si h12**  **.............A....G.....C..........C.....--------...................................................**

**RCPE si h13**  **.............A....G.....C..........C.....--------...................................................**

**RCPE se h1**  **.............A....G.....C..........C.....--------............C......................................**

**RCPE ma h1**  **.............A....G.....C..........C.....--------...................................................**

**RCPE ya h1**  **......G......A..........C..........C..AT.--------...........G.....A.................................**

**RCPE ya h2**  **......G......A..........C..........C..AT.--------...........G.....A.................................**

**RCPE ya h3**  **......G......A..........C..........C..AT.--------...........G.....A.................................**

**RCPE ya h4**  **......G......A..........C..........C..AT.--------...........G.....A.................................**

**RCPE ya h5**  **......G......A..........C..........C..AT.--------...........G.....A.................................**

**RCPE ya h6**  **......G......A..........C..........C..AT.--------...........G.....A.................................**

**RCPE ya h7**  **......G......A..........C..........C..AT.--------...........G.....A.................................**

**RCPE ya h8**  **......G......A..........C..........C..AT.--------...........G.....A.................................**

**RCPE ya h9**  **......G......A..........C..........C..AT.--------...........G.....A.................................**

**RCPE ya h10**  **......G......A..........C..........C..AT.--------...........G.....A.................................**

**RCPE ya h11**  **......G......A..........C..........C..AT.--------...........G.....A.................................**

**RCPE ya h12**  **......G......A..........C..........C..AT.--------...........G.....A.................................**

**RCPE ya h13**  **......G......A..........C..........C..AT.--------...........G.....A.................................**

**RCPE ya h14**  **......G......A..........C..........C..AT.--------...........G.....A.................................**

**RCPE ya h15**  **......G......A..........C..........C..AT.--------...........G.....A....................C............**

**RCPE ya h16**  **......G......A..........C..........C..AT.--------...........G.....A.................................**

**RCPE te h1**  **......G......A.....G....C..........C..AT.--------.................A.............G...................**

**RCPE te h2**  **.....GG......A.....G....C..........C..AT.--------.................A.............G...................**

**RCPE or h1**  **.............A..........C........A.C..A..ATCGCAGC..................................CGT..............**

**RCPE er h1**  **.............A..........C........ACC..A..--------....................................T..............**

**Fixed substitutions**  **______1______1____11____1________111__11_1__________________11____1_____________1__111______________**

**Polymorphic substitutions** **_____1_________________________________________________________________________________1____________**

**DNAse footprinting data _________________________________________________PPPPPPPPPPP___________PPPPPPPPPPPPPPPPPPPPPPPP_____**

1110 1120 1130 1140 1150 1160 1170 1180 1190 1200

....|....|....|....|....|....|....|....|....|....|....|....|....|....|....|....|....|....|....|....|

**RCPE me h1**  **ATTGAGA~~TGACC~~TCCGGAGGAGTCGTCGGTGGGCCTCC~~GATCG~~AA~~GGTCACCCAGAAA~~TGCA~~TCCTG~~TCTGGC~~TTTCT~~C~~TTCAA~~TCT~~ATAGT~~GA~~ATTTTA~~**

**RCPE me h2**  **.......C...........................................................................G................**

**RCPE me h3**  **.......C...........................................................................G..............A.**

**RCPE me h4**  **.......C...........................................................................G..............A.**

**RCPE me h5**  **.......C...........................................................................G................**

**RCPE me h6**  **.......C...........................................................................G................**

**RCPE me h7**  **....................................................................................................**

**RCPE me h8**  **...................................................................................G.T..A...........**

**RCPE me h9**  **.......C...........................................................................G.G.T............**

**RCPE me h10**  **.......C...........................................................................G................**

**RCPE me h11**  **.......C...........................................................................G................**

**RCPE me h12**  **...................................................................................G....A...........**

**RCPE me h13**  **.......C...........................................................................G..............A.**

**RCPE me h14**  **.......C...........................................................................G................**

**RCPE me h15**  **.......C...........................................................................G................**

**RCPE me h16**  **.......C...........................................................................G................**

**RCPE me h17**  **.......C...........................................................................G................**

**RCPE me h18**  **.......C...........................................................................G................**

**RCPE me h19**  **.......C...........................................................................G................**

**RCPE me h20**  **.......C...........................................................................G................**

**RCPE si h1**  **...................................................T..................A............G....A..........G**

**RCPE si h2**  **...................................................T..................A............G....A..........G**

**RCPE si h3**  **...................................................T..................A............G....A..........G**

**RCPE si h4**  **...................................................T..................A............G....A..........G**

**RCPE si h5**  **...................................................T..................A............G....A..........G**

**RCPE si h6**  **...................................................T..................A............G....A..........G**

**RCPE si h7**  **...................................................T..................A............G....A..........G**

**RCPE si h8**  **...................................................T..................A............G....A..........G**

**RCPE si h9**  **...................................................G..................A............G....A..........G**

**RCPE si h10**  **...................................................T..................A............G....A..........G**

**RCPE si h11**  **...................................................G..................A...........GG....A..........G**

**RCPE si h12**  **...................................................T..................A............G....A..........G**

**RCPE si h13**  **...................................................T..................A............G....A..........G**

**RCPE se h1**  **...................................................T..................A.....A......G....A..........G**

**RCPE ma h1**  **....................................AA.............T..................A............G....A..........G**

**RCPE ya h1**  **........................A.......A...T....T.....C....A..........T......A...........CG...T.GA.........**

**RCPE ya h2**  **........................A.......A...T....T.....C....A..........T......A...........CG...T.GA.........**

**RCPE ya h3**  **........................A.......A...T....T.....C....A..........T......A...........CG...T.GA.........**

**RCPE ya h4**  **........................A.......A...T....T.....C....A..........T......A...........CG...T.GA.........**

**RCPE ya h5**  **........................A.......A...T....T.....C....A..........T......A...........CG...T.GA.........**

**RCPE ya h6**  **........................A.......A...T....T.....C....A..........T......A...........CG...T.GA.........**

**RCPE ya h7**  **........................A.......A...T....T.....C....A..........T......A...........CG...T.GA.........**

**RCPE ya h8**  **........................A..T....A...T....T.....C....A..........T......A...........CG...T.GA.........**

**RCPE ya h9**  **........................A.......A...T....T.....C....A..........T......A...........CG...T.GA.........**

**RCPE ya h10**  **...................A....A.......A...T....T.....C....A..........T......A...........CG...T.GA.........**

**RCPE ya h11**  **........................A.......A...T....T.....C....A..........T......A...........CG...T.GA.........**

**RCPE ya h12**  **........................A.......A...T....T.....C....A..........T......A...........CG...T.GA.........**

**RCPE ya h13**  **........................A.......A...T....T.....C....A..........T......A...........CG...T.GA.........**

**RCPE ya h14**  **...................A....A.......A...T....T.....C....A..........T......A...........CG...T.GA.........**

**RCPE ya h15**  **........................A.......A...T.A..T.....C....A..........T......A...........CG...T.GA.........**

**RCPE ya h16**  **........................A.......A...T....T.....C....A..........T......A...........CG...T.GA.........**

**RCPE te h1**  **........................A.......A...T....T..........A..............-..A............G...T.GA.........**

**RCPE te h2**  **........................A.......A...T....T..........A..............-..A..C.........G...T.GA.........**

**RCPE or h1**  **........................AA.............CGT..........A..G.GA.A........CA............G...TA.A..C......**

**RCPE er h1**  **......C.................A................T..........A....T.....G.....CA............G...TA....C.G....**

**Fixed substitutions**  **______1_________________11______1___21_111_____1___11__1_21_1__2___1_11___________1____1111__1_1___1**

**Polymorphic substitutions** **_______1___________1_______1__________1____________1_____________________1__1_____11_2_11_________1_**

**DNAse footprinting data** **____________PPPPPPPPPP____________________________________________________E_________________________**

1210 1220 1230 1240 1250 1260 1270 1280 1290 1300

....|....|....|....|....|....|....|....|....|....|....|....|....|....|....|....|....|....|....|....|

**RCPE me h1**  **~~GAAACTGAAGATTAC~~TTC~~ATTTA~~----~~TCTAGA~~CATCTGGCAAATTCAT~~CAAGA~~T~~GATTT~~CCCCAGCGCAGACTTAACGACTCGATCCGATCT~~GCGTG~~CA**

**RCPE me h2**  **.......................----.........................................................................**

**RCPE me h3**  **.......................----.........................................................................**

**RCPE me h4**  **.......................----.........................................................................**

**RCPE me h5**  **.......................----.........................................................................**

**RCPE me h6**  **.......................----.........................................................................**

**RCPE me h7**  **.......................----.........................................................................**

**RCPE me h8**  **.......................----.....................................C...................................**

**RCPE me h9**  **.......................----...C.....................................................................**

**RCPE me h10**  **.......................----...C.....................................................................**

**RCPE me h11**  **.......................----.........................................................................**

**RCPE me h12**  **.......................----.....................................C...................................**

**RCPE me h13**  **.......................----.........................................................................**

**RCPE me h14**  **....T..................----.........................................................................**

**RCPE me h15**  **.......................----.........................................................................**

**RCPE me h16**  **.......................----.........................................................................**

**RCPE me h17**  **.......................----.........................................................................**

**RCPE me h18**  **.......................----.........................................................................**

**RCPE me h19**  **.......................----.........................................................................**

**RCPE me h20**  **.......................----.....................................................................C...**

**RCPE si h1**  **.......................----.........................A...............................................**

**RCPE si h2**  **......A................----.........................A...............................................**

**RCPE si h3**  **.......................----.........................A...............................................**

**RCPE si h4**  **.......................----.........................A...............................................**

**RCPE si h5**  **.......................----.........................A...............................................**

**RCPE si h6**  **.......................CATA.........................A...............................................**

**RCPE si h7**  **......A................----.........................A...............................................**

**RCPE si h8**  **.......................----.........................A...............................................**

**RCPE si h9**  **.......................----.........................A...............................................**

**RCPE si h10**  **.......................----.........................A...............................................**

**RCPE si h11**  **.......................----.........................A...............................................**

**RCPE si h12**  **.......................----.........................A...............................................**

**RCPE si h13**  **.......................----.........................A...............................................**

**RCPE se h1**  **.......................----.........................A...............................................**

**RCPE ma h1**  **.......................----.........................A...............................................**

**RCPE ya h1**  **...G...................----.......................................................T.................**

**RCPE ya h2**  **...G...................----.....................A...................................................**

**RCPE ya h3**  **...G...................----.....................A...................................................**

**RCPE ya h4**  **...G...................----.........................................................................**

**RCPE ya h5**  **...G...................----.........................................................................**

**RCPE ya h6**  **...G...................----.........................................................................**

**RCPE ya h7**  **...G...................----.........................................................................**

**RCPE ya h8**  **...G...................----.........................................................................**

**RCPE ya h9**  **...G...................----.........................................................................**

**RCPE ya h10**  **...G...................----.........................................................................**

**RCPE ya h11**  **...G...................----.........................................................................**

**RCPE ya h12**  **...G...................----.........................................................................**

**RCPE ya h13**  **...G...................----.........................................................................**

**RCPE ya h14**  **...G...................----.........................................................................**

**RCPE ya h15**  **...G...................----.........................................................................**

**RCPE ya h16**  **...G...................----.........................................................................**

**RCPE te h1**  **...G.....A.A...........----..C......................................................................**

**RCPE te h2**  **...G.....A.A...........----..C....................T.................................................**

**RCPE or h1**  **.G.G...G...............----.............................G...........................G...............**

**RCPE er h1**  **...G...G...............----..............................................C................A.........**

**Fixed substitutions**  **_1_1___1_1_1_________________1__________________________1________________1__________1_____1_________**

**Polymorphic substitutions** **____1_1________________1______1_________________1_1_____________1_________________1_____________1___**

**DNAse footprinting data ____________________________________________________________________________________________________**

1310 1320 1330 1340 1350 1360 1370 1380 1390 1400

....|....|....|....|....|....|....|....|....|....|....|....|....|....|....|....|....|....|....|....|

**RCPE me h1**  **GT~~TTGAT~~TGT~~TAACT~~CAA~~ACAAA~~TCC~~AATAA~~CGCAAA~~AGAAA~~TTTTGAATAA~~TTTTC~~GAGCATCGCTA~~CTAATTAG~~CCGCCGATTGAGGCAATCAAATTG**

**RCPE me h2**  **..........................C...........................C.............................................**

**RCPE me h3**  **..........................C...........................C.............................................**

**RCPE me h4**  **....................................................................................................**

**RCPE me h5**  **..........................C...........................C.............................................**

**RCPE me h6**  **..........................C...........................C.............................................**

**RCPE me h7**  **....................................................................................................**

**RCPE me h8**  **..........................C...........................C.............................................**

**RCPE me h9**  **..........................C...........................C.............................................**

**RCPE me h10**  **..........................C...........................C.............................................**

**RCPE me h11**  **..........................C...........................C.............................................**

**RCPE me h12**  **..........................C...........................C.............................................**

**RCPE me h13**  **..........................C...........................C.............................................**

**RCPE me h14**  **..........................C...........................C.............................................**

**RCPE me h15**  **..........................C...........................C.............................................**

**RCPE me h16**  **..........................C...........................C.............................................**

**RCPE me h17**  **..........................C...........................C.............................................**

**RCPE me h18**  **..........................C...........................C.............................................**

**RCPE me h19**  **..........................C...........................C.............................................**

**RCPE me h20**  **..........................C...........................C.............................................**

**RCPE si h1**  **.......G..................C..........C................C.............................................**

**RCPE si h2**  **.......G..................C..........C................C.............................................**

**RCPE si h3**  **.......G..................C..........C................C.............................................**

**RCPE si h4**  **.......G..................C..........C................C.............................................**

**RCPE si h5**  **.......G..................C..........C................C.............................................**

**RCPE si h6**  **.......G..................C..........C................C.............................................**

**RCPE si h7**  **.......G..................C..........C................C.............................................**

**RCPE si h8**  **.......G..................C..........C................C.............................................**

**RCPE si h9**  **.......G..................C..........C................C.............................................**

**RCPE si h10**  **.......G..................C..........C................C.............................................**

**RCPE si h11**  **.......G..................C..........C................C.............................................**

**RCPE si h12**  **.......G..................C..........C................C.............................................**

**RCPE si h13**  **.......G..................C..........C................C.............................................**

**RCPE se h1**  **.......G..................C..........C................C............................................A**

**RCPE ma h1**  **.......G..................C..........C................C.............................................**

**RCPE ya h1**  **....A........A.G.....C..A.C.C.....C..C................C.............G......A........................**

**RCPE ya h2**  **....A...................A.C.C.G......C................C.........A...G......A........................**

**RCPE ya h3**  **....A...................A.C.C.G......C................C.........A...G......A........................**

**RCPE ya h4**  **....A...................A.C.C........C................C.............G......A........................**

**RCPE ya h5**  **....A...................A.C.C........C................C....................A........................**

**RCPE ya h6**  **....A...................A.C.C.G......C................C.........A...G......A........................**

**RCPE ya h7**  **....A...................A.C.C........C................C.............G......A........................**

**RCPE ya h8**  **....A........A..........A.C.C.G......C................C.........A...G......A........................**

**RCPE ya h9**  **....A...................A.C.C........C................C.............G......A........................**

**RCPE ya h10**  **....A...................A.C.C.G......C................C.........A...G......A........................**

**RCPE ya h11**  **....A...................A.C.C........C................C.............G......A........................**

**RCPE ya h12**  **....A...................A.C.C........C................C.............G......A........................**

**RCPE ya h13**  **....A..................CA.C.C.G......C................C.............G......A........................**

**RCPE ya h14**  **....A........A.......C..A.C.C.....C..C................C.............G......A........................**

**RCPE ya h15**  **....A........A.......C..A.C.C.....C..C................C.............G......A........................**

**RCPE ya h16**  **....A...................A.C.C........C................C.............G......A........................**

**RCPE te h1**  **....A...................A.C.CG....C..C................C.............G......A........................**

**RCPE te h2**  **....A...................A.C.GG....C..C................C.............G......A........................**

**RCPE or h1**  **.........C................C..........C................C....................A........................**

**RCPE er h1**  **..........................C..........C................C....................A........................**

**Fixed substitutions**  **____1__1_1_____________1___11_______1_____________________________________1_________________________**

**Polymorphic substitutions** **_____________1_1_____1_1__1_1_1___1^2^__________________1_________1___1^2^_______________________________**

**DNAse footprinting data ____________________________________________________________________________________________________**

1410 1420 1430 1440 1450 1460 1470 1480 1490 1500

....|....|....|....|....|....|....|....|....|....|....|....|....|....|....|....|....|....|....|....|

**RCPE me h1**  **ATTGCCTGCGATCCGAACCAA~~AGAAAACC~~T~~CACGC~~TGGGATCCCCG~~AACCC~~A~~CCATA~~GACCGATTGACGCTGATACT~~ATATATA~~GACGGTG---TAT~~CGG~~**

**RCPE me h2**  **...........................................................................................---......**

**RCPE me h3**  **...........................................................................................---......**

**RCPE me h4**  **...........................................................................................---......**

**RCPE me h5**  **...........................................................................................---......**

**RCPE me h6**  **...........................................................................................---......**

**RCPE me h7**  **...........................................................................................---......**

**RCPE me h8**  **...........................................................................................---......**

**RCPE me h9**  **...........................................................................................---......**

**RCPE me h10**  **...........................................................................................---......**

**RCPE me h11**  **...........................................................................................---......**

**RCPE me h12**  **...........................................................................................---......**

**RCPE me h13**  **...........................................................................................---......**

**RCPE me h14**  **...........................................................................................---......**

**RCPE me h15**  **...........................................................................................---......**

**RCPE me h16**  **...........................................................................................---......**

**RCPE me h17**  **...........................................................................................---......**

**RCPE me h18**  **...........................................................................................---......**

**RCPE me h19**  **...........................................................................................---......**

**RCPE me h20**  **..................................................................................C........---......**

**RCPE si h1**  **......................................................T....................................---......**

**RCPE si h2**  **......................................................T....................................---......**

**RCPE si h3**  **......................................................T....................................---......**

**RCPE si h4**  **......................................................T....................................---......**

**RCPE si h5**  **......................................................T....................................---......**

**RCPE si h6**  **......................................................T....................................---......**

**RCPE si h7**  **......................................................T....................................---......**

**RCPE si h8**  **......................................................T....................................---......**

**RCPE si h9**  **......................................................T....................................---......**

**RCPE si h10**  **......................................................T....................................---......**

**RCPE si h11**  **......................................................T....................................---......**

**RCPE si h12**  **......................................................T....................................---......**

**RCPE si h13**  **......................................................T....................................---......**

**RCPE se h1**  **......................................................T....................................---......**

**RCPE ma h1**  **......................................................T....................................---......**

**RCPE ya h1**  **................G..........G.........................AT.........C..........................---......**

**RCPE ya h2**  **................G..........G.........................AT.........C..........................---......**

**RCPE ya h3**  **................G..........G.........................AT.........C..........................---......**

**RCPE ya h4**  **................G..........G.........................AT.........C..........................---......**

**RCPE ya h5**  **................G..........G.........................AT.........C..........................---......**

**RCPE ya h6**  **................G..........G.........................AT.........C..........................---......**

**RCPE ya h7**  **................G..........G.........................AT.........C..........................---......**

**RCPE ya h8**  **................G..........G.........................AT.........C..........................---......**

**RCPE ya h9**  **................G..........G.........................AT.........C..........................---......**

**RCPE ya h10**  **................G....-.....G.........................AT.........C..........................---......**

**RCPE ya h11**  **................G..........G.........................AT.........C..........................---......**

**RCPE ya h12**  **................G..........G.........................AT.........C..........................---......**

**RCPE ya h13**  **................G..........G.........................AT.........C..........................---......**

**RCPE ya h14**  **................G..........G.........................AT.........C..........................---......**

**RCPE ya h15**  **................G..........G.........................AT.........C..........................---......**

**RCPE ya h16**  **................G..........G.........................AT.........C..........................---......**

**RCPE te h1**  **................G....C.....G.........................AT.....T...C..........................---......**

**RCPE te h2**  **................G....C.....G.........................AT.....T...C..........................---......**

**RCPE or h1**  **..............A.G..G...T...................G..........T....................................GTG......**

**RCPE er h1**  **..............A.G......T...................G..........T....................................---......**

**Fixed substitutions**  **______________1_1__1_1_1___1_______________1_________11_____1___1__________________________1________**

**Polymorphic substitutions** **__________________________________________________________________________________1_________________**

**DNAse footprinting data ____________________________________________________________________________________________________**

1510 1520 1530 1540 1550 1560 1570 1580 1590 1600

....|....|....|....|....|....|....|....|....|....|....|....|....|....|....|....|....|....|....|....|

**RCPE me h1**  **~~AATAG~~T-CGGGGA~~ATTTA~~CACCAATCTCGAG~~TT-~~G~~TTTGTTTTGTTTGT~~TGATAA~~TTTTTCT~~CGG~~CCCTTT~~A~~CTATTTTAT~~TCCCA~~GCGGG~~TCGTC~~GCCT~~**

**RCPE me h2**  **......-.......C..................-..................................................................**

**RCPE me h3**  **......-..........................-..................................................................**

**RCPE me h4**  **......-..........................-..................................................................**

**RCPE me h5**  **......-..........................-..................................................................**

**RCPE me h6**  **......-..........................-..................................................................**

**RCPE me h7**  **......-..........................-..................................................................**

**RCPE me h8**  **......-..........................-.........................................................C........**

**RCPE me h9**  **......-..........................-..................................................................**

**RCPE me h10**  **......-..........................-..................................................................**

**RCPE me h11**  **......-.......C..................-..................................................................**

**RCPE me h12**  **......-..........................-.........................................................C........**

**RCPE me h13**  **......-..........................-..................................................................**

**RCPE me h14**  **......-.......C..................-..................................................................**

**RCPE me h15**  **......-.......C..................-..................................................................**

**RCPE me h16**  **......-.......C..................-..................................................................**

**RCPE me h17**  **......-..........................-..................................................................**

**RCPE me h18**  **......-..........................-..................................................................**

**RCPE me h19**  **......-.......C..................-..................................................................**

**RCPE me h20**  **......-..........................-..................................................................**

**RCPE si h1**  **......-..........................T..................................................................**

**RCPE si h2**  **......-..........................T..................................................................**

**RCPE si h3**  **......-..........................T..................................................................**

**RCPE si h4**  **......-..........................T..................................................................**

**RCPE si h5**  **......-..........................T..................................................................**

**RCPE si h6**  **......-..........................T..................................................................**

**RCPE si h7**  **......-..........................T..................................................................**

**RCPE si h8**  **......-..........................T..................................................................**

**RCPE si h9**  **......-.....................A....T..................................................................**

**RCPE si h10**  **......-..........................T..................................................................**

**RCPE si h11**  **......-..........................T..............................A...................................**

**RCPE si h12**  **......-..........................T..................................................................**

**RCPE si h13**  **......-.....................A....T..................................................................**

**RCPE se h1**  **......-..........................T..................................................................**

**RCPE ma h1**  **......-..........................T..................................................................**

**RCPE ya h1**  **......A..........................-.............................A.........................T.G........**

**RCPE ya h2**  **......A..........................-.............................A.........................T.G........**

**RCPE ya h3**  **......A..........................-.............................A.........................T.G........**

**RCPE ya h4**  **......A..........................-.............................A.........................T.G........**

**RCPE ya h5**  **......A..........................-.............................A.........................T.G........**

**RCPE ya h6**  **......A..........................-.............................A.........................T.G........**

**RCPE ya h7**  **......A..........................-.............................A.........................T.G........**

**RCPE ya h8**  **......A..........................-.............................A.........................T.G........**

**RCPE ya h9**  **......A..........................-.............................A.........................T.G........**

**RCPE ya h10**  **......A..........................-.............................A.........................T.G........**

**RCPE ya h11**  **......A..........................-.............................A.........................T.G........**

**RCPE ya h12**  **......A..........................-.............................A.........................T.G........**

**RCPE ya h13**  **......A..........................-.............................A.........................T.G........**

**RCPE ya h14**  **......A..........................-.............................A.........................T.G........**

**RCPE ya h15**  **......A..........................-.............................A.........................T.G........**

**RCPE ya h16**  **......A..........................-.............................A..............C..........T.G........**

**RCPE te h1**  **......A..........................-.............................A..........G.........................**

**RCPE te h2**  **......A..........................-.............................A..........G.........................**

**RCPE or h1**  **......AT.........................-...............................................A................G.**

**RCPE er h1**  **......AT.........................-.................................-.............A................A.**

**Fixed substitutions**  **______11_________________________1_____________________________1___1______1______1_______1_1______2_**

**Polymorphic substitutions** **______________1_____________1___________________________________1_____________1____________1________**

**DNAse footprinting data ____________________________________________________________________________________________________**

1610 1620 1630 1640 1650 1660 1670 1680 1690 1700

....|....|....|....|....|....|....|....|....|....|....|....|....|....|....|....|....|....|....|....|

**RCPE me h1**  **~~GTT~~T~~CACTG~~TGA~~TAATGAGTGTGATATACTC~~GATTG~~TTTTTATCACCCC~~TTC~~CCCAATTAC~~GTGGTATTT~~GGTATATAATGAGAAAA~~--~~CC~~G~~CCGGA~~GGA**

**RCPE me h2**  **.......................................................................................--...........**

**RCPE me h3**  **............................................................T..........................--...........**

**RCPE me h4**  **.......................................................................................--...........**

**RCPE me h5**  **.......................................................................................--...........**

**RCPE me h6**  **............................................................T..........................--...........**

**RCPE me h7**  **............................................................T..........................--...........**

**RCPE me h8**  **.......................................................................................--...........**

**RCPE me h9**  **.......................................................................................--...........**

**RCPE me h10**  **.......................................................................................--...........**

**RCPE me h11**  **.......................................................................................--...........**

**RCPE me h12**  **.......................................................................................--...........**

**RCPE me h13**  **............................................................T..........................--...........**

**RCPE me h14**  **...............................C.......................................................--...........**

**RCPE me h15**  **........................................................G..............................--...........**

**RCPE me h16**  **............................................................T..........................--...........**

**RCPE me h17**  **............................................................T..........................--...........**

**RCPE me h18**  **.......................................................................................--...........**

**RCPE me h19**  **.......................................................................................--...........**

**RCPE me h20**  **.......................................................................................--...........**

**RCPE si h1**  **........C.................C.............................T............C.................--...........**

**RCPE si h2**  **........C.A.............................................T............C.................--...........**

**RCPE si h3**  **........C...............................................T....A.......C.................--...........**

**RCPE si h4**  **........C...............................................T....A.......C.................--...........**

**RCPE si h5**  **.....C..C.................C.............................T....A.......C.................--...........**

**RCPE si h6**  **........C...............................................T............C.................--...........**

**RCPE si h7**  **.....C..C.................C.............................T............C.................--...........**

**RCPE si h8**  **........C...............................................T....A.......C.................--...........**

**RCPE si h9**  **........C...............................................T............C.................--...........**

**RCPE si h10**  **........C...............................................T............C.................--...........**

**RCPE si h11**  **.....C..C.................C.............................T............C.................--...........**

**RCPE si h12**  **.....C..C.................C.............................T............C.................--...........**

**RCPE si h13**  **........C...............................................T............C.................--...........**

**RCPE se h1**  **........C..........A....................................T............C.................--...........**

**RCPE ma h1**  **........C...............................................T...A........C.................--...........**

**RCPE ya h1**  **.C......C............C..................................T...T............T.G...........TC..C.TA...A.**

**RCPE ya h2**  **.C......C............C..................................T...T............T.G...........TC..C.TA...A.**

**RCPE ya h3**  **.C......C............C..................................T...T............T.G...........TC..C.TA...A.**

**RCPE ya h4**  **.C......C............C........................T.........T...T............T.G...........TC..C.TA...A.**

**RCPE ya h5**  **.C......C............C..................................T...T............T.G...........TC..C.TA...A.**

**RCPE ya h6**  **.C......C............C..................................T...T............T.G...........TC..C.TA...A.**

**RCPE ya h7**  **.C......C............C..................................T...T............T.G...........TC..C.TA...A.**

**RCPE ya h8**  **.C......C............C..................................T...T............T.G...........TC..C.TA...A.**

**RCPE ya h9**  **.C......C............C..................................T...T............T.G...........TC..C.TA...A.**

**RCPE ya h10**  **.C......C............C..................................T...T............T.G...........TC..C.TA...A.**

**RCPE ya h11**  **.C......C............C..................................T...T............T.G...........TC..C.TA...A.**

**RCPE ya h12**  **.C......C............C..................................T...T............T.G...........TC..C.TA...A.**

**RCPE ya h13**  **.C......C............C..................................T...T............T.G...........TC..C.TA...A.**

**RCPE ya h14**  **.C......C............C..................................T...T............T.G...........TC..C.TA...A.**

**RCPE ya h15**  **.C......C............C..................................T...T............T.G...........TC..C.TA...A.**

**RCPE ya h16**  **.C......C............C..................................T...T............T.G...........TC..C.TA...A.**

**RCPE te h1**  **.C......C............C......G...........................T...T............T.G...........TC..C..A...A.**

**RCPE te h2**  **.C......C............C......G...........................T...T............T.G...........TC..C..A...A.**

**RCPE or h1**  **.G......C............C.......G....................G.....T.C.T....C...................G.GC..C.T......**

**RCPE er h1**  **.G......C............C..................................C.CTT....C.....................GC..C.A......**

**Fixed substitutions**  **_2______1__________1_1______11____________________1_____2_112____1___1___1_1_________1_2___1_2^1^___1_**

**Polymorphic substitutions** **_____1____1_______________1____1______________1_________1___11______________________________________**

**DNAse footprinting data ____________________________________________________________________________________________________**

**A**

10 20 30 40 50 60 70 80 90 100

....|....|....|....|....|....|....|....|....|....|....|....|....|....|....|....|....|....|....|....|

**ZE me h1**  **CCACGAGGGCAA~~ACAAAAA~~GCGCAAA~~CACGC~~GACCCTCGGC~~CACGC~~GTAT~~TCCTG~~ATCC~~CAGG~~G~~A~~TCGGAC~~GTAATG~~TTATCCTTTG~~GCCGC~~CCAGTGCC**

**ZE me h2**  **....................................................................................................**

**ZE me h3**  **....................................................................................................**

**ZE me h4**  **....................................................................................................**

**ZE si h1**  **.................................................G.....G............................................**

**ZE si h2**  **.................................................G.....G............................................**

**ZE si h3**  **.................................................G.....G............................................**

**ZE si h4**  **.................................................G.....G............................................**

**ZE si h5**  **.................................................G.....G............................................**

**ZE si h6**  **.................................................G.....G............................................**

**ZE si h7**  **.................................................G.....G............................................**

**ZE si h8**  **.................................................G.....G............................................**

**ZE si h9**  **.................................................G.....G............................................**

**ZE si h10**  **.................................................G.....G............................................**

**ZE se h1**  **.................................................G.....G............................................**

**ZE ma h1**  **.................................................G.....G............................................**

**ZE ya h1**  **.................................................G.....G.......-.............................-......**

**ZE ya h2**  **.................................................G.....G.......-.............................-......**

**ZE ya h3**  **.................................................G.....G.......-.............................-......**

**ZE ya h4**  **.................................................G.....G.......-.............................-......**

**ZE ya h5**  **.................................................G.....G.......-.............................-......**

**ZE ya h6**  **.................................................G.....G.......-.............................-......**

**ZE ya h7**  **.................................................G.....G.......-.............................-......**

**ZE ya h8**  **.................................................G.....G.......-.............................-......**

**ZE ya h9**  **.................................................G.....G.......-.............................-......**

**ZE te h1**  **.................................................G.....G............................................**

**ZE te h2**  **.................................................G.....G............................................**

**ZE or h1**  **.................................................G.....G.......-........C...........................**

**ZE er h1**  **.................................................G.....G.....................................-......**

**Fixed substitutions**  **_________________________________________________1_____1_______1^1^_______1____________________1^1^______**

**Polymorphic substitutions** **____________________________________________________________________________________________________**

**DNAse footprinting data** **____________________________________________________________________________________________________**

110 120 130 140 150 160 170 180 190 200

....|....|....|....|....|....|....|....|....|....|....|....|....|....|....|....|....|....|....|....|

**ZE me h1**  **ACGAA~~ATAAAT~~TCGGAG~~GGAAA~~GGGCATC~~GGGTTCCG~~GGAACAACTGGCAGCCAGTCTTCGGTGTTTTGCGCGCTGGC~~AAAAA~~TCCAG~~AGAAATTTTT~~AG**

**ZE me h2**  **....................................................................................................**

**ZE me h3**  **........T...........................................................................................**

**ZE me h4**  **....................................................................................................**

**ZE si h1**  **G..........................G...A..................A.................................................**

**ZE si h2**  **G..........................G...A..................A.................................................**

**ZE si h3**  **G..........................G...A..................A.................................................**

**ZE si h4**  **G..........................G...A..................A.................................................**

**ZE si h5**  **G..........................G...A..................A.................................................**

**ZE si h6**  **G..........................G...A..................A.................................................**

**ZE si h7**  **G..........................G...A..................A.................................................**

**ZE si h8**  **G..........................G...A..................A.................................................**

**ZE si h9**  **G..........................G...A..................A.................................................**

**ZE si h10**  **G..........................G...A..................A.................................A...............**

**ZE se h1**  **G..........................G...A..................A.................................................**

**ZE ma h1**  **G..........................G...A..................A....----------------.............................**

**ZE ya h1**  **.......G.......C...........A...A...............C.........C...............................T..........**

**ZE ya h2**  **.......G.......C...........A...A...............C.........C...............................T..........**

**ZE ya h3**  **.......G.......C...........A...A...............C.........C...............................T..........**

**ZE ya h4**  **.......G.......C...........A...A...............C.........C...............................T..........**

**ZE ya h5**  **.......G.......C...........A...A...............C.........C...............................T..........**

**ZE ya h6**  **.......G.......C...........A...A...............C.........C...............................T..........**

**ZE ya h7**  **.......G.......C...........A...A...............C..C......C...............................T..........**

**ZE ya h8**  **.......G.......C...........A...A...............C..C......C...............................T..........**

**ZE ya h9**  **.T.....G.......C...........A...A...............C.........C...............................T..........**

**ZE te h1**  **...............C...........G...A...............C.........C...............................T.C........**

**ZE te h2**  **...............C...........G...A...............C.........C...............................T.C........**

**ZE or h1**  **.......G...................G...A.........................C...............................T..........**

**ZE er h1**  **G..........................G...A.........................C...............................T..........**

**Fixed substitutions**  **1^1^_____1^1^_______1___________2___1_______________1__1____1_1­_______________________________1_1________**

**Polymorphic substitutions** **_1______1_________________________________________1_________________________________1_______________**

**DNAse footprinting data ____________________________________________________________________________________________________**

210 220 230 240 250 260 270 280 290 300

....|....|....|....|....|....|....|....|....|....|....|....|....|....|....|....|....|....|....|....|

**ZE me h1**  **GGAACC~~ATAAA~~CGGGCCGG~~GGAAAAAG~~CCTCTGCGCCGA~~AGGAA~~CG~~TTTTC~~AGC~~AACAG~~TTTAC~~A~~---------------------~~GTTTTTAT~~G~~TCTTT~~**

**ZE me h2**  **..................................C..............................---------------------..............**

**ZE me h3**  **.................................................................---------------------..............**

**ZE me h4**  **..........................T......................................---------------------..............**

**ZE si h1**  **....................-............................................---------------------..............**

**ZE si h2**  **.................................................................---------------------..............**

**ZE si h3**  **....................-............................................---------------------..............**

**ZE si h4**  **.................................................................---------------------..............**

**ZE si h5**  **....................-............................................---------------------..............**

**ZE si h6**  **.................................................................---------------------..............**

**ZE si h7**  **.................................T...............................---------------------..............**

**ZE si h8**  **.................................................................---------------------..............**

**ZE si h9**  **.................................................................---------------------..............**

**ZE si h10**  **.................................................................---------------------..............**

**ZE se h1**  **.................A...............................................---------------------..............**

**ZE ma h1**  **.................................................................---------------------..............**

**ZE ya h1**  **...............T........C................A..........T............GTTTACAG-------TATACA..............**

**ZE ya h2**  **...............T........C................A..........T............GTTTACAG-------TATACA..............**

**ZE ya h3**  **...............T........C................A..........T............GTTTACAGTTTACAGTATACA..............**

**ZE ya h4**  **...............T........C................A..........T............GTTTACAG-------TATACA..............**

**ZE ya h5**  **...............T........C................A..........T............GTTTACAG-------TATACA..............**

**ZE ya h6**  **...............T........C................A..........T............GTTTACAG-------TTTACA..............**

**ZE ya h7**  **...............T........C................A..........T............GTTTACAG-------TATACA..............**

**ZE ya h8**  **...............T........C................A..........T............GTTTACAGTTTACAGTATACA..............**

**ZE ya h9**  **...............T........C................A..........T............GTTTACAG-------TATACA..............**

**ZE te h1**  **...............T........T................A..........T............GTATACAG-------TATACA..............**

**ZE te h2**  **...............T........T................A..........T............-~~TATA~~CAG-------TATACA..............**

**ZE or h1**  **...............T.................................................GTTTAC-~~G~~-------------..........T...**

**ZE er h1**  **...............T.................................................---------------------..........T...**

**Fixed substitutions**  **_______________1_1______2________________1__________1____________1^1^1___1________1_______________1___**

**Polymorphic substitutions** **____________________1_____1______11______________________________1_______1_______1__________________**

**DNAse footprinting data ____________________________________________________________________________________________________**

310 320 330 340 350 360 370 380 390 400

....|....|....|....|....|....|....|....|....|....|....|....|....|....|....|....|....|....|....|....|

**ZE me h1**  **~~ATGATT~~ATTGCA~~ATTAGAGG~~GAGA----------TCGGCTGAGAGTCGCGCCCTCTCGCTCTGCGCACCTCATAGGTAGGCACCTCATGGCC~~GTAATTAC~~**

**ZE me h2**  **........................----------..................................................................**

**ZE me h3**  **........................----------..................................................................**

**ZE me h4**  **........................----------..................................................................**

**ZE si h1**  **........................----------...............--.................................................**

**ZE si h2**  **........................----------...............--.................................................**

**ZE si h3**  **........................----------...............--.................................................**

**ZE si h4**  **........................----------...............--.................................................**

**ZE si h5**  **........................----------...............--.................................................**

**ZE si h6**  **........................----------...............--.................................................**

**ZE si h7**  **........................----------...............--.................................................**

**ZE si h8**  **........................----------..................................................................**

**ZE si h9**  **........................----------..................................................................**

**ZE si h10**  **........................----------...............--.................................................**

**ZE se h1**  **........................----------...............--.................A...............................**

**ZE ma h1**  **........................----------..................................................................**

**ZE ya h1**  **...................A....GAGAGAGAGA..................................................................**

**ZE ya h2**  **...................A....GAGGGAGAGA..................................................................**

**ZE ya h3**  **...................A....GAGAGAGAGA..................................................................**

**ZE ya h4**  **...................A....GAGAGAGA--..A...............................................................**

**ZE ya h5**  **...................A....GAGAGAGA--..................................................................**

**ZE ya h6**  **...................A....GAGGGAGAGA..................................................................**

**ZE ya h7**  **...................A....GAGAGAGAGA..................................................................**

**ZE ya h8**  **...................A....GAGAGAGAGA..................................................................**

**ZE ya h9**  **...................A....GAGAGAGAGA..................................................................**

**ZE te h1**  **................A.AA....----------.A................................................................**

**ZE te h2**  **................A.AA....----------.A................................................................**

**ZE or h1**  **........................----------..................A........C.T............C.......................**

**ZE er h1**  **........................----------.....C............A..........T....................................**

**Fixed substitutions**  **________________1_11____1__________1___1____________1________1_1____1_______1_______________________**

**Polymorphic substitutions** **___________________________1____1___1____________1__________________________________________________**

**DNAse footprinting data ____________________________________________________________________________________________________**

410 420 430 440 450 460 470 480 490 500

....|....|....|....|....|....|....|....|....|....|....|....|....|....|....|....|....|....|....|....|

**ZE me h1**  **TGCAGCACCGTCTCAAGGTCGCCGAGTAGGAGAAGCGCGCGGGCGG~~ATAAAT~~CGCGATGA~~TAATG~~GGCGCGATGGGTAG~~GTAAT~~AAGC~~CGCGC~~AG~~CAGGT~~**

**ZE me h2**  **....................................................................................................**

**ZE me h3**  **....................................................................................................**

**ZE me h4**  **....................................................................................................**

**ZE si h1**  **.......G..........C................T................................................................**

**ZE si h2**  **.......G..........C................T................................................................**

**ZE si h3**  **.......G..........C................T................................................................**

**ZE si h4**  **.......G..........C................T................................................................**

**ZE si h5**  **.......G..........C................T................................................................**

**ZE si h6**  **.......G..........C................T................................................................**

**ZE si h7**  **.......G..........C................T................................................................**

**ZE si h8**  **.......G..........C................T................................................................**

**ZE si h9**  **.......G..........C................T................................................................**

**ZE si h10**  **.......G..........C................T................................................................**

**ZE se h1**  **.......GG.........A................T................................................................**

**ZE ma h1**  **.......G.C.........................T................................................................**

**ZE ya h1**  **.......G..................G........T.......T......G......................................A..........**

**ZE ya h2**  **.......G..................G........T.......T......G......................................A..........**

**ZE ya h3**  **.......G..................G........T.......T......G......................................A..........**

**ZE ya h4**  **.......G..................G........T.......T......G......................................A..........**

**ZE ya h5**  **.......G..................G........T.......T......G......................................A..........**

**ZE ya h6**  **.......G..................G........T.......T......G......................................A..........**

**ZE ya h7**  **.......G..................G........T.......T......G......................................A..........**

**ZE ya h8**  **.......G..................G........T.......T......G......................................A..........**

**ZE ya h9**  **.......G..................G........T.......T......G......................................A..........**

**ZE te h1**  **.......G..................G........T.......T......G......................................A..........**

**ZE te h2**  **.......G..................G........T.......T......G......................................A..........**

**ZE or h1**  **.......G..................G........T.......T...........................................T............**

**ZE er h1**  **.......G..................G........T.......T...........................................T............**

**Fixed substitutions**  **_______111________2_______1________1_______1______1____________________________________1_1__________**

**Polymorphic substitutions** **____________________________________________________________________________________________________**

**DNAse footprinting data ____S__________________PPPPP__________PPPPPPPPPPPPPPPPPPPPPPPPPPPPPP________________________________**

510 520 530 540 550 560 570 580 590 600

....|....|....|....|....|....|....|....|....|....|....|....|....|....|....|....|....|....|....|....|

**ZE me h1**  **AGGCACCGTACGGATAAAGTTGC~~CAGGA~~CCTCGGATAACTTCCCCTCTCCGTGCCTGCAAGGACA~~TTTCG~~CCGGA~~GGGGT~~GGCTGCG~~AACAG~~CAGGCGGC**

**ZE me h2**  **....................................................................................................**

**ZE me h3**  **....................................................................................................**

**ZE me h4**  **....................................................................................................**

**ZE si h1**  **....T................................................................A..............................**

**ZE si h2**  **....T................................................................A..............................**

**ZE si h3**  **....T................................................................A..............................**

**ZE si h4**  **....T................................................................A..............................**

**ZE si h5**  **....C..............................................................-................................**

**ZE si h6**  **....T................................................................A..............................**

**ZE si h7**  **....C..............................................................-................................**

**ZE si h8**  **....C..............................................................-................................**

**ZE si h9**  **....C..............................................................-................................**

**ZE si h10**  **....T................................................................A..............................**

**ZE se h1**  **....T................................................................A..............................**

**ZE ma h1**  **....T............GC................................................-................................**

**ZE ya h1**  **....T.........................................................C......C..A...........................**

**ZE ya h2**  **....T.........................................................C......C..A...........................**

**ZE ya h3**  **....T.........................................................C......C..A...........................**

**ZE ya h4**  **....T.........................................................C......C..A...........................**

**ZE ya h5**  **....T.........................................................C......C..A...........................**

**ZE ya h6**  **....T.........................................................C......C..A...........................**

**ZE ya h7**  **....T.........................................................C......C..A...........................**

**ZE ya h8**  **....T.........................................................C......C..A...........................**

**ZE ya h9**  **....T.........................................................C......C..A...........................**

**ZE te h1**  **....C.................................T.......................C......C..A...........................**

**ZE te h2**  **....C.................................T.......................C......C..A...........................**

**ZE or h1**  **....C...............G...........................................G....C..............................**

**ZE er h1**  **....C...............G.................T.........................G....C..............................**

**Fixed substitutions**  **____2____________11_1_________________1^1^______________________1_1____1__1___________________________**

**Polymorphic substitutions** **____1______________________________________________________________1_1______________________________**

**DNAse footprinting data ______PPPPPPPPPPPPPPPPPPPPPPPPPPPPPPPPPPPP__________PPPPPPPPPPPPPPPPPPPPP___________________________**

610 620 630 640 650 660 670 680 690 700

....|....|....|....|....|....|....|....|....|....|....|....|....|....|....|....|....|....|....|....|

**ZE me h1**  **AAAGTGTCATGCGCAGGGAT~~ATTTAT~~GCGCTAT~~AACGG~~CGA~~GCGTG~~TGCCGAGGGCTCTCTGATTTTGC~~TATATAT~~G~~CAGGA~~TCT~~GCCGCAGGA~~CCAGCT**

**ZE me h2**  **....................................................................................................**

**ZE me h3**  **....................................................................................................**

**ZE me h4**  **....................................................................................................**

**ZE si h1**  **....................................................................................................**

**ZE si h2**  **....................................................................................................**

**ZE si h3**  **....................................................................................................**

**ZE si h4**  **........................................................................................T...........**

**ZE si h5**  **....................................................................................................**

**ZE si h6**  **........................................................................................T...........**

**ZE si h7**  **....................................................................................................**

**ZE si h8**  **....................................................................................................**

**ZE si h9**  **........................................................................................T...........**

**ZE si h10**  **........................................................................................T...........**

**ZE se h1**  **....................................................................................................**

**ZE ma h1**  **........................................................................................T...........**

**ZE ya h1**  **.....................................................A..C.............................T............A**

**ZE ya h2**  **.....................................................A..C.............................T............A**

**ZE ya h3**  **.....................................................A..C.............................T............A**

**ZE ya h4**  **.....................................................A..C.............................T............A**

**ZE ya h5**  **.....................................................A..C.............................T............A**

**ZE ya h6**  **.....................................................A..C.............................T............A**

**ZE ya h7**  **.....................................................A..C.............................T............A**

**ZE ya h8**  **.....................................................A..C.............................T............A**

**ZE ya h9**  **.....................................................A..C.............................T............A**

**ZE te h1**  **....................................A................A......A.........................T............A**

**ZE te h2**  **....................................A................A......A.........................T............A**

**ZE or h1**  **...........................................................G.....C....................T.............**

**ZE er h1**  **.....................................................A.....G..........................T.............**

**Fixed substitutions**  **____________________________________1________________1^1^_1__11____1____________________1____________1**

**Polymorphic substitutions** **________________________________________________________________________________________1^2^__________**

**DNAse footprinting data ______________PPPPPPPPPPPPPPP__________________PPPPPPPPPPPPPPPPPPPPP________________________________**

710 720 730 740 750 760 770 780 790 800

***ftz***

....|....|....|....|....|....|....|....|....|....|....|....|....|....|....|....|....|....|....|....|

**ZE me h1**  **CATTCGCAAACTCACCAGCGTT~~GCGTG~~CACATCGCAGAGTTAGAGA~~AGAAA~~TCTAG~~CAATAC~~*ACATCC*GATATGGCCACCACA~~AACAG~~CCAGAGCCACTA**

**ZE me h2**  **....................................................................................................**

**ZE me h3**  **....................................................................................................**

**ZE me h4**  **....................................................................................................**

**ZE si h1**  **.......................T............................................................................**

**ZE si h2**  **....................................................................................................**

**ZE si h3**  **...............A....................................................................................**

**ZE si h4**  **...............A....................................................................................**

**ZE si h5**  **.......................T............................................................................**

**ZE si h6**  **....................................................................................................**

**ZE si h7**  **....................................................................................................**

**ZE si h8**  **...............A...C................................................................................**

**ZE si h9**  **...............A...C................................................................................**

**ZE si h10**  **...............A....................................................................................**

**ZE se h1**  **....................................................................................................**

**ZE ma h1**  **...............A..........................................T.........................................**

**ZE ya h1**  **................................................C.....................G.............................**

**ZE ya h2**  **................................................C.............G....T..G.............................**

**ZE ya h3**  **................................................C.....................G.............................**

**ZE ya h4**  **................................................C.............G....T..G.............................**

**ZE ya h5**  **................................................C.....................G.............................**

**ZE ya h6**  **................................................C.....................G.............................**

**ZE ya h7**  **................................................C.....................G.............................**

**ZE ya h8**  **................................................C.....................G.............................**

**ZE ya h9**  **................................................C.....................G.............................**

**ZE te h1**  **.......................................A........C.....................G.............................**

**ZE te h2**  **.......................................A........C.....................G.............................**

**ZE or h1**  **...................................C..................................G...........G.................**

**ZE er h1**  **...............................................C..T........C..........G.............................**

**Fixed substitutions**  **___________________________________1___1_______11_1_______11__________1___________1_________________**

**Polymorphic substitutions** **_______________1^2^__1___1______________________________________1____1________________________________**

**DNAse footprinting data ___________________________________________________________________E________________________________**

810 820 830

....|....|....|....|....|....|....

**ZE me h1**  **CAGCTACGCCGACAACATGAACATGTACAACATG**

**ZE me h2**  **..................................**

**ZE me h3**  **..................................**

**ZE me h4**  **..................................**

**ZE si h1**  **..................................**

**ZE si h2**  **..................................**

**ZE si h3**  **..................................**

**ZE si h4**  **..................................**

**ZE si h5**  **..................................**

**ZE si h6**  **..................................**

**ZE si h7**  **..................................**

**ZE si h8**  **..................................**

**ZE si h9**  **..................................**

**ZE si h10**  **..................................**

**ZE se h1**  **..................................**

**ZE ma h1**  **..................................**

**ZE ya h1**  **..................................**

**ZE ya h2**  **..................................**

**ZE ya h3**  **..................................**

**ZE ya h4**  **..................................**

**ZE ya h5**  **..................................**

**ZE ya h6**  **..................................**

**ZE ya h7**  **..................................**

**ZE ya h8**  **..................................**

**ZE ya h9**  **..................................**

**ZE te h1**  **..................................**

**ZE te h2**  **..................................**

**ZE or h1**  **..................................**

**ZE er h1**  **..................................**

**Fixed substitutions**  **__________________________________**

**Polymorphic substitutions** **__________________________________**

**DNAse footprinting data __________________________________**

**B**

**Figure S1.** **Haplotypes and TFBSs of the RCPE (A) and ZE (B) from the eight *Drosophila* species studied in this work.**

Shaded positions are those indentified by PATCH searches as 5 bp minimum sequences that are perfect matches to transcription factor binding sequences at the insect directory of the TRANSFAC database. Boxes delimit TFBSs identified by MATCH searches as having 100% similarity at the core and at least 70% overall similarity to position weight matrices at the insect directory of the TRANSFAC database. h1, h2…: Haplotype 1, 2…. me: *D. melanogaster*, si: *D. simulans*, se: *D. sechellia*, ma: *D. mauritiana*, ya: *D. yakuba*, te: *D. teissieri*, or: *D. orena*, er: *D. erecta*. Underlined: Nucleotides shared between two different transcription factor-binding regions identified by PATCH. Double underlined: nucleotides shared between three different transcription factor-binding regions identified by PATCH. Strikethrough: Nucleotides at the core sequence of a TFBS identified by MATCH. DNase-I footprinting data are those in references [29,39,40] of the manuscript. S: Start of the DNase-I footprinted sequence. E: End of the DNase-I footprinted sequence. P: Transcription factor binding position. The arrow marks the *ftz*’s transcription start. ^1^: Here it is assumed that the ancestor was polymorphic for the nucleotides at this position some of which were subsequently fixed in some clades/branches. ^2^: It is assumed that a nucleotide that is polymorphic in *D. simulans* or in *D. yakuba* —of which more than one line were analyzed— is very likely also polymorphic in their respective geographical daughter/sister species *D. sechellia*, *D. mauritiana* or in *D. teissieri* —of which only a single line was analyzed. The aim of rules ^1^ and ^2^ is to avoid noise caused by false positives in fixed substitutions. Sequences in GenBank accession numbers HQ693575- HQ693658.

**Key to TFBSs identified by PATCH search of the insect directory of the TRANSFAC database using perfect matches and 5 bp minimum size as criteria.**

**ACTIVATOR OF ALCOHOL DEHYDROGENASE FACTOR 1**

**CAUDAL**

**GAGA FACTOR**

***fushi tarazu* Repressing Element 1**

**FUSHI TARAZU FACTOR 1**

***fushi tarazu* Dual Element 1**

***fushi tarazu* Repressing Element 2**

***fushi tarazu* Repressing Element 3**

**TRAMTRACK 69K**

***fushi tarazu* Dual Element 2**

**B FACTOR**

**BOUNDARY ELEMENT ASSOCIATED FACTOR**

**ZESTE**

**HUNCHBACK**

**FUSHI TARAZU**

**ACTIVATING PROTEIN-1**

**Possible Interferon Regulatory Factor Binding Site**

**CHORION FACTOR-2**

**FARNESOID X ACTIVATED RECEPTOR**

**PANGOLIN**

**ANTENNARCPEDIA**

**UNKNOWN (see Table S3)**

**ENGRAILED**

**ECDYSONE RECEPTOR**

**TAILLESS**

**EVEN-SKIPRCPED**

**DNA REPLICATION-RELATED ELEMENT FACTOR**

**TWIST**

**Key to TFBSs identified by MATCH search of the insect directory of the TRANSFAC database respectively using 100% and 70% similarity to the core and overall position weigh matrix.**

**ABDOMINAL-B**

**ACTIVATOR OF ALCOHOL DEHYDROGENASE FACTOR-1**

**B-Factor**

**BICOID**

**BROAD-COMPLEX**

**CROCODILE**

**DEFORMED**

**DORSAL**

**ECDYSONE-INDUCED PROTEIN 74EF**

**E74-LIKE FACTOR1**

**FUSHI TARAZU**

**GLIAL CELLS MISSING**

**HAIRY**

**HEAT SHOCK FACTOR**

**HUNCHBACK**

**KRÜPRCPEL**

**PANGOLIN**

**SNAIL**

SIGNAL-TRANSDUCER AND ACTIVATOR OF **TRANSCRIPTION PROTEIN AT 92E**

**SUPPRESSOR OF HAIRLESS**

**TRAMTRACK**

**ULTRABITHORAX**

**ULTRASPIRACLE**

**ZESTE**
