# Supplementary material for: Microevolution of cis-Regulatory Elements: An Example from the Pair-Rule Segmentation Gene fushi tarazu in the Drosophila melanogaster Subgroup
Source: PLoS One. 2011 Nov 3;6(11):e27376. doi: 10.1371/journal.pone.0027376 (PMC3207857; doi:10.1371/journal.pone.0027376)
Supplement: Table S1 — Fly species, populations and lines, their origin and the colleagues who kindly offered them. (DOC) [file pone.0027376.s002.doc]

| **Table S1.** **Fly species, populations and lines, their origin and the colleagues who kindly offered them.** | | | |
| --- | --- | --- | --- |
| Species | Population | Line | Obtained from |
| *D. melanogaster* | Netherlands | N04 | Dr. Penelope Haddrill and Dr. Peter Andolfatto, Ashworth Laboratories, University of Edinburgh, Scotland |
|  |  | N11 |  |
|  |  | N22 |  |
|  |  | N25 |  |
|  |  | N29 |  |
|  | Gabon | G121 |  |
|  |  | G130 |  |
|  |  | G139 |  |
|  |  | G141 |  |
|  |  | G185 |  |
|  | Kenya | Ky23 |  |
|  | Oregon | Oregon R | Dr. Rhonda R. Snook, University of Sheffield, UK. |
|  | Pennsylvania | P02 | Dr. Penelope Haddrill and Dr. Peter Andolfatto, Ashworth Laboratories, University of Edinburgh, Scotland |
|  | Zimbabwe | Zw104 |  |
|  |  | Zw186 |  |
| D. simulans | California | CS2A | Dr. Penelope Haddrill and Dr. Peter Andolfatto, Ashworth Laboratories, University of Edinburgh, Scotland |
|  | France | FS16 |  |
|  |  | FS19 |  |
|  |  | FS24 |  |
|  |  | FS32 |  |
|  |  | FS60 |  |
|  | Gabon | GN70 |  |
|  |  | GN154 |  |
|  |  | GN169 |  |
|  |  | GN170 |  |
|  |  | GN200 |  |
|  | Guatemala | N/A | The Tucson Species Resource Centre, stock number 14021-0251.161 |
|  | Kenya | KS01 | Dr. Penelope Haddrill and Dr. Peter Andolfatto, Ashworth Laboratories, University of Edinburgh, Scotland |
|  | Madagascar | MD06 |  |
| *D. sechellia* | Cousin Island, Seychelles | N/A | The Tucson Species Resource Centre, stock number 14021-0248.2 |
| *D. mauritiana* | Mauritius | N/A | The Tucson Species Resource Centre, stock number 14021-0241.4 |
| *D. orena* | Unknown | N/A | Dr. Harmit S. Malik, Fred Hutchinson Cancer Center, Seattle, USA. |
| *D. erecta* | Unknown | N/A | The Tucson Species Resource Centre, stock number 14021-0224.0 |
| *D. teissieri* | Unknown | N/A | The Tucson Species Resource Centre, stock number 14021-0257.0 |
| D. yakuba | Cameroon | CY03 | Dr. Penny Haddrill and Dr. Peter Andolfatto, Ashworth Laboratories, University of Edinburgh, Scotland |
|  |  | CY07 |  |
|  |  | CY19 |  |
|  |  | CY29 |  |
|  |  | CY33 |  |
|  | Gabon | GY42 |  |
|  |  | GY43 |  |
|  |  | GY45 |  |
|  |  | GY49 |  |
|  |  | GY167 |  |
|  | Ivory Coast | N/A | The Tucson Species Resource Centre, stock number 14021-0261.0 |
