# Supplementary material for: Microevolution of cis-Regulatory Elements: An Example from the Pair-Rule Segmentation Gene fushi tarazu in the Drosophila melanogaster Subgroup
Source: PLoS One. 2011 Nov 3;6(11):e27376. doi: 10.1371/journal.pone.0027376 (PMC3207857; doi:10.1371/journal.pone.0027376)
Supplement: Table S2 — Primers used for PCR amplification and cycle sequencing of the RCPE and ZE DNAs. Selection of the primers was based on conservation between the aligned sequences of D. melanogaster and D. pseudoobscura available at GenBank (accession numbers AE003673 and AY190944). *: Both D. orena and D. erecta have a 9 bp deletion within the RCPE reverse primer, underlined bases, so RCPE reverse2 was used as alternative reverse primer for amplifying the RCPE from these two species. (DOC) [file pone.0027376.s003.doc]

| **Table S2.** **Primers used for PCR amplification and cycle sequencing of the RCPE and ZE DNAs.** | | |
| --- | --- | --- |
| Primer | Sequence 5’ 3’ | Melting temperature |
| RCPE forward | GCGCGACTTCGATTCCCCGG | 62 |
| RCPE reverse^*^ | CTTTGAAGTYGCTGCTGATC | 46 |
| RCPE reverse2 (for *D. erecta* and *D. orena*)^*^ | AATATTTGAAACTTCAAAAG | 41 |
| RCPE internal forward for sequencing | AAGTGTGACAGGAGCAATTA | 41 |
| RCPE internal reverse for sequencing | CGGCACCAATCAAAGTCAAGG | 57 |
| ZE forward | GCAAATACCGGGCACAGGAGT | 54 |
| ZE reverse | GGCAGGCTGTGGGGGTGATA | 55 |
| ZE internal forward for sequencing | AATGGGCGCGATGGGTAGGT | 77 |
| ZE internal reverse for sequencing | TACCTGCTGYGCGGCTTATT | 54 |
| Selection of the primers was based on conservation between the aligned sequences of *D. melanogaster* and *D. pseudoobscura* available at GenBank (accession numbers AE003673 and AY190944). *: Both *D. orena* and *D. erecta* have a 9 bp deletion within the RCPE reverse primer, underlined bases, so RCPE reverse2 was used as alternative reverse primer for amplifying the RCPE from these two species. | | |
