# Supplementary material for: Microevolution of cis-Regulatory Elements: An Example from the Pair-Rule Segmentation Gene fushi tarazu in the Drosophila melanogaster Subgroup
Source: PLoS One. 2011 Nov 3;6(11):e27376. doi: 10.1371/journal.pone.0027376 (PMC3207857; doi:10.1371/journal.pone.0027376)
Supplement: Table S3 — Transcription factor binding sites identified in haplotypes of the Drosophila region containing ftz proximal enhancer (RCPE) and zebra element (ZE) and estimation of the likelihood of ftz regulation by their binding proteins based on our knowledge on gene expression and function. The search was performed using the insect directory of the TRANSFAC® 6.0 database [99,100,101,102,103] with the help of the programs PATCH™ public 1.0 (limited to perfect matches of at least 5 bases) and MATCH™ public 1.0 [104] (using 70% as a minimum overall similarity and 100% as core similarity cut-offs). ftz is a segmentation gene with neurogenic involvement it is known to be active late in development [105] but not studied during metamorphosis; although it wouldńt be unwise to expect it to be expressed throughout that period, especially since it is activated by the nuclear hormone receptor FTZ-F1 (see above). It has multiple positive and negative regulators that shape the variation of its spacio-temporal expression. When a gene is not known to regulate ftz it was assessed based on its spacio-temporal expression, its position downstream of ftz and the possibility of a feedback, its involvement in segmentation, neurogenesis or molting/metamorphosis. It is also worth mentioning that, while the zebra element and the proximal enhancer-containing sequences analyzed in this work are of ∼800 bp and 1500 bp respectively, the experimental testing of transcription factor binding to these elements has been performed only for parts of these sequences (about 400 bp (see [22,98,106])). (DOC) [file pone.0027376.s004.doc]

| **Table S3. Transcription factor binding sites identified in haplotypes of the *Drosophila* region containing *ftz* proximal enhancer (RCPE) and zebra element (ZE) and estimation of the likelihood of *ftz* regulation by their binding proteins based on our knowledge on gene expression and function.** | | | |
| --- | --- | --- | --- |
| Element |  | Transcription-factor–binding site | |
|  | Likelihood of the interaction with *ftz* | Name | Function of the factor bound or its gene and assessment of its potential involvement in *ftz* regulation |
| RCPE | Known to regulate *ftz* activity and the binding of the transcription factor to sequences in the element has been experimentally proven | *fushi tarazu* (*ftz*) | FTZ is a member of the Q50 homeodomain proteins. It has a positive feed-back on its gene through the proximal and the distal enhancer [1,2,3]. |
|  |  | GAGA, or Trithorax-Like, Factor | Functions in counteracting chromatin repression at all levels by influencing chromatin structure and, by so doing, triggers the active transcription of genes subject to repression [4]. It is a suggested *ftz* activator [5]. |
|  |  | *snail* (*sn*) | Gap gene required for proper mesoderm formation. It represses the ventro-lateral genes in the mesoderm, restricts neuroectoderm and neural fate in the invaginating mesoderm, and is a *ftz* regulator [6]. |
|  |  | *tramtrack* (*Ttk*) | TTK 69k protein, also called FTZ-F2, was isolated on the basis of its binding to *ftz*’s zebra element, where it has a repressive action. It is also expressed in the peripheral nervous system, and has a dual function by serving negative and positive regulatory roles at different stages of photoreceptor development [7]. |
|  | Known to regulate *ftz* activity but the binding of the transcription factor to sequences in the element has not been experimentally tested | Activator of Alcohol Dehydrogenase Factor 1 (ADF-1) | May play an essential role in terminal stages of neuronal differentiation and function [8] and it is suspected to interact with *ftz* [9]. |
|  |  | B, or TATA, Factor | This factor directs transcription by cellular RNA polymerases and mediates activation of *ftz* [10]. |
|  |  | *caudal* (*cad*) | Maternally and zygotically expressed, caudal protein forms a posterior-to-anterior concentration gradient [11], and activates *ftz*’s transcription in the embryo's posterior [12]. |
|  |  | *engrailed* (*en*) | Like FTZ, EN is a member of the Q50 homeodomain proteins. e*n* is a segment polarity gene. It expression starts at the end of cellularization and is localized in 14 even-numbered stripes (*i.e.*, overlapping with *ftz*). *en* is activated by *ftz* [13,14,15] and seems to have a negative feedback on it [16]. |
|  |  | *even-skipped* (*eve*) | Pair rule segmentation gene [17]. Complementary to *ftz* , it confers identity to the odd-numbered parasegments where it is expressed [18]. EVE is a repressor of a number of genes including *ftz* [19]. |
|  |  | *hairy* (*h*) | Pair-rule gene whose product interacts with groucho protein; a mediator of maternal influences on neurogenesis, segmentation and sex determination [20]. It is essential for peripheral nervous system development [21] and is a known activator of *ftz* [22]. |
|  |  | *hunchback* (*hb*) | Gap segmentation gene [23] involved in the zygotic determination of anterior-posterior axis as well as in the torso signalling pathway, ventral cord development, ganglion mother cell fate determination, neuroblast cell fate determination, tracheal system development and salivary gland development (see [24]). It can function synergistically together with *ftz* [25] and is known to affect its expression [26]. |
|  |  | *Krüppel* (*Kr*) | Gap gene necessary for segmentation [27]. It represses *engrailed*, *hairy* in stripe 6 and *even-skipped* stripe 2 element, but activates *hairy* in stripes 3 to 5. It is a segmentation regulator of *ftz* as well [28,29]. |
|  |  | Signal-transducer and activator of transcription protein at 92E (*STAT*) | Also known as *marelle* (*mrl*). It is present in the egg at the time of fertilization. Early in development it is expressed in a pair-rule distribution, but later in a 14 striped segment polarity pattern. It regulates even-skipped stripe 3 promoter and the pair rule gene runt *—*both *ftz* repressors [22]— [30,31,32]. It is also a *ftz* regulator [33]. |
|  |  | *tailless* (*tll*) | Required for the establishment of the posterior and anterior domains of the embryo [34,35]. Its products define the positions of the germ layers *primordia* and thereby the invagination regions of the blastodermal epithelium [36]. *tll* functions to drive cells to the optic lobe in the developing embryonic visual system [37] and is active in the early embryonic head and the protocerebral brain anlage [38]. It represses *ftz* in the anterior domains [39]. |
|  | Potential *ftz* regulators but there is so far no supporting empirical evidence | *Abdominal-B* (*Adb-B*) | Segment-polarity gene also expressed in the central nervous system (ventral cord) at stage 12 of drosophila development [40,41]. It is therefore a possible segmentation and/or neurogenic regulator (feedback) of *ftz* —a gene known to regulate Abdominal A [42]. |
|  |  | Activating Protein-1 (AP-1) | Is a heterodimer formed by C-JUN and C-FOS. This association is required for binding to the promoter region of many genes. Its involvement in *ftz* regulation during neurogenesis is possible as it is tightly linked to some neural functions [43,44] as well as eye development [45,46,47]. |
|  |  | *Antennapedia* (*Antp*) | *hox* gene regulated by *ftz* [48], to which it is distal [49,50,51], so I cannot rule out a possible feedback of this gene on *ftz*. |
|  |  | *bicoid* (*bcd*) | Maternal effect gene [52] and concentration-dependent activator of eve stripe 2 element and hairy in stripe 1. The product of this gene, a broadly bound transcription factor, is a major determinant of antero-posterior axis of the embryo [52,53,54,55]. It is therefore a potential segmentation regulator (suppressor) of *ftz*. |
|  |  | Boundary Element Associated Factor (BEAF) | This is a protein that binds to boundary elements to interfere with interactions between different enhancers and promoters [56]. Its function in the *cis*-regulatory elements studied in this work cannot be ruled out —especially since it is located in potential inter-element locations of the sequences (*i.e.* between *ftz* zebra element and coding sequence and by the last 200 bp of the ~1.5 kb sequence containing *ftz* proximal enhancer analyzed in this work). |
|  |  | *Broad-Complex* (*Br-C*) | Codes for multiple protein isoforms that are widely distributed among all tissues examined in late larval to pre-pupal stages of development, and have different functions in regulation of genes activated late in the molting hierarchy [57,58]. Thus, its possible involvement in a potential *ftz* regulation during metamorphosis cannot be ruled out. |
|  |  | *crocodile* (*croc*) | Seems to play a role in the development of the brain and central nervous system, where it may be regulating *ftz*, as it is expressed in precursors of the ventral cord in a segmentaly repeated fashion [59]. It is also a regulator of *engrailed* and *wingless*, two FTZ targets [60]. |
|  |  | *Deformed* (*Dfd*) | Homeotic selector responsible for the normal development of the maxillary segment. It is also needed for the normal separation of the subesophageal ganglion from the thoracic ganglion during metamorphosis [61]. It is suppressed by *ftz* [62] so potential ‘negative feedback’ of *Dfd* on *ftz* cannot be ruled out. |
|  |  | *dorsal* (*dl*) | A morphogen that establishes dorsal-ventral polarity during embryogenesis [63]. I can therefore not rule out its involvement in the regulation of *ftz*. |
|  |  | DNA Replication-Related Element Factor (DREF) | Required for normal DNA replication in both the mitotic cell cycle and the endo cycle [64]. There are no data on its possible interaction with *ftz*, but it is an activator of *caudal* [65]; which activates *ftz* (see above and text of the manuscript). I therefore chose to include it in the analyses as it may be a *ftz* activator. |
|  |  | E74-Like Factor-1 (ELF-1) | Also known as Neural Transcription Factor-1 (NTF-1). It has some similarity with E74 and is known to interact with *ftz* neurogenic element [66]. Therefore, I cannot rule out its interaction with other *ftz* elements as well. |
|  |  | Ecdysone-Induced Protein 74EF (E74A) | Among other involvements, E74A is required for metamorphosis [67,68,69] where it may be regulating any potential *ftz* activity. |
|  |  | *Ecdysone receptor* (*Ecr*) | Among its functions, ecdysone is required for the timing of metamorphosis and the induction and repression of genes required for the differentiation process [70]. It negatively regulates beta-FTZ-F1 (a transcription factor that activates *ftz* through its zebra element) providing a molecular mechanism for stage-specific responses to steroid hormones [71]. It is therefore a potential regulator of *ftz* during metamorphosis. |
|  |  | *glial cells missing* (*gcm*) | Expressed in almost all glia as soon as they are born. If GCM is lacking, presumptive glial cells are transformed into neurons. If *gcm* is expressed in presumptive neurons, these cells transform into glia [72]. Since *ftz* expression in the developing CNS includes glia [73], *gcm* could therefore be a neurogenic regulator of *ftz*. |
|  |  | Heat Shock Factor (HSF) | Affects gene transcription in response to heat stress [74]. Its involvement in *ftz* regulation cannot be ruled out. |
|  |  | *pangolin* (*pan*) | PAN is product of a segment polarity gene (*pan*) that interacts with the product of the gene *armadillo* and is an essential component of the multifunction Wnt/Wingless (Wg) transduction pathway acting directly to regulate gene transcription in response to members of the so important Wg family of signaling proteins [75]. This gene, also referred to as dTCF (Drosophila’s homolog of mammalian T Cell Factor), can therefore not be ruled out as possible regulator of *ftz*. |
|  |  | Similar to a mammalian Interferon Regulatory Factor Binding Site | This sequence, AACTGA, seems to be a 3´ binding site for a mammalian interferon regulatory factor [76]. It seems significant that our *ftz* *cis*-regulatory sequences contain multiple AACTGA copies (the likelihood of a six base pairs sequence appearing more than once in a 1.5 kb sequence is negligible). More important is the fact that this sequence tends to appear in the close vicinity of *ftz* binding sites, but only in the *ftz* RCPE element. I therefore cannot rule out the potential authenticity of this hexamer and I prefer to consider it as potential regulator of *ftz* activity through its proximal enhancer. |
|  |  | *Suppressor of Hairless* (*Su(H)*) | Integral part of the all important Notch signalling pathway and is neurogenic. SU(H) protein is both cytoplasmic, where it interacts with the trans-membrane Notch receptor that receives neurogenic signals from outside the cell, and nuclear, where it carries the Notch signal and acts as a transcription factor regulating neurogenesis [77,78,79]. Its involvement in *ftz* regulation during neurogenesis is therefore possible. |
|  |  | *twist* (*Twi*) | *twist* has no effect on the striped expression of *ftz* in *D. melangaster* [80]. Accordingly it wasn´t identified in any haplotype of this species. However one conserved site was identified in *D. orena* and *D. erecta*. I can’t rule out the authenticity of these potentially ‘*de novo*’ sites as *twist* is an activator of downstream mesoderm genes [81] and *twist*-expressing cells associate with the segmental nerves in the thoracic as well as the abdominal segments of the third larval instar [82]. Its involvement in *ftz* regulation in the mesoderm and the developing neural system of some Drosophila species may thus be possible. |
|  |  | *Ultrabithorax* (*Ubx*) | A homeotic selector required for the proper development of thoracic structures [83]. It is regulated by *ftz* [84,85] on which it may have some sort of feedback. |
|  |  | *ultraspiracle*  (*usp*) | Maternally and zygotically expressed and, among other functions, an activator of genes involved in molting. Among its multiple functions in development, it is required in the eye-antennal imaginal disc and for normal eye morphogenesis [86]. Its neurogenic and/or metamorphosis involvement in *ftz* regulation can therefore not be ruled out. |
|  |  | Unknown | This sequence, TACTAA, is involved in activating the *Adh* gene [87] and seems to be closely linked with promoters, so I cannot rule out its involvement in *ftz* regulation. |
|  |  | *zeste* (*z*) | Z is a gene-activating protein, including in *trans*, that also reduces position effect variegation [88]. It is a redundant activator of *Ubx* [89] and I cannot rule out its involvement in *ftz* regulation. |
|  | Not likely to regulate *ftz* activity (false positive) | *Chorion Factor-2* (*CF-2*) | Myogenic and late activator in follicle cells during chorion formation [90,91]. With no clear involvement in segmentation nor in neurogenesis, I prefer to consider this binding site as potentially false positive and exclude it from the analyses as not likely to regulate *ftz*. |
|  |  | Similar to Farnesoid X Activated Receptor (FXR) | This sequence is similar to the binding site of the mammalian ligand-modulated transcription factor FXR which forms a heterodimer with retinoid X receptor alpha (RXR-Alpha). FXR is expressed in the liver, kidney and gut in vertebrate embryos and adults [92]. I therefore prefer to consider this sequence as false positive and exclude it from the analyses as its Drosophila binding protein is not known and, if any, it would be unlikely to regulate *ftz*. |
| ZE | Known to regulate *ftz* activity and the binding of the transcription factor to sequences in the element has been experimentally proven | ADF-1 | See above. |
|  |  | B Factor | See above. |
|  |  | *Cad* | See above. |
|  |  | *Ftz* | See above. |
|  |  | *ftz* Dual Element 1 (fDE1) | This is a 32-bp site through which both *runt* and *hairy* gene products respectively activate and repress *ftz*. It is also a binding site for FTZ-F1 [22]. |
|  |  | fDE2 | This is a site considered to be functionally redundant to fDE1 —for a purpose of fine tuning of *ftz* expression— as it is another site with a dual function in *ftz* activation and repression, depending on the transcription factor(s) bound to it. It also contains a second low affinity FTZ-F1 binding site [22]. |
|  |  | *ftz* Factor 1 (FTZ-F1) | With two isoforms, *Alpha and Beta* [93], FTZ-F1 is a nuclear hormone receptor and activator of *ftz* through its zebra element [94]. Besides binding to several sites in the zebra element, FTZ-F1 binds to additional sites within the *ftz* gene [95]. Both this and the FTZ protein are mutually dependent cofactors [96]. Among their functions, FTZ-F1 *Alpha* is the isoform involved in the segmentation expression of *ftz* [97]. Whereas, FTZ-F1 *Beta* contributes to the regulation of a significant fraction of the genes in the late prepupal phase of the molting regulatory hierarchy, and its action may serve as a bridge between early and late gene expression during the process of metamorphosis (early genes include *Ecdysone receptor*) [70,71]. |
|  |  | *ftz* Repressing Element 1 (fRE1) | *ftz* repressing element 1 [98]. |
|  |  | fRE2 | *ftz* repressing element 2 [98]. |
|  |  | fRE3 | *ftz* repressing element 3 [98]. |
|  |  | GAGA Factor | See above. |
|  |  | *Ttk* | See above. |
|  | Known to regulate *ftz* activity but the binding of the transcription factor to sequences in the element has not been experimentally tested | *H* | See above. |
|  |  | *Hb* | See above. |
|  |  | *Kr* | See above. |
|  |  | *STAT* | See above. |
|  |  | *Sn* | See above. |
|  | Potential *ftz* regulators but there is so far no supporting empirical evidence | *Abd-B* | See above. |
|  |  | *Bcd* | See above. |
|  |  | BEAF | See above. |
|  |  | *Br-C* | See above. |
|  |  | *Croc* | See above. |
|  |  | *Dfd* | See above. |
|  |  | *Dl* | See above. |
|  |  | E74A | See above. |
|  |  | ELF-1 | See above. |
|  |  | *Gcm* | See above. |
|  |  | HSF | See above. |
|  |  | *Ubx* | See above. |
|  |  | *Usp* | See above. |
|  |  | *Z* | See above. |
|  | Not likely to regulate *ftz* activity (false positive) | CF2 | See above. |
| The search was performed using the insect directory of the TRANSFAC® 6.0 database [99,100,101,102,103] with the help of the programs PATCH^TM^ public 1.0 (limited to perfect matches of at least 5 bases) and MATCH^TM^ public 1.0 [104] (using 70% as a minimum overall similarity and 100% as core similarity cut-offs). *ftz* is a segmentation gene with neurogenic involvement it is known to be active late in development [105] but not studied during metamorphosis; although it wouldn´t be unwise to expect it to be expressed throughout that period, especially since it is activated by the nuclear hormone receptor FTZ-F1 (see above). It has multiple positive and negative regulators that shape the variation of its spacio-temporal expression. When a gene is not known to regulate *ftz* it was assessed based on its spacio-temporal expression, its position downstream of *ftz* and the possibility of a feedback, its involvement in segmentation, neurogenesis or molting/metamorphosis. It is also worth mentioning that, while the zebra element and the proximal enhancer-containing sequences analyzed in this work are of ~800 bp and 1500 bp respectively, the experimental testing of transcription factor binding to these elements has been performed only for parts of these sequences (about 400 bp (see [22,98,106])).  **References**  1. Ingham P, Gergen P (1988) Interactions between the pair-rule genes *runt*, *hairy*, *even-skipped* and *fushi tarazu* and establishment of periodic pattern in the *Drosophila* embryo. Development 104 supplement: 51-60.  2. Dearolf CR, Topol J, Parker CS (1989) Transcriptional control of *Drosophila fushi tarazu* zebra stripe expression. Genes Dev 3: 384-398.  3. Dearolf CR, Topol J, Parker CS (1990) Transcriptional regulation of the *Drosophila* segmentation gene *fushi tarazu* (*ftz*). Bioessays 12: 109-113.  4. Granok H, Leibovitch BA, Shaffer CD, Elgin SC (1995) Chromatin. Ga-ga over GAGA factor. Curr Biol 5: 238-241.  5. Okada M, Hirose S (1998) Chromatin remodeling mediated by Drosophila GAGA factor and ISWI activates *fushi tarazu* gene transcription *in vitro*. Mol Cell Biol 18: 2455-2461.  6. Ashraf SI, Hu X, Roote J, Ip YT (1999) The mesoderm determinant snail collaborates with related zinc-finger proteins to control *Drosophila* neurogenesis. Embo J 18: 6426-6438.  7. Lai ZC, Li Y (1999) *Tramtrack69* is positively and autonomously required for *Drosophila* photoreceptor development. Genetics 152: 299-305.  8. DeZazzo J, Sandstrom D, de Belle S, Velinzon K, Smith P, et al. (2000) nalyot, a mutation of the *Drosophila* *myb*-related *Adf1* transcription factor, disrupts synapse formation and olfactory memory. Neuron 27: 145-158.  9. Han W, Yu Y, Su K, Kohanski RA, Pick L (1998) A binding site for multiple transcriptional activators in the *fushi tarazu* proximal enhancer is essential for gene expression *in vivo*. Mol Cell Biol 18: 3384-3394.  10. Colgan J, Wampler S, Manley JL (1993) Interaction between a transcriptional activator and transcription factor IIB in vivo. Nature 362: 549-553.  11. Schulz C, Tautz D (1995) Zygotic *caudal* regulation by *hunchback* and its role in abdominal segment formation of the *Drosophila* embryo. Development 121: 1023-1028.  12. Dearolf CR, Topol J, Parker CS (1989) The caudal gene product is a direct activator of fushi tarazu transcription during *Drosophila* embryogenesis. Nature 341: 340-343.  13. Saenz-Robles MT, Maschat F, Tabata T, Scott MP, Kornberg TB (1995) Selection and characterization of sequences with high affinity for the *engrailed* protein of *Drosophila*. Mech Dev 53: 185-195.  14. Nasiadka A, Grill A, Krause HM (2000) Mechanisms regulating target gene selection by the homeodomain-containing protein Fushi tarazu. Development 127: 2965-2976.  15. Nasiadka A, Krause HM (1999) Kinetic analysis of segmentation gene interactions in *Drosophila* embryos. Development 126: 1515-1526.  16. Smith ST, Jaynes JB (1996) A conserved region of *engrailed*, shared among all *en-*, *gsc-*, *Nk1-*, *Nk2-* and *msh-*class homeoproteins, mediates active transcriptional repression *in vivo*. Development 122: 3141-3150.  17. Frasch M, Levine M (1987) Complementary patterns of *even-skipped* and *fushi tarazu* expression involve their differential regulation by a common set of segmentation genes in *Drosophila*. Genes Dev 1: 981-995.  18. Frasch M, Hoey T, Rushlow C, Doyle H, Levine M (1987) Characterization and localization of the *even-skipped* protein of *Drosophila*. Embo J 6: 749-759.  19. Fujioka M, Jaynes JB, Goto T (1995) Early *even-skipped* stripes act as morphogenetic gradients at the single cell level to establish *engrailed* expression. Development 121: 4371-4382.  20. Paroush Z, Finley RL, Jr., Kidd T, Wainwright SM, Ingham PW, et al. (1994) *Groucho* is required for *Drosophila* neurogenesis, segmentation, and sex determination and interacts directly with *hairy*-related bHLH proteins. Cell 79: 805-815.  21. Van Doren M, Bailey AM, Esnayra J, Ede K, Posakony JW (1994) Negative regulation of proneural gene activity: *hairy* is a direct transcriptional repressor of *achaete*. Genes Dev 8: 2729-2742.  22. Tsai C, Gergen P (1995) Pair-rule expression of the *Drosophila fushi tarazu* gene: a nuclear receptor response element mediates the opposing regulatory effects of *runt* and *hairy*. Development 121: 453-462.  23. Tautz D (1988) Regulation of the *Drosophila* segmentation gene *hunchback* by two maternal morphogenetic centres. Nature 332: 281-284.  24. Wu X, Vasisht V, Kosman D, Reinitz J, Small S (2001) Thoracic patterning by the *Drosophila* gap gene *hunchback*. Dev Biol 237: 79-92.  25. Zuo P, Stanojevic D, Colgan J, Han K, Levine M, et al. (1991) Activation and repression of transcription by the gap proteins *hunchback* and *Kruppel* in cultured *Drosophila* cells. Genes Dev 5: 254-264.  26. Carroll SB, Scott MP (1986) Zygotically active genes that affect the spatial expression of the *fushi tarazu* segmentation gene during early *Drosophila* embryogenesis. Cell 45: 113-126.  27. Preiss A, Rosenberg UB, Kienlin A, Seifert E, Jackle H (1985) Molecular genetics of *Kruppel*, a gene required for segmentation of the *Drosophila* embryo. Nature 313: 27-32.  28. Harrison SD, Travers AA (1988) Identification of the binding sites for potential regulatory proteins in the upstream enhancer element of the *Drosophila fushi tarazu* gene. Nucleic Acids Res 16: 11403-11416.  29. Ingham PW, Ish-Horowicz D, Howard KR (1986) Correlative changes in homoeotic and segmentation gene expression in *Kruppel* mutant embryos of *Drosophila*. Embo J 5: 1659-1665.  30. Hou XS, Melnick MB, Perrimon N (1996) *Marelle* acts downstream of the *Drosophila HOP/JAK* kinase and encodes a protein similar to the mammalian STATs. Cell 84: 411-419.  31. Yan R, Luo H, Darnell JE Jr., Dearolf CR (1996) A *JAK-STAT* pathway regulates wing vein formation in *Drosophila*. Proc Natl Acad Sci U S A 93: 5842-5847.  32. Yan R, Small S, Desplan C, Dearolf CR, Darnell JE Jr. (1996) Identification of a *Stat* gene that functions in *Drosophila* development. Cell 84: 421-430.  33. Harrison DA, McCoon PE, Binari R, Gilman M, Perrimon N (1998) *Drosophila* unpaired encodes a secreted protein that activates the *JAK* signaling pathway. Genes Dev 12: 3252-3263.  34. Strecker TR, Kongsuwan K, Lengyel JA, Merriam JR (1986) The zygotic mutant *tailless* affects the anterior and posterior ectodermal regions of the *Drosophila* embryo. Dev Biol 113: 64-76.  35. Strecker TR, Merriam JR, Lengyel JA (1988) Graded requirement for the zygotic terminal gene, *tailless*, in the brain and tail region of the *Drosophila* embryo. Development 102: 721-734.  36. Leptin M (1994) Morphogenesis. Control of epithelial cell shape changes. Curr Biol 4: 709-712.  37. Daniel A, Dumstrei K, Lengyel JA, Hartenstein V (1999) The control of cell fate in the embryonic visual system by *atonal*, *tailless* and *EGFR* signaling. Development 126: 2945-2954.  38. Hartmann B, Reichert H, Walldorf U (2001) Interaction of gap genes in the *Drosophila* head: *tailless* regulates expression of *empty spiracles* in early embryonic patterning and brain development. Mech Dev 109: 161-172.  39. Reinitz J, Levine M (1990) Control of the initiation of homeotic gene expression by the gap genes *giant* and *tailless* in *Drosophila*. Dev Biol 140: 57-72.  40. DeLorenzi M, Ali N, Saari G, Henry C, Wilcox M, et al. (1988) Evidence that the *Abdominal-B* r element function is conferred by a trans-regulatory homeoprotein. EMBO J 7: 3223-3231.  41. Delorenzi M, Bienz M (1990) Expression of *Abdominal-B* homeoproteins in *Drosophila* embryos. Development 108: 323-329.  42. Macias A, Pelaz S, Morata G (1994) Genetic factors controlling the expression of the *abdominal-A* gene of *Drosophila* within its domain. Mech Dev 46: 15-25.  43. Karin M, Liu Z, Zandi E (1997) AP-1 function and regulation. Curr Opin Cell Biol 9: 240-246.  44. Johnson RS, Spiegelman BM, Papaioannou V (1992) Pleiotropic effects of a null mutation in the *c-fos* proto-oncogene. Cell 71: 577-586.  45. Kockel L, Zeitlinger J, Staszewski LM, Mlodzik M, Bohmann D (1997) *Jun* in *Drosophila* development: redundant and nonredundant functions and regulation by two MAPK signal transduction pathways. Genes Dev 11: 1748-1758.  46. Glise B, Noselli S (1997) Coupling of *Jun* amino-terminal kinase and *Decapentaplegic* signaling pathways in *Drosophila* morphogenesis. Genes Dev 11: 1738-1747.  47. Hou YN, Cebers G, Terenius L, Liljequist S (1997) Characterization of NMDA- and AMPA-induced enhancement of AP-1 DNA binding activity in rat cerebellar granule cells. Brain Res 754: 79-87.  48. Riley GR, Jorgensen EM, Baker RK, Garber RL (1991) Positive and negative control of the *Antennapedia* promoter P2. Dev Suppl 1: 177-185.  49. Abbott MK, Kaufman TC (1986) The relationship between the functional complexity and the molecular organization of the *Antennapedia* locus of *Drosophila melanogaster*. Genetics 114: 919-942.  50. Stroeher VL, Jorgensen EM, Garber RL (1986) Multiple transcripts from the *Antennapedia* gene of *Drosophila melanogaster*. Mol Cell Biol 6: 4667-4675.  51. Reuter R, Scott MP (1990) Expression and function of the homoeotic genes *Antennapedia* and *Sex combs reduced* in the embryonic midgut of *Drosophila*. Development 109: 289-303.  52. Struhl G, Struhl K, Macdonald PM (1989) The gradient morphogen *bicoid* is a concentration-dependent transcriptional activator. Cell 57: 1259-1273.  53. Hanes SD, Brent R (1989) DNA specificity of the *bicoid* activator protein is determined by homeodomain recognition helix residue 9. Cell 57: 1275-1283.  54. Hoch M, Seifert E, Jackle H (1991) Gene expression mediated by *cis*-acting sequences of the *Kruppel* gene in response to the *Drosophila* morphogens *bicoid* and *hunchback*. EMBO J 10: 2267-2278.  55. Frohnhofer HG, Nusslein-Volhard C (1986) Organization of anterior pattern in the *Drosophila* embryo by the maternal gene *bicoid*. Nature 324: 120-125.  56. Hart CM, Zhao K, Laemmli UK (1997) The scs' boundary element: characterization of boundary element-associated factors. Mol Cell Biol 17: 999-1009.  57. Emery IF, Bedian V, Guild GM (1994) Differential expression of *Broad-Complex* transcription factors may forecast tissue-specific developmental fates during *Drosophila* metamorphosis. Development 120: 3275-3287.  58. Huet F, Ruiz C, Richards G (1993) Puffs and PCR: the *in vivo* dynamics of early gene expression during ecdysone responses in *Drosophila*. Development 118: 613-627.  59. Hacker U, Grossniklaus U, Gehring WJ, Jackle H (1992) Developmentally regulated *Drosophila* gene family encoding the fork head domain. Proc Natl Acad Sci U S A 89: 8754-8758.  60. Hacker U, Kaufmann E, Hartmann C, Jurgens G, Knochel W, et al. (1995) The *Drosophila fork head* domain protein crocodile is required for the establishment of head structures. Embo J 14: 5306-5317.  61. Restifo LL, Merrill VK (1994) Two *Drosophila* regulatory genes, *deformed* and the *Broad-Complex*, share common functions in development of adult CNS, head, and salivary glands. Dev Biol 162: 465-485.  62. Jack T, McGinnis W (1990) Establishment of the *Deformed* expression stripe requires the combinatorial action of coordinate, gap and pair-rule proteins. Embo J 9: 1187-1198.  63. Belvin MP, Jin Y, Anderson KV (1995) Cactus protein degradation mediates *Drosophila* dorsal-ventral signaling. Genes Dev 9: 783-793.  64. Hirose F, Yamaguchi M, Matsukage A (1999) Targeted expression of the DNA binding domain of DRE-binding factor, a *Drosophila* transcription factor, attenuates DNA replication of the salivary gland and eye imaginal disc. Mol Cell Biol 19: 6020-6028.  65. Choi YJ, Choi TY, Yamaguchi M, Matsukage A, Kim YS, et al. (2004) Transcriptional regulation of the *Drosophila caudal* homeobox gene by DRE/DREF. Nucleic Acids Res 32: 3734-3742.  66. Dynlacht BD, Attardi LD, Admon A, Freeman M, Tjian R (1989) Functional analysis of NTF-1, a developmentally regulated *Drosophila* transcription factor that binds neuronal *cis*-elements. Genes Dev 3: 1677-1688.  67. Fletcher JC, Burtis KC, Hogness DS, Thummel CS (1995) The *Drosophila E74* gene is required for metamorphosis and plays a role in the polytene chromosome puffing response to ecdysone. Development 121: 1455-1465.  68. Fletcher JC, Thummel CS (1995) The ecdysone-inducible *Broad-complex* and *E74* early genes interact to regulate target gene transcription and *Drosophila* metamorphosis. Genetics 141: 1025-1035.  69. Fletcher JC, Thummel CS (1995) The *Drosophila E74* gene is required for the proper stage- and tissue-specific transcription of ecdysone-regulated genes at the onset of metamorphosis. Development 121: 1411-1421.  70. Thummel CS (1995) From embryogenesis to metamorphosis: the regulation and function of *Drosophila* nuclear receptor superfamily members. Cell 83: 871-877.  71. Woodard CT, Baehrecke EH, Thummel CS (1994) A molecular mechanism for the stage specificity of the *Drosophila* prepupal genetic response to ecdysone. Cell 79: 607-615.  72. Akiyama-Oda Y, Hosoya T, Hotta Y (1998) Alteration of cell fate by ectopic expression of *Drosophila* *glial cells missing* in non-neural cells. Dev Genes Evol 208: 578-585.  73. Doe CQ, Hiromi Y, Gehring WJ, Goodman CS (1988) Expression and function of the segmentation gene *fushi tarazu* during *Drosophila* neurogenesis. Science 239: 170-175.  74. O'Brien T, Lis JT (1993) Rapid changes in *Drosophila* transcription after an instantaneous heat shock. Mol Cell Biol 13: 3456-3463.  75. Brunner E, Peter O, Schweizer L, Basler K (1997) *pangolin* encodes a Lef-1 homologue that acts downstream of *Armadillo* to transduce the *Wingless* signal in *Drosophila*. Nature 385: 829-833.  76. Meraro D, Gleit-Kielmanowicz M, Hauser H, Levi BZ (2002) IFN-stimulated gene *15* is synergistically activated through interactions between the myelocyte/lymphocyte-specific transcription factors, PU.1, IFN regulatory factor-8/IFN consensus sequence binding protein, and IFN regulatory factor-4: characterization of a new subtype of IFN-stimulated response element. J Immunol 168: 6224-6231.  77. Fortini ME, Artavanis-Tsakonas S (1994) The suppressor of hairless protein participates in notch receptor signaling. Cell 79: 273-282.  78. Furriols M, Bray S (2000) Dissecting the mechanisms of *suppressor of hairless* function. Dev Biol 227: 520-532.  79. Wettstein DA, Turner DL, Kintner C (1997) The *Xenopus* homolog of *Drosophila* *Suppressor of Hairless* mediates Notch signaling during primary neurogenesis. Development 124: 693-702.  80. Carroll SB, Winslow GM, Twombly VJ, Scott MP (1987) Genes that control dorsoventral polarity affect gene expression along the anteroposterior axis of the *Drosophila* embryo. Development 99: 327-332.  81. Leptin M (1991) *twist* and *snail* as positive and negative regulators during *Drosophila* mesoderm development. Genes Dev 5: 1568-1576.  82. Fernandes J, Vijay-Raghavan K (1993) The development of indirect flight muscle innervation in *Drosophila melanogaster*. Development 118: 215-227.  83. Struhl G (1982) Genes controlling segmental specification in the *Drosophila* thorax. Proc Natl Acad Sci U S A 79: 7380-7384.  84. Qian S, Capovilla M, Pirrotta V (1991) The *bx* region enhancer, a distant *cis*-control element of the *Drosophila Ubx* gene and its regulation by *hunchback* and other segmentation genes. Embo J 10: 1415-1425.  85. Muller J, Bienz M (1992) Sharp anterior boundary of homeotic gene expression conferred by the *fushi tarazu* protein. Embo J 11: 3653-3661.  86. Oro AE, McKeown M, Evans RM (1992) The *Drosophila* retinoid X receptor homolog *ultraspiracle* functions in both female reproduction and eye morphogenesis. Development 115: 449-462.  87. Heberlein U, England B, Tjian R (1985) Characterization of *Drosophila* transcription factors that activate the tandem promoters of the *alcohol dehydrogenase* gene. Cell 41: 965-977.  88. Judd BH (1995) Mutations of *zeste* that mediate transvection are recessive enhancers of position-effect variegation in *Drosophila melanogaster*. Genetics 141: 245-253.  89. Biggin MD, Bickel S, Benson M, Pirrotta V, Tjian R (1988) *Zeste* encodes a sequence-specific transcription factor that activates the *Ultrabithorax* promoter *in vitro*. Cell 53: 713-722.  90. Hsu T, Bagni C, Sutherland JD, Kafatos FC (1996) The transcriptional factor CF2 is a mediator of EGF-R-activated dorsoventral patterning in *Drosophila* oogenesis. Genes Dev 10: 1411-1421.  91. Hsu T, Gogos JA, Kirsh SA, Kafatos FC (1992) Multiple zinc finger forms resulting from developmentally regulated alternative splicing of a transcription factor gene. Science 257: 1946-1950.  92. Forman BM, Goode E, Chen J, Oro AE, Bradley DJ, et al. (1995) Identification of a nuclear receptor that is activated by farnesol metabolites. Cell 81: 687-693.  93. Ohno CK, Ueda H, Petkovich M (1994) The *Drosophila* nuclear receptors FTZ-F1 alpha and FTZ-F1 beta compete as monomers for binding to a site in the *fushi tarazu* gene. Mol Cell Biol 14: 3166-3175.  94. Lavorgna G, Ueda H, Clos J, Wu C (1991) FTZ-F1, a steroid hormone receptor-like protein implicated in the activation of *fushi tarazu*. Science 252: 848-851.  95. Ueda H, Sonoda S, Brown JL, Scott MP, Wu C (1990) A sequence-specific DNA-binding protein that activates *fushi tarazu* segmentation gene expression. Genes Dev 4: 624-635.  96. Guichet A, Copeland JW, Erdelyi M, Hlousek D, Zavorszky P, et al. (1997) The nuclear receptor homologue Ftz-F1 and the homeodomain protein Ftz are mutually dependent cofactors. Nature 385: 548-552.  97. Schwartz CJ, Sampson HM, Hlousek D, Percival-Smith A, Copeland JW, et al. (2001) FTZ-Factor1 and Fushi tarazu interact via conserved nuclear receptor and coactivator motifs. Embo J 20: 510-519.  98. Topol J, Dearolf CR, Prakash K, Parker CS (1991) Synthetic oligonucleotides recreate *Drosophila fushi tarazu* zebra-stripe expression. Genes Dev 5: 855-867.  99. Wingender E (2008) The TRANSFAC project as an example of framework technology that supports the analysis of genomic regulation. Brief Bioinform 9: 326-332.  100. Wingender E, Chen X, Fricke E, Geffers R, Hehl R, et al. (2001) The TRANSFAC system on gene expression regulation. Nucleic Acids Res 29: 281-283.  101. Wingender E, Chen X, Hehl R, Karas H, Liebich I, et al. (2000) TRANSFAC: an integrated system for gene expression regulation. Nucleic Acids Res 28: 316-319.  102. Wingender E, Dietze P, Karas H, Knuppel R (1996) TRANSFAC: a database on transcription factors and their DNA binding sites. Nucleic Acids Res 24: 238-241.  103. Wingender E, Karas H, Knuppel R (1997) TRANSFAC database as a bridge between sequence data libraries and biological function. Pac Symp Biocomput: 477-485.  104. Kel AE, Gossling E, Reuter I, Cheremushkin E, Kel-Margoulis OV, et al. (2003) MATCH: A tool for searching transcription factor binding sites in DNA sequences. Nucleic Acids Res 31: 3576-3579.  105. Krause HM, Klemenz R, Gehring WJ (1988) Expression, modification, and localization of the fushi tarazu protein in *Drosophila* embryos. Genes Dev 2: 1021-1036.  106. Han W, Yu Y, Altan N, Pick L (1993) Multiple proteins interact with the *fushi tarazu* proximal enhancer. Mol Cell Biol 13: 5549-5559. | | | |
